# Comparative Experimental and Theoretical Study of Mg, Al and Zn Aryloxy Complexes in Copolymerization of Cyclic Esters: The Role of the Metal Coordination in Formation of Random Copolymers

**DOI:** 10.3390/polym12102273

**Published:** 2020-10-02

**Authors:** Ilya Nifant’ev, Pavel Komarov, Valeriya Ovchinnikova, Artem Kiselev, Mikhail Minyaev, Pavel Ivchenko

**Affiliations:** 1 Department of Chemistry, M.V. Lomonosov Moscow State University, Leninskie Gory 1–3, 119991 Moscow, Russia; 2 A.V. Topchiev Institute of Petrochemical Synthesis RAS, Leninsky Avenue 29, 119991 Moscow, Russia; komarrikov@yandex.ru (P.K.); valeriya.160001@gmail.com (V.O.); metra77@mail.ru (A.K.); mminyaev@mail.ru (M.M.); 3 Faculty of Chemistry, National Research University Higher School of Economics, Miasnitskaya Str. 20, 101000 Moscow, Russia; 4 N.D. Zelinsky Institute of Organic Chemistry RAS, Leninsky pr. 47, 119991 Moscow, Russia

**Keywords:** aryloxy metal complexes, controlled transesterification, DFT, ε-caprolactone, enolization, *L*-lactide, polycaprolactone, polylactide, random copolymerization, ring-opening polymerization

## Abstract

Homogeneity of copolymers is a general problem of catalytic coordination polymerization. In ring-opening polymerization of cyclic esters, the rational design of the catalyst is generally applied to solve this problem by the equalization of the reactivities of comonomers—however, it often leads to a reduction of catalytic activity. In the present paper, we studied the catalytic behavior of BnOH-activated complexes (BHT)Mg(THF)_2_*^n^*Bu (**1**), (BHT)_2_AlMe (**2**) and [(BHT)ZnEt]_2_ (**3**), based on 2,6-di-*tert*-butyl-4-methylphenol (BHT-H) in homo- and copolymerization of *L*-lactide (*l*LA) and ε-caprolactone (εCL). Even at 1:5 *l*LA/εCL ratio Mg complex **1** catalyzed homopolymerization of *l*LA without involving εCL to the formation of the polymer backbone. On the contrary, Zn complex **3** efficiently catalyzed random *l*LA/εCL copolymerization; the presence of mono-lactate subunits in the copolymer chain clearly pointed to the transesterification mechanism of copolymer formation. Both epimerization and transesterification side processes were analyzed using the density functional theory (DFT) modeling that confirmed the qualitative difference in catalytic behavior of **1** and **3**: Mg and Zn complexes demonstrated different types of preferable coordination on the PLA chain (*k*^2^ and *k*^3^, respectively) with the result that complex **3** catalyzed controlled εCL ROP/PLA transesterification, providing the formation of *l*LA/εCL copolymers that contain mono-lactate fragments separated by short oligo(εCL) chains. The best results in the synthesis of random *l*LA/εCL copolymers were obtained during experiments on transesterification of commercially available PLLA, the applicability of **3**/BnOH catalyst in the synthesis of random copolymers of εCL with methyl glycolide, ethyl ethylene phosphonate and ethyl ethylene phosphate was also demonstrated.

## 1. Introduction

**Scheme 1:**
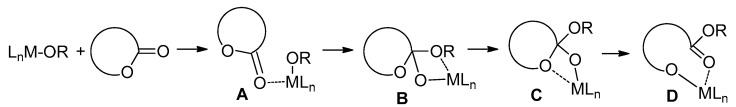
Coordination ring-opening polymerization of cyclic esters: (**A**) monomer coordination; (**B**) nucleophilic addition to carbonyl group; (**C**) coordination of endocyclic oxygen and (**D**) ring-opening.

Catalytic ring-opening polymerization (ROP) of cyclic esters is the basis for the efficient synthetic approach to actual biodegradable and biocompatible materials [1,2,3,4,5,6,7]. Both organocatalysts [8,9,10,11,12,13,14,15] and coordination catalysts [16,17,18,19,20] have been efficiently used in ROP. The reaction mechanism of the coordination ROP, catalyzed by metal alkoxy complexes, is traditionally viewed as a repeating sequence of monomer coordination, alkoxy insertion and ring-opening (Scheme 1); these processes are influenced by both the metal nature and ligand environment at the metal center [16,17,18,19,20,21].

**Scheme 2:**
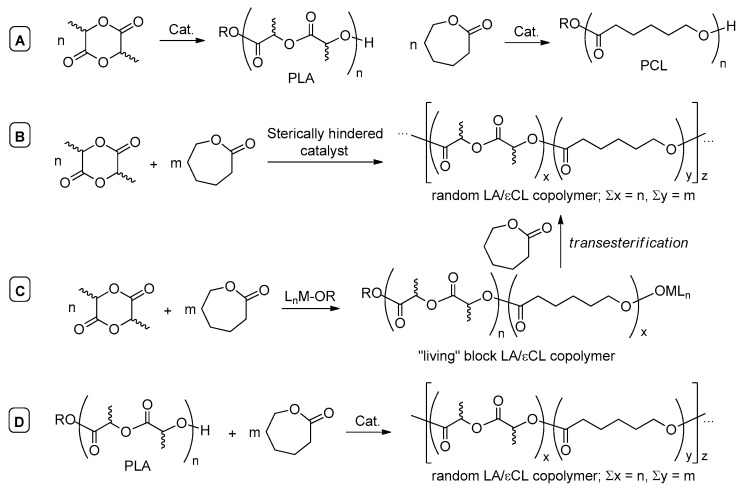
Homopolymerization (**A**) and random copolymerization (**B**) of LA and εCL, formation of random LA/εCL copolymers via transesterification of PLA (**C**,**D**).

Polylactide (PLA) and poly(ε-caprolactone) (PCL), obtained by catalytic ROP of the corresponding cyclic esters, lactide (LA) and ε-caprolactone (εCL) (Scheme 2A) [1,4,8,15,20,22,23,24], have currently received great attention in biomedical applications [25,26,27,28,29,30,31,32]. Due to the contrasting physical properties of polylactide (PLA) and poly-ε-caprolactone (PCL), random copolymers of LA and εCL have generally proved useful for the development of polymer materials with desired mechanical characteristics and biodegradation rate [33,34,35,36,37,38]. However, the inherent difference in reactivity of LA and εCL usually leads to the formation of diblock or gradient copolymers, since in most cases LA was polymerized primarily [39,40,41,42,43,44,45]. Occasionally, only PLA was formed when the mixtures of LA and εCL were used as a comonomer feed [46,47].

Apart from practical use, the formation of random LA/εCL copolymers is of considerable interest for the theory of coordination ROP catalysis. There are basically three approaches to random LA/εCL copolymers. The first approach (Scheme 2B) is to adjust the reactivity ratios of LA and εCL to nearly equal with a formation of the random copolymer in the course of living ROP; this approach can be potentially implemented via the catalyst’s design. This approach had been realized, more or less successfully, when used with sterically hindered complexes of Al [44,48,49,50,51,52,53,54,55,56,57,58,59,60,61], Zn [43,62,63,64,65,66], Ti [67,68,69,70,71,72,73], Zr [70,71], Hf [70], Mo [74], Mg or alkali-earth metals [45,75] and lanthanides [76,77,78]. The formation of random copolymers with the catalysis by metal chelates was usually attributed to higher inhibition of the coordination of LA relative to εCL due to higher steric hindrance for LA coordination [50,79]. This interpretation appears to be incomplete bearing in mind a higher donor number of lactones in comparison with lactides [80,81], a considerable advantage of LA over εCL in coROP can be attributed to the formation of highly stable chelate metal complexes after LA ring-opening that inhibit subsequent ring-opening of εCL [82,83]: this suggestion was confirmed experimentally and theoretically for aryloxy complexes of Mg [47].

The second approach (Scheme 2C) employs transesterification of the initially formed PLA by εCL. This approach should consider the competition between living coordination ROP and transesterification. Similar competition was studied experimentally and theoretically for 1,5,7-triazabicyclo[4.4.0]dec-5-ene catalyzed polymerization of cyclic phosphates [84]. Apparently, transesterification takes place in LA/εCL copolymerization, catalyzed by Al iminophenolates [52], Sn alkoxides [34,37] and Bi derivatives [85,86,87,88]. Not long ago, Yao et al. reported an efficient application of the similar approach in the synthesis of random LA/εCL copolymers from polylactide (PLA) and εCL using La and Yb complexes with chelating ONNO ligands [89]. This third approach (Scheme 2D) to random lactide/lactone copolymers seems to be attractive, but the use of lanthanide catalysts is limited by moderate toxicity of La [90,91] and Yb [92]; additionally, the relative content of monolactate subunits in copolymers was relatively low.

In the recent years, the promising catalytic properties in ROP of different cyclic esters were demonstrated for the complexes of ‘biometals’ (Na, Mg, Ca, Zn and Al) with bulky phenols such as 2,6-di-*tert*-butyl-4-methylphenol (BHT-H) [81,93,94,95,96,97,98,99]. These synthetically available complexes represent a suitable model for the understanding fundamentals of co-ROP [47].

In the present paper, we report the results of the experimental and theoretical study of the catalytic behavior of Mg, Zn and Al phenolates in the synthesis of random copolymers of εCL with *L*-lactide (*l*LA) and other prospective ROP comonomers, bearing in mind underestimated side processes of transesterification and enolization. Our research resulted in the development of an efficient catalytic method of the synthesis of highly statistical biodegradable εCL-based copolymers that are potentially suitable for different applications.

## 2. Materials and Methods

### 2.1. Materials

*n*-Hexane was stored over Na and distilled before use. THF and toluene were refluxed over Na/benzophenone and distilled under argon. *l*LA (Merck, Darmstadt, Germany) was purified by recrystallization and subsequent sublimation in vacuo. εCL (Merck) was distilled before use under argon over CaH_2_. Benzyl alcohol (BnOH, Merck, 99%) was distilled over BaO and stored under argon. Poly(*L*-lactide) (PLLA, *M*_n_ 7.8 × 10^4^ Da) was purchased from FDPlast company (Moscow, Russian Federation). Metal complexes (BHT)Mg(THF)_2_*^n^*Bu (**1**) [97], (BHT)_2_AlMe (**2**) [100] and [(BHT)ZnEt]_2_ (**3**) [101] and cyclic esters 3-methyl-1,4-dioxane-2,5-dione (MeGL) [102], 3-phenyl-1,4-dioxane-2,5-dione (PhGL) [102], 2-ethyl-1,3,2-dioxaphospholane 2-oxide (EtEP) [103] and 2-ethoxy-1,3,2-dioxaphospholane 2-oxide (EtOEP) [104] were synthesized according to previously reported procedures.

### 2.2. Instruments and Methods

The ^1^H and ^13^C NMR spectra were recorded on a Bruker AVANCE 400 or on Bruker Avance III (600 MHz) spectrometers (Bruker Corporation, Billerica, MS, USA). CDCl_3_ (Cambridge Isotope Laboratories, Inc., Tewksbury, MA, USA, D 99.8%) was used as purchased. The chemical shifts are reported in ppm relative to the solvent residual peaks.

Size exclusion chromatography (SEC) of polymer samples was performed using an Agilent PL-GPC 220 chromatograph equipped with a PLgel column (Agilent Technologies, Santa Clara, CA, USA) and THF was used as the eluent (1 mL/min). The measurements were recorded with universal calibration based on a polystyrene standard at 40 °C.

Differential scanning calorimetry (DSC) experiments were performed on the TGA/DSC1 apparatus (Mettler Toledo, Columbus, OH, USA).

### 2.3. Preparation and X-ray Diffraction Study of the Complex 4

Zn complex **4** was prepared using the previously reported method [105]. Single crystals of the complex **4** were obtained by low-temperature crystallization from hexane/THF solution. X-ray diffraction data were collected at 100 K on a Bruker Quest D8 diffractometer using Mo*K*_α_ radiation (λ = 0.71073 Å). The intensity data were integrated by the SAINT program [106] and corrected for absorption and decay using SADABS [107]. The structure was solved by direct methods using SHELXT [108] and refined on *F^2^* using SHELXL-2018 [109]. All non-hydrogen atoms were refined with anisotropic displacement parameters. Hydrogen atoms were placed in ideal calculated positions and refined as riding atoms with relative isotropic displacement parameters. The Mercury program [110] was used for molecular graphics. The structure has been deposited at the Cambridge Crystallographic Data Center (CCDC) with the reference CCDC number 2021607; it also contains the supplementary crystallographic data. These data can be obtained free of charge from the CCDC [111]. Crystal data, data collection and structure refinement details for **4** are summarized in Table S1 in the Supplementary Materials, bond lengths are presented in Table S2 in the Supplementary Materials.

### 2.4. Polymerization Experiments

#### 2.4.1. Homopolymerization of *l*LA and εCL

*l*LA (1.44 g, 10 mmol; Table 1, Entries 1–6) or εCL (1.14 g, 10 mmol; Table 1, Entries 7–12) was placed under argon into a flame-dried vial. Toluene (7 mL) was added and the mixture was heated with stirring to the target temperature (oil bath). In a separate vial, the precatalyst (**1**–**4**, 0.1 mmol) was dissolved in toluene (0.5 mL) and BnOH (10.8 mg, 0.1 mmol) was added. After 1 min, this mixture was added through the septum to the first vial, and the total volume of the mixture was latched to 10 mL. After 2 h, 0.1 mL of AcOH was added. After sampling of the organic phase, CH_2_Cl_2_ (10 mL) was added, the organic phase was poured into MeOH (150 mL). The product was separated by centrifugation, washed by MeOH (3 × 10 mL), hexane (10 mL) and dried in vacuo. Conversion of monomers and composition of the copolymers was determined by ^1^H NMR spectroscopy, from the ratio of the integrated values of the CH_2_O signals of the εCL (m, 4.2 ppm) and PCL units (t, 4.07–4.14 ppm) as well as methine signals of *l*LA (4.75 ppm) and PLA units (5.05–5.25 ppm; see Section 3.4). The ^1^H spectra of the samples are presented in the Supplementary Materials.

#### 2.4.2. Copolymerization of *l*LA and εCL

The experiments were performed by the same manner, using *l*LA (0.72 g, 5 mmol) and εCL (0.57 g, 5 mmol; Table 2, Entries 1–6) or *l*LA (0.72 g, 5 mmol) and εCL (2.85 g, 25 mmol; Table 2, Entries 7–10) loading. The sampling of the reaction mixture was done after 1, 3 and 5 h. Separation of copolymers was made as described in Section 2.4.1. ^1^H and ^13^C NMR spectra of copolymers are presented in the Supplementary Materials.

#### 2.4.3. Synthesis of Random *l*LA/εCL Copolymers Using PLLA

The experiments on transesterification were performed using commercial PLLA (*M*_n_ 7.8 × 10^4^ Da, 0.72 g, 5 mmol of dilactate) and εCL (2.85 g, 25 mmol; Table 3, Entries 1 and 2) loading. The sampling of the reaction mixture was done after 5 and 15 h. Separation of copolymers was made as described in Section 2.4.1. ^1^H and ^13^C NMR spectra of copolymers are presented in the Supplementary Materials.

#### 2.4.4. Copolymerization of εCL with Other Comonomers

In these experiments (Table 3, Entries 3–6) we used 5 mmol loadings of comonomers (0.65 g for MeGL, 1.03 g for MePhGL, 0.68 g for EtEP and 0.76 g for EtOEP), the loading of εCL remained unchanged (2.85 g, 25 mmol). The sampling of the reaction mixture was done after 1, 3 and 5 h. Separation of copolymers was made as described in Section 2.4.1. ^1^H and ^31^P NMR spectra of copolymers are presented in the Supplementary Materials.

### 2.5. DFT Calculations

Molecular structures of stationary points and transition states were optimized using the density functional theory (DFT). The initial Cartesian coordinates of the stationary points had been drafted by PRIRODA software [112] using the 3ζ basis. The final calculations (structure optimization and determination of the thermodynamic parameters) were carried out using the Gaussian 09 program [113] for the gas phase at 298.15 K. The B3PW91 hybrid exchange-correlation functional [114,115] and DGTZVP basis [116,117] were used in the optimizations, the RMS force criterion was 3 × 10^−4^. More detailed data (molecular plots, Cartesian coordinates and energy characteristics of the optimized structures) are presented in the Supplementary Materials.

## 3. Results and Discussion

### 3.1. Homopolymerization of lLA and εCL

**Scheme 3:**
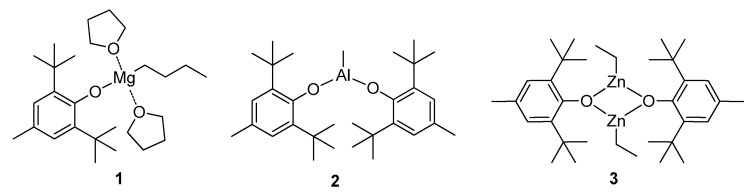
Metal complexes **1**–**3** studied in homo- and copolymerization of *l*LA and εCL.

To estimate the catalytic performance of the BHT derivatives of Mg, Zn and Al, we chose the complexes (BHT)Mg(THF)_2_*^n^*Bu (**1**) [97], (BHT)_2_AlMe (**2**) [100] and [(BHT)ZnEt]_2_ (**3**) [101] (Scheme 3). In our experiments on homopolymerization of lLA and εCL, we used activation of alkyl complexes **1**–**3** by BnOH.

At room temperature only complex **1** proved to be an efficient initiator of the ROP of *l*LA, complex **2** was inactive, **3** demonstrated very low activity (Table 1, Entries 1–3, respectively). When the temperature was increased to 100 °C, Zn complex **3** efficiently catalyzed the formation of PLLA (Table 1, Entry 6), but Al complex **2** demonstrated low activity. In marked contrast, the complex **2** (along with **1**, see Figure S1 in the Supplementary Materials) was active at 20 °C in homopolymerization of *ε*CL (Table 1, Entries 7 and 8), while Zn complex **3** did not show any activity (Table 1, Entry 9). At 100 °C high conversion of *ε*CL was achieved after 2 h for all complexes (Table 1, Entries 10–12, see Figure S2 in the Supplementary Materials for **3**).

**Figure 1:**
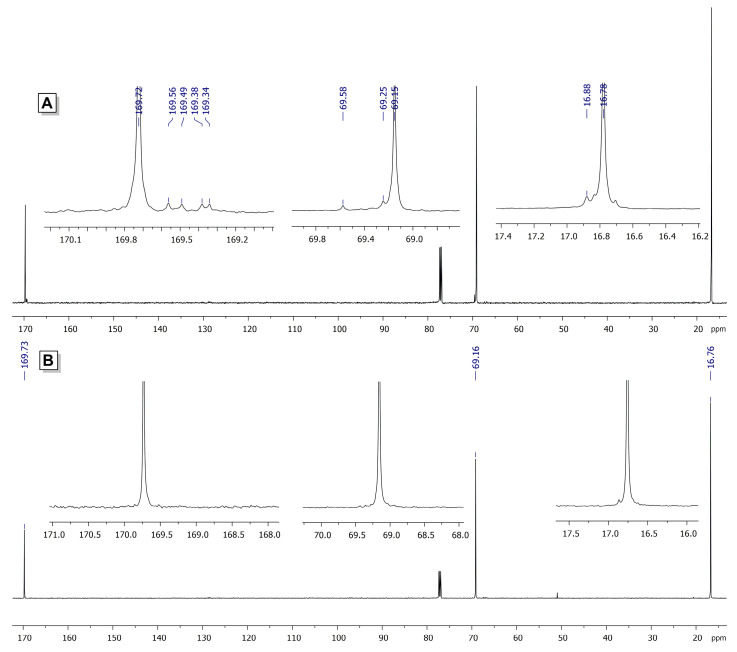
^13^C NMR spectra (CDCl_3_, 20 °C) of PLLA obtained in the presence of **1** (**A**) and **3** (**B**).

The samples of PLLA (Table 1, Entries 1, 6 and 4) demonstrated substantially different thermal properties. The value of the melting point (*T*_m_) of PLLA, obtained in the presence of **1** at ambient temperature (Table 1, Entry 1), was 161.4 °C, which is comparable with *T*_m_ = 165.2 °C of PLLA, obtained in the presence of Zn catalyst **3** at 100 °C (Table 1, Entry 6, Figure S3B in the Supplementary Materials). However, *T*_m_ of PLLA, synthesized in the presence of **1** at 100 °C (Table 1, Entry 4), was 147.1 °C (Figure S3A in the Supplementary Materials). We proposed that such behavior might be caused by partial disorder in the PLLA microstructure. Analysis of the ^13^C NMR spectra of PLLA samples 4 and 6 (Table 1) confirmed our assumption. In the region of the signals of carbonyl groups for PLLA, obtained in the presence of **1**/BnOH, we observed additional signals that can be attributed to stereoerrors in isotactic PLLA chain (Figure 1). We propose that partial epimerization of CHMe fragments can be explained by enolization of the PLLA chain under the influence of the active BHT-Mg-OR species (see Section 3.3).

### 3.2. Copolymerization of lLA and εCL

To compare catalytic behavior of the complexes **1**–**3** in copolymerization of *l*LA and εCL, we performed a series of experiments using the 1:1 comonomer ratio (Table 2, Entries 1–6). At 20 °C, after activation of Mg complex **1** by BnOH we observed fast polymerization of *l*LA, while εCL did not form a polymer even in trace amounts. Under these conditions, Al complex **2** was inert, Zn complex **3** catalyzed very slow formation of *l*LA oligomer without polymerization of εCL. At 100 °C, Mg complex **1** continued to be inactive towards εCL. Similar to the homopolymerization experiment (Table 1, Entry 4), the value of *T*_m_ of PLLA (154.2 *T*_m_ of PLLA) was substantially lower than the reference value (165 °C). Aluminum complex **2** at 100 °C (Table 2, Entry 5) formed low molecular weight PLLA, εCL was not polymerized. The most interesting preliminary results on *l*LA/εCL copolymerization at 100 °C were obtained for Zn complex **3** (Table 2, Entry 6). ^1^H NMR spectrum of the reaction mixture demonstrates the presence of relatively long PLLA fragments and, on the other hand, PCL fragments were absent (no CH_2_C(O) signals at 2.3 ppm). Therefore, the product of the reaction was a random, not gradient, *l*LA/εCL copolymer. This conclusion is also supported by a large decline of *T*_g_ and *T*_m_ values (18.5 °C and 133.8 °C, respectively, see Figure S4 in the Supplementary Materials) in comparison with the reference values for PLLA (60–65 °C and 165 °C, respectively).

Thus, Mg and Zn BHT complexes **1** and **3** demonstrated qualitatively different catalytic behavior. The complex **1**/BnOH, being a highly active catalyst of the homopolymerization of *l*LA and εCL, was unable to polymerize εCL in the presence of lactide even at elevated temperatures. In contrast with **1**, the use of the zinc catalyst **3**/BnOH allowed one to obtain random *l*LA/εCL copolymers, although with low content of 6-oxyhexanoate fragments. Aluminum complex **2** was far below both **1** and **3** by catalytic activity.

To improve confidence in our evaluation of the catalytic behavior of **1**–**3** and in order to obtain random *l*LA/εCL copolymers with higher 6-oxyhexanoate content, we made a series of copolymerization experiments at 100 °C using the 1:5 *l*LA/εCL ratio (Table 2, Entries 7–9). Under these conditions, **1**/BnOH continued to polymerize *l*LA but with a much reduced rate (Table 2, Entry 7), copolymerization of εCL was detected only in trace amounts after 15 h. *T*_m_ of PLLA obtained after 15 h was 134.6 °C. Conversion of *l*LA in the presence of **2**/BnOH after 1 h was only 30% (Table 2, Entry 8a), however, minimal insertion of εCL was detected. After 15 h, the full conversion of *l*LA and nearly complete conversion of εCL were achieved (Table 2, Entry 8d).

Zinc complex **3** has proven to be much more efficient in the synthesis of *l*LA/εCL copolymer in comparison with Al complex **2**. After 1 h, after nearly complete polymerization of *l*LA, the conversion of εCL amounted to more than 20% (Table 2, Entry 9a). After 15 h, the conversion of εCL was almost completed with a formation of highly statistical copolymer (Table 2, Entry 9d). *T*_m_ of this copolymer was 35.7 °C (see Figure S5 in the Supplementary Materials); the closeness of this value to the human body temperature deserves special attention in the context of possible biomedical applications of random *l*LA/εCL copolymers. It is quite obvious that the formation of *l*LA/εCL copolymers when using **2** and **3** is due to transesterification of the initially formed PLLA in combination with polymerization of εCL.

### 3.3. Synthesis, Molecular Structure and Catalytic Behaviour of the Zn Chelate Complex 4

**Scheme 4:**
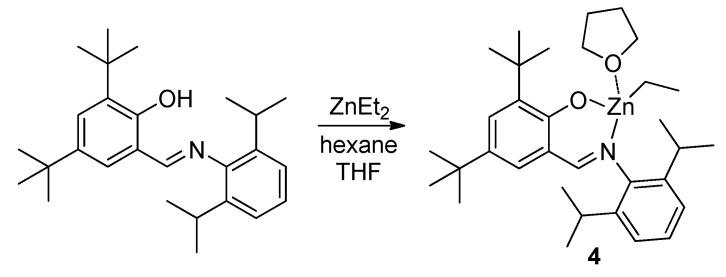
Synthesis of zinc complex **4**.

Lower catalytic activity in both polymerization and transesterification of **2** in comparison with **3** can be explained not only by the nature of the metal (Al or Zn), but also by the qualitatively different ligand environment in Al complex **2** containing two bulky BHT ligands that occupy two coordination sites. To evaluate the impact of the additional metal–ligand bonding on the catalytic behavior of the zinc complexes in ROP, we prepared chelate complex **4** (Scheme 4).

**Figure 2:**
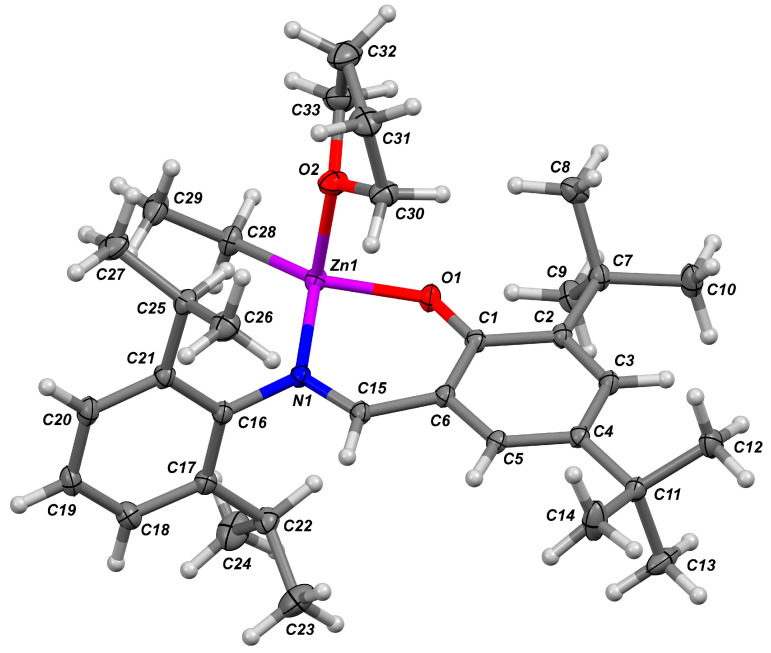
Molecular structure of the complex **4**. The probability for thermal ellipsoids is set to the 50% level.

The complex **4** was obtained earlier by Darensbourg et al. in aliphatic reaction media [105]. To establish the ability of the Zn atom to binding with donor ROP substrates, we prepared single crystals of **4** by low-temperature crystallization from hexane solution in the presence of donor solvent, THF. X-ray diffraction analysis of **4** (Figure 2, see also Figure S6 in the Supplementary Materials for ORTEP drawing) confirmed the monomeric nature of the complex, we found that the Zn atom was bonded to one THF molecule. The Zn environment in **4** was best described as a distorted trigonal pyramid (coordination number is 4; see Table 4 for Zn-L bond distances), where Zn was located nearly within the plane defined by atoms O1, N1 and C28 (the deviation was 0.3096(9) Å). Atoms C1–C6 and C15 were also situated in the same plane. Such an unusual Zn environment is mainly caused by the presence of the flat fragment in the C_27_H_38_NO ligand and by the steric influence of the bulky (2,6-^i^Pr_2_C_6_H_4_) substituent located close to the Zn atom.

In *l*LA/εCL copolymerization, the complex **4**/BnOH (Table 2, Entry 10) has been less active in comparison with **3**/BnOH. The almost complete conversion of *l*LA was observed in one hour, however, the conversion of εCL was only 3%. After 5 h, 78% of εCL polymerized, but the polymer mostly comprised of long PLLA and PCL fragments (see Section 3.4). We thought that the low activity of **4** in transesterification is attributable to bidentate nature of the iminophenolate ligand hampering the coordination of PLLA chain at Zn atom (see Section 3.5).

### 3.4. Microstructure and Thermal Properties of lLA/εCL Copolymers

**Figure 3:**
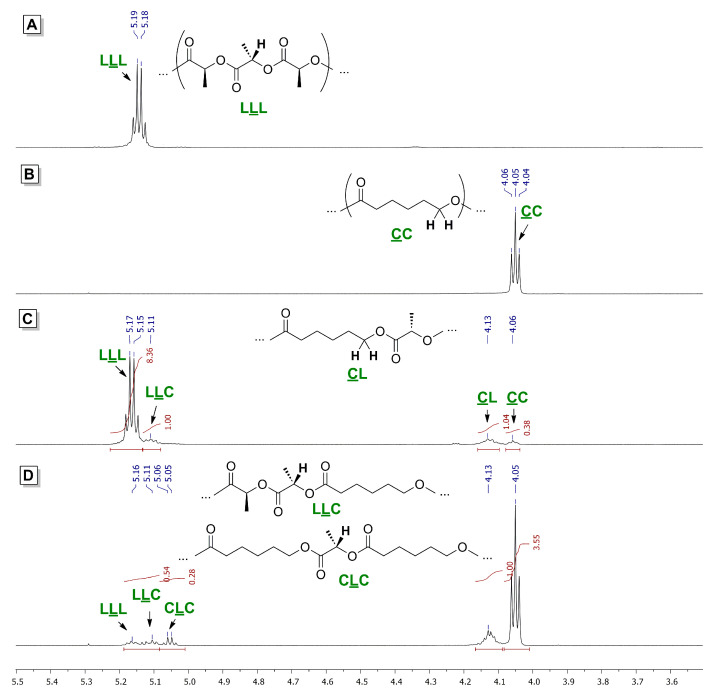
^1^H NMR spectra (CDCl_3_, 20 °C, 600 MHz) of PLLA (**A**), PCL (**B**) and *l*LA/εCL copolymers obtained at the 1:5 *l*LA/εCL ratio using complexes **1** (**C**) and **3** (**D**).

The microstructure of *l*LA/εCL copolymers was analyzed by ^1^H and ^13^C NMR spectroscopy. ^1^H NMR spectra of homo- and copolymers of *l*LA and εCL are presented in Figure 3. In these spectra, a few significant signals could be distinguished, making it possible to determine both comonomer ratios and some comonomer sequences. The first group is related to the signals of methine proton in lactate fragments. These signals are quadruplets with characteristic chemical shifts that can be attributed to LLL, LLC and CLC fragments (δ = 5.17, 5.11 and 5.05 ppm, respectively). Note that the presence of the signal of CLC fragment clearly points to the transesterification of PLLA chain during copolymerization, the share of these mono-lactate fragments in the PLLA fraction of the copolymer is presented in Table 2. The second characteristic group of the signals is triplets related to the –CH_2_O– group of 6-oxyhexanoate fragments. Triplet at 4.13 ppm is related to the CL fragment, triplet at 4.05 ppm is a signal of methylene protons of the CC fragment. The ratio of the integral intensities of these signals allows one to determine the average sequence length (ASL) of PCL fragments in the copolymer by the ratio: ASL_C_ = (*I*_C__C_ + *I*_C__L_)/*I*_C__L_ (see Table 2).

**Figure 4:**
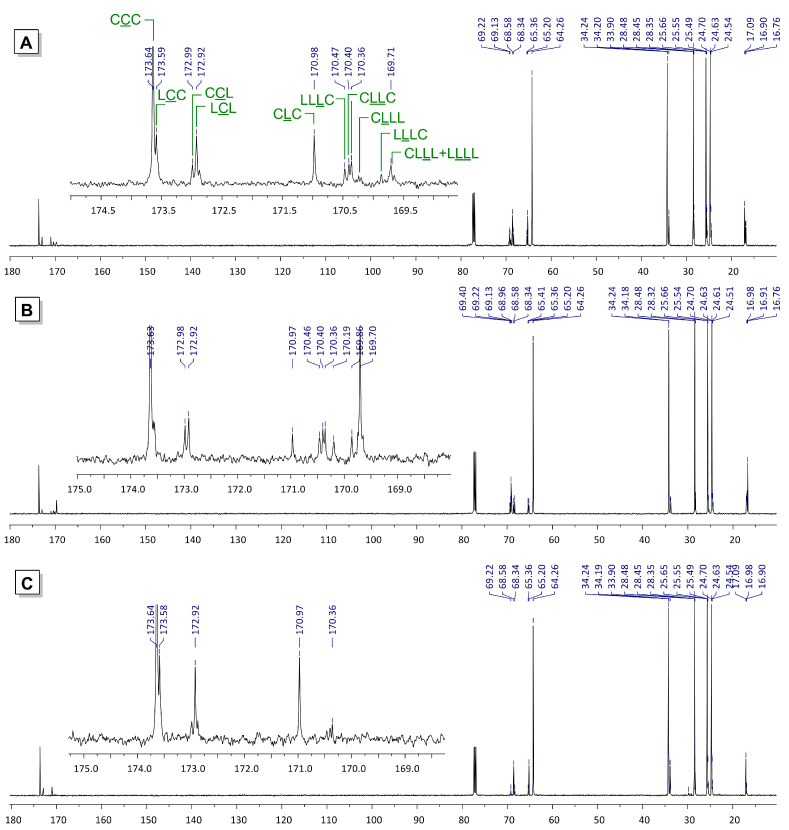
^13^C NMR spectra (CDCl_3_, 20 °C, 151 MHz) of the products of *l*LA/εCL copolymerization using **3** (**A**) and **4** (**B**) as a catalysts, and transesterification of PLLA by εCL in the presence of **3** (**C**).

Detailed and consistent analysis of ^13^C NMR spectra of *l*LA/εCL copolymers was reported earlier, the most suitable interval for the detailed analysis of the copolymer’s microstructure is the area 169–174 ppm of the signals of carbonyl groups [42,49,52,53,61,62,72]. The view of the ^13^C NMR spectra of *l*LA/εCL copolymers (Figure 4A,B) confirmed the difference in catalytic behavior of the Zn complexes **3** and **4**. The presence of the signal of CLC triads at 171 ppm resulting from the cleavage of the lactyl–lactyl bond in the dilactate unit [51] is a criterion for the transesterification during ROP. This signal presents in both spectra, however, it is this signal that is most intensive for the copolymer, obtained in the presence of **3**. In contrast, during catalysis by chelate complex **4**, longer oligolactate and oligo(6-oxyhexanoate) fragments were formed, which confirms the lower efficiency of **4** as a transesterification catalyst.

Another important point is the relative high intensity of the LCL signal in the ^13^C NMR spectrum of copolymer, formed in the presence of **3**. High content of LCL fragments reflects a high probability of transesterification of the PLLA chain after one single coordination and ring-opening of the εCL molecule. This important feature is discussed below from the point of view of the reaction mechanism (see Section 3.5). A random character of the copolymer can be confirmed by the value of the glass transition point *T*_g_ that is predicted by the empirical Fox Equation (1) [40],

(1) $$\frac{1}{T_{g}}=\frac{W_{1}}{T_{g1}}+\frac{W_{2}}{T_{g2}}$$

where *T*_g1_ and *T*_g2_ are glass transition points of homopolymers (60–65 °C for PLLA and −65 to −60 °C for PCL), *W*_1_ and *W*_2_ are mass fractions of the comonomers in the copolymer. For the LA/εCL copolymer with a relatively low εCL rate (Table 2, Entry 6) the DSC curve displayed a glass transition temperature at 18.5 °C, which was close to the theoretical value for the random LA/εCL copolymer (20 °C for 26% mol εCL). For LA/εCL copolymers obtained at the 1:5 LA/εCL molar ratio, calculated *T*_g_ was –46 °C. The experimental DSC curve of the copolymer, obtained using this comonomer ratio in the presence of **3** (Table 2, Entry 9c), comprised of a crystallization peak at 9.6 °C and melting peak at 33.8 °C, glass transition was not detected down to −50 °C (see Figure S5 in the Supplementary Materials).

### 3.5. Mechanistic Insights of the Formation of lLA/εCL Copolymers

Recently, we studied BHT-Mg catalyzed ROP of different cyclic substrates at the B3PW91/DGTZVP level of the density functional theory (DFT); to reduce the calculation time, 2,6-di-*tert*-butylphenoxy (DBP) complexes were applied as initiator models [47,81,118,119]. In the present work, we also used DBP derivatives of Mg and Zn as a model catalytic species and transition states, derived from **1** and **3**, respectively, and chose the same B3PW91/DGTZVP basis set to compare new and previously obtained results. Polymerization experiments have shown that Al complex **2** demonstrated interim characteristics between **1** and **3**, and we ruled out this complex from the theoretical investigations.

**Scheme 5:**
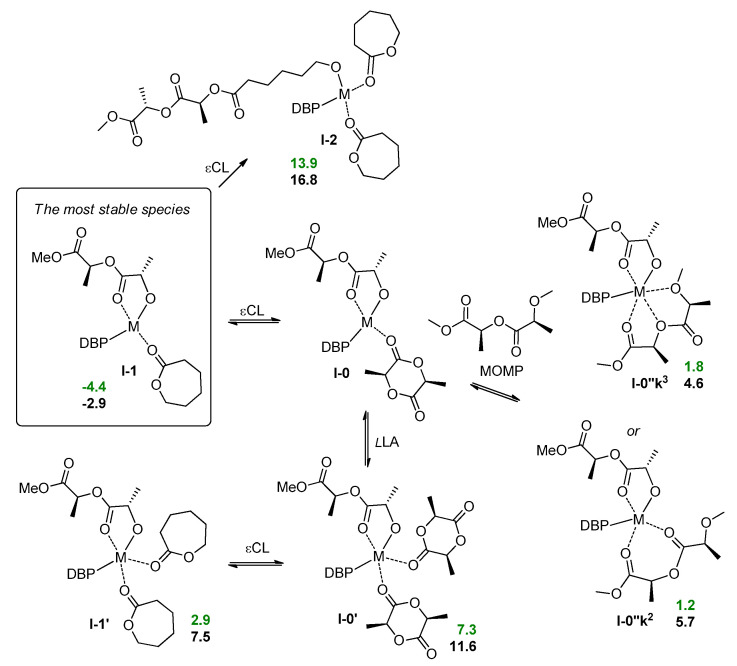
Calculated thermodynamic data for ligand exchange and εCL ring-opening during *l*LA/εCL copolymerization. The values of the free energies (kcal/mol) relative to **I-0** are colored by green for Mg and by black for Zn.

Copolymerization experiments have shown that under the mild conditions in the mixture of *l*LA and εCL after activation by the BnOH Mg complex **1** catalyzed formation of PLLA without marked polymerization of εCL. The Zn complex **3** was able to catalyze copolymerization, but only at elevated temperature. In the modeling of copolymerization and transesterification we considered tetrahedral metal complexes, stabilized by the formation of five-membered chelate, as a starting stationary points. The reaction mixture contained three components that can coordinate the metal center, namely, *l*LA, εCL and PLA. For the simulation of the PLA fragment we used (*S*,*S*)-MeOC(O)CHMeOC(O)CHMeOMe ((*S*,*S*)-1-methoxy-1-oxopropan-2-yl 2-methoxypropanoate, MOMP, Scheme 5) and found two possible coordination modes for MOMP. Our calculations showed that the *k*^2^-coordinated MOMP complex was more stable for Mg; in the case of Zn *k*^3^-coordination is preferable. This finding is highly important for the analysis of transesterification and other side processes (see below).

Tetrahedral complexes with εCL **I-1** seemed to be low-energy ground states for *l*LA/εCL copolymerization. Starting from these complexes, we analyzed the change of the free energies and free enthalpies for the ring-opening of εCL molecule, and found that the formation of the product **I-2** (Scheme 5) is an endergonic process both for Mg and Zn. Therefore, the ring-opening of the first εCL molecule after polymerization of *l*LA needs for the compensation, and the formation of stable chelate, metal lactate complex, in further transformations appears to be a favorable reaction pathway. Additionally, note that the presence of εCL in the reaction mixture should slow down ROP of *l*LA by the mechanism of concurrent inhibition. This conclusion is supported by the results of our experiment on *l*LA/εCL copolymerization at the 1:5 comonomer using **1**/BnOH as a catalyst (Table 1, run 7).

**Scheme 6:**
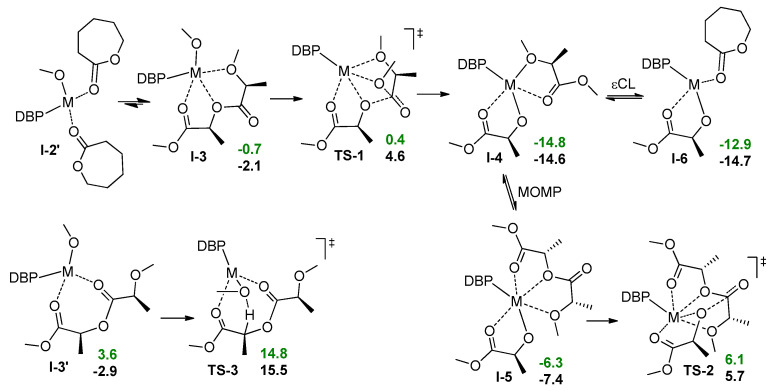
Calculated thermodynamic data for ligand exchange and transesterification during *l*LA/εCL copolymerization. The values of the free energies (kcal/mol) relative to **I-2’** are colored by green for Mg and by black for Zn.

**Figure 5:**
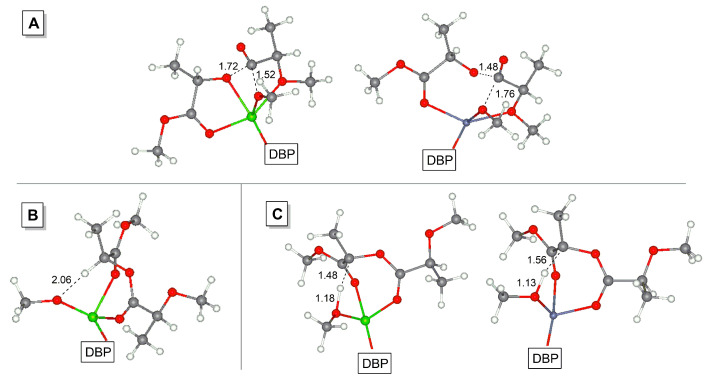
Optimized geometries of the transition states of PLA transesterification **TS-1** (**A**) and enolization **TS-3** (**C**) for M = Mg (left) and Zn (right); close contact in Mg-MOMP complex **I-3’** (**B**). Interatomic distances are given in Å.

In theory, following ring-opening of εCL in **I-2** may offset the endergonic effect after the first insertion and ring-opening of the first εCL molecule. However, the coordination of εCL in the reaction mixture is competing with coordination of the PLA chain. Using a simplified model (MeO fragment instead of ring-opened εCL, Scheme 6) we optimized the geometries of DBP-metal complexes with two εCL molecules and with MOMP (PLA model) in the *k*^3^-coordination mode (Scheme 6, **I-2’** and **I-3**, respectively) and found MOMP coordination to be an exergonic process. We have therefore tried to analyze the possible ways of the transformations of the complexes **I-3** with MOMP, and suddenly found transition states **TS-1** with unusual geometry (Figure 5A): in contrast with conventional TS of the first stage of coordination ROP, in **TS-1** the oxygen atom of the reactive carbonyl group is not coordinated at the metal center. Going through minimal activation barriers (1.1 and 6.8 kcal/mol for Mg and Zn, respectively), the MOMP complexes transform into transesterification products **I-4**, this process is highly exergonic.

Obviously, the complexes **I-4** are still reactive. First, these complexes can undergo further transesterification. We replaced (*S*)-methyl 2-methoxypropanoate in **I-4** by MOMP obtaining the complex **I-5** (Scheme 6) and calculated the activation barriers of transesterification of PLA by lactate via **TS-2** (Scheme 6) amounting to 12.4 and 13.1 kcal/mol for Mg and Zn, respectively. Hence, in the absence of lactide, the catalytic process may continue as transesterification of PLA, the living ROP turns into immortal fragmentation of PLA, accompanied by broadening of molecular weight distribution (MWD). Second, (*S*)-methyl 2-methoxypropanoate can be replaced by the εCL molecule with a formation of **I-6** (Scheme 6). Such a replacement was found to be substantially endergonic for Mg and slightly exergonic for Zn.

In that way, the results of our calculations predicted qualitatively different behavior for Mg and Zn complexes in the mixture of LA and εCL. The Mg complex, after polymerization of LA, continues to be a moderately active catalyst of transesterification of PLA under the action of the lactate fragment, resulting in PLA with broader MWD as a single reaction product. Zn complex that retains the ability to coordinate εCL molecule, involves εCL to transesterification that includes intermediate ring-opening of lactone. This process results in the formation of random LA/εCL copolymer.

In concluding the discussion of the results of DFT modeling, we wish to draw attention to another important issue, namely, epimerization of PLLA during polymerization. We mentioned above that MOMP can coordinate metal atoms in *k*^2^- and *k*^3^-coordination modes (Scheme 5), the first mode was preferable for the Mg complex. We optimized the geometries of the complexes **I-3’** with the formula (DBP)M(OMe)(*k*^2^-MOMP) (M = Mg, Zn) and found the presence of promising MeO–HC(Me) contact in the Mg complex (d = 2.06 Å, Figure 5B); such contact was absent in the Zn complex. The transition states that correspond to the enolization process **TS-3** (Figure 5C) were found by scanning of C–H bond distances, the free activation energies were 11.2 and 18.4 kcal/mol for Mg and Zn, respectively. Taking into account substantial difference in **TS-3** for Mg and Zn, such enolization seems to be feasible for Mg derivative. In addition, the presence of the MeO–HC(Me) contact in starting the Mg complex **I-3’** should facilitate enolization due to a high Arrhenius factor.

Optimization of the Mg lactate complex (DBP)M(OCHMeC(O)OMe)(*k*^2^-MOMP) **I-3’’** (as even more specific model complex for the study of enolization during *l*LA homopolymerization) also detected MeO–HC(Me) contact (d = 2.10 Å), the free activation energy of the enolization via **TS-3’’** was 17.6 kcal/mol (see Supplementary Materials for details). Therefore, one would expect to observe epimerization during *l*LA polymerization, catalyzed by the BHT-Mg catalyst **1**. This conclusion is in good agreement with ^13^C NMR spectral data and DSC analysis of PLLA samples obtained in the presence of **1**.

### 3.6. Synthesis of Random εCL Copolymers Using PLLA and Other Comonomers

**Scheme 7:**
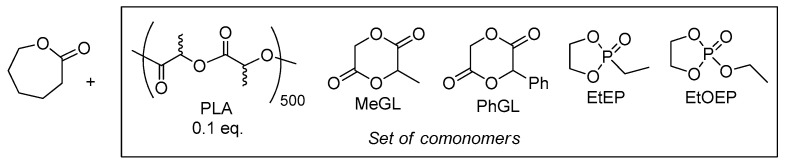
Comonomers studied in the synthesis of random copolymers with εCL in the presence of **3**/BnOH.

Since the formation of random *l*LA/εCL copolymers in the presence of **3** follows homopolymerization of *l*LA even at low *l*LA/εCL ratios (Table 2, Entry 9), we proposed that these copolymers could be efficiently synthesized from PLLA (Scheme 7). In our experiment (Table 3, Entries 1,2), we used commercial PLLA containing 500 dilactyl subunits (*M*_n_ 7.8 × 10^4^ Da), the ratio LL/εCL/**3**/BnOH was 50:250:1:1. The reaction was conducted at 100 °C (Table 2, Entries 7–10). After 15 h the reaction was completed, the product contained about 2/3 of lactate subunits as a CLC fragments (see Figure 4C). The average sequence length of PCL fragment was 6, which confirms highly statistical character of the copolymer (see also Figures S7–S9 in the Supplementary Materials).

We proposed that substituted glycolides are also able to form random copolymers with εCL, and studied copolymerization of εCL with two monosubstituted glycolides, namely, MeGL and PhGL (Scheme 7). The reaction with MeGL after 5 h resulted in the formation of the random copolymer (ASL_C_ = 3.6, Table 3, Entry 3c, see Figures S10 and S11 in the Supplementary Materials). During the reaction with PhGL (Table 3, Entries 4a–c) we observed a fast formation of low molecular weight poly(PhGL) and homopolymerization of PCL, bimodal MW distribution of the reaction products was confirmed by SEC. We were assuming that failure in the synthesis of PhGL/εCL copolymer was owing to the formation of Zn enolates, stabilized by conjugation with phenyl groups.

Since phosphate- and phosphonate-containing polymers were of considerable interest for biomedical applications due to biocompatibility and biodegradability [120,121,122,123,124], we studied copolymerization of εCL with two substituted 1,3,2-dioxaphospholane 2-oxides, EtEP and EtOEP (Scheme 7). The fast formation of the random copolymer was detected when EtEP was used as the comonomer (Table 3, Entries 5a–c). This copolymer was formed within 1 h, and there was no significant change in the view of ^1^H and ^31^P NMR spectra of the reaction mixture for an additional four hours (see Figures S12–S14 in the Supplementary Materials). Copolymerization of εCL with EtOEP occurred in a similar manner, but with lower εCL conversions (Table 3, runs 6a–c), the formation of the random copolymer has been confirmed by ^1^H, ^13^C and ^31^P NMR spectra (see Figures S15–S17 Supplementary Materials).

## 4. Conclusions

Aryloxy complexes of Mg, Al and Zn demonstrated different catalytic behavior in homopolymerization and copolymerization of *l*LA and εCL. BHT-Mg complex **1**, being a highly efficient catalyst of homopolymerization of both *l*LA and εCL, was not suitable for the synthesis of random copolymers. As an example, for the mixture of *l*LA and εCL in the presence of **1**/BnOH we observed slowed-down homopolymerization of *l*LA, almost without εCL polymerization. In addition, in the presence of **1** we detected substantial epimerization of PLLA during *l*LA homopolymerization. BHT-Zn complex **3** at elevated temperatures efficiently catalyzed homopolymerization of *l*LA and εCL, and furthermore, polymerization of *l*LA was not accompanied by side reactions. The complex **3** has proved to be efficient catalyst for the synthesis of a random *l*LA/εCL copolymer, the presence of mono-lactate fragments in the copolymer backbone clearly indicates that the formation of the copolymer occurs by the transesterification mechanism. Since the feasibility of such a mechanism depends on the ability of the metal atom to bonding with the PLA chain, we studied catalytic activity of Zn iminophenolate **4** in *l*LA/εCL coROP, establishing a negative impact of additional ligand coordination on the catalytic activity of the Zn complex. The fundamental difference in catalytic behavior of **1** and **3** was verified using DFT simulation of *l*LA/εCL copolymerization and related processes of transesterification and enolization of PLLA. The difference in preferable PLA coordination modes, *k*^2^-coordination for Mg and *k*^3^-coordination for Zn, explained the difference in the catalytic behavior of the complexes, namely, PLLA enolization for **1** and εCL ring-opening/PLLA transesterification for **3**.

Copolymers, obtained by coROP of *l*LA and εCL at the 1:5 comonomer ratio, had a high content of CLC fragments, which was increased up to 2/3 when PLLA was used as a source of lactate. Hence, we developed an efficient method of the synthesis of εCL-based *l*LA/εCL copolymers, which allowed us to introduce more hydrophobic lactate fragments “by the piece”.

By replacing *l*LA with comonomers of glycolide and cyclophosphate families, we successfully obtained random copolymers of εCL with MeGL, EtEP and EtOEP as novel prospective biodegradable materials.

## Figures and Tables

**Table 1:** Homopolymerization results for complexes **1**–**3** (1 M monomer solutions in toluene, [Mon]/[Precat]/[BnOH] ratio 100:1:1, 2 h).

Entry	Precat.	Monomer	T, °C	Conv., %	*M*_n_ (theo), ×10^3^ Da ^1^	*M*_n_ (SEC), ×10^3^ Da ^2^	*Đ*_M_^2^
1	**1**	*l*LA	20	>99	14.52	12.52	1.97
2	**2**	*l*LA	20	0	-	-	-
3	**3**	*l*LA	20	5	-	-	-
4	**1**	*l*LA	100	>99	14.52	13.32	1.85
5	**2**	*l*LA	100	21	-	-	-
6	**3**	*l*LA	100	>99	14.52	12.54	2.03
7	**1**	*ε*CL	20	>99	11.41	13.50	1.40
8	**2**	*ε*CL	20	>99	11.41	24.29	1.49
9	**3**	*ε*CL	20	0	-	-	-
10	**1**	*ε*CL	100	>99	11.41	6.92	1.50
11	**2**	*ε*CL	100	94	10.73	8.11	1.55
12	**3**	*ε*CL	100	99	11.30	8.30	2.19

^1^ Calculated by the formula *M*_n_ (theo) = *M*(monomer) × Conv. + 108.14, where 108.14 is *M* of the initiator BnOH. ^2^ Determined by SEC *vs* polystyrene standards and corrected by a factors of 0.56 (PCL) and 0.58 (PLA).

**Table 2:** LA/εCL copolymerization results for complexes **1**–**3**.

Entry	Pre-Cat.	*l*LA/εCL/ Cat/BnOH Ratio	[*l*LA], mol/L	[εCL], mol/L	T, °C	t, h	Conversion, % ^1^		*M*_n_ (theo), ×10^3^ Da ^2^	*M*_n_ (SEC), ×10^3^ Da ^3^	*Đ*_M_^3^	CLC, % ^4^	ASL_C_^5^
							*l*LA	εCL					
1	**1**	50/50/1/1	0.5	0.5	20	2	>99	0	7.10	8.30	1.57	0	-
2	**2**	50/50/1/1	0.5	0.5	20	2	0	0	-	-	-	0	-
3	**3**	50/50/1/1	0.5	0.5	20	2	~5	0	-	-	-	0	-
4	**1**	50/50/1/1	0.5	0.5	100	2	>99	0	7.10	9.70	1.48	0	-
5	**2**	50/50/1/1	0.5	0.5	100	2	25	0	-	-	-	0	-
6	**3**	50/50/1/1	0.5	0.5	100	2	99	36	8.27	13.15	1.45	0	~1.0
7a	**1**	50/250/1/1	0.5	2.5	100	1	83	0	6.09	5.77	1.28	0	-
7b						3	86	0	6.31	5.46	1.44	0	-
7c						5	87	0	6.38	7.30	1.35	0	-
7d						15	89	3	6.52	8.58	1.34	<1	1.4
8a	**2**	50/250/1/1	0.5	2.5	100	1	30	3	3.13	3.39	1.13	0	-
8b						3	78	17	10.58	9.00	1.15	<1	3.2
8c						5	>99	79	29.86	19.76	1.57	10	7.1
8d						15	>99	98	35.28	31.90	1.94	14	6.8
9a	**3**	50/250/1/1	0.5	2.5	100	1	99	22	13.52	11.97	1.57	11	1.7
9b						3	>99	58	23.86	13.78	2.06	21	3.3
9c						5	>99	82	30.71	20.47	1.72	31	4.0
9d						15	>99	96	34.71	26.07	1.77	32	4.6
10a	**4**	50/250/1/1	0.5	2.5	100	1	94	3	7.74	13.07	1.26	0	-
10b						3	>99	43	19.51	21.73	1.65	7	5.7
10c						5	>99	78	29.57	35.40	1.72	11	6.4

^1^ Determined by the analysis of ^1^H NMR spectra of the reaction mixtures. ^2^ Calculated by the formula *M*_n_ (theo) = Σ(*M*(monomer) × Conversion) + 108.14, where 108.14 is *M* of the initiator BnOH. ^3^ Determined by SEC vs. polystyrene standards and corrected by a factor of 0.57 (average value for PCL/PLA). ^4^ Percentage of mono-lactate fragments CLC in PLLA (see Section 3.4). ^5^ ASL_C_—average sequence length of the PCL fragment in the copolymer, calculated by integration of –CH_2_O– signals at 4.05 ppm (CC sequence) and 4.13 ppm (CL sequence, see Section 3.4).

**Table 3:** Copolymerization results for εCL using **3**/BnOH catalysis (0.5 M comonomer and 2.5 M εCL solutions in toluene, [Comon]/[εCL]/[3]/[BnOH] 50:250:1:1, 100 °C).

Run	Comonomer	Reaction Time, h	Conversion, %		*M*_n_ (theo), ×10^3^ Da ^1^	*M*_n_ (SEC), ×10^3^ Da ^2^	*Đ*_M_^2^	ASL_C_^3^
			Comon.	εCL				
1	PLLA	5	-	97	34.99	22.96	1.74	6.0
2		15	-	99	35.56	27.25	1.85	5.9
3a	MeGL	1	94	~1	6.51	5.59	1.37	~1
3b		3	>99	58	23.16	14.25	1.86	3.1
3c		5	>99	91	32.58	23.59	1.79	3.6
4a	PhGL	1	>99	2	10.29	1.54	3.20	-
4b		3	>99	5	11.14	1.49	3.56	-
4c		5	>99	12	13.14	1.51	5.64	-
5a	EtEP	1	>99	>99	35.16	23.02	1.48	20
5b		3	>99	>99	35.16	19.23	1.94	19
5c		5	>99	>99	35.16	24.01	1.60	14.5
6a	EtOEP	1	>99	81	30.83	36.12	1.91	6.8
6b		3	>99	82	31.11	34.55	2.12	5.2
6c		5	>99	84	31.68	38.77	2.42	4.5

^1^ Calculated by the formula *M*_n_ (theo) = *M*(comonomer) × Conversion + *M*(εCL) × Conversion + 108.14, where 108.14 is *M* of the initiator BnOH. ^2^ Determined by SEC vs. polystyrene standards and corrected by a factor of 0.56 (PCL). ^3^ ASL_C_—average sequence length of PCL fragment in copolymer, calculated by integration of –CH_2_O– signals at 4.05 ppm (CC sequence) and signal of C-comonomer fragment.

**Table 4:** The Zn-L bond distances.

Bond	Distance, Å
Zn-O1	1.9265(10)
Zn-C28	1.9733(15)
Zn1-N1	2.0591(13)
Zn1-O2	2.2274(12)

## Supplementary Materials

The following are available online at https://www.mdpi.com/2073-4360/12/10/2273/s1, Figure S1: ^1^H NMR spectrum of the reaction mixture of εCL polymerization, catalyzed by **1**/BnOH (Table 1, Entry 7, εCL/**1**/BnOH ratio 100:1:1), Figure S2: ^1^H NMR spectrum of the reaction mixture of εCL polymerization, catalyzed by **3**/BnOH (Table 1, Entry 12, εCL/**3**/BnOH ratio 100:1:1), Figure S3: DSC curves (second heat) of PLLA obtained in the presence of **1**/BnOH (A) and **3**/BnOH (B) (Table 1, Entries 4 and 6, respectively), Figure S4: DSC curve (second heat) of *l*LA/εCL copolymer obtained in the presence of **3**/BnOH at 1:1 *l*LA/εCL ratio (Table 2, Entry 6), Figure S5: DSC curves (second heat) of *l*LA/εCL copolymers obtained in the presence of **3**/BnOH at 1:5 *l*LA/εCL ratio after 5 h (top) and 15 h (bottom) (Table 2, Entries 9c and 9d, respectively), Figure S6: Molecular structure of the complex **4**. The probability for thermal ellipsoids is set to the 50% level, Figure S7: ^1^H NMR spectrum (CDCl_3_, 600 MHz) of *l*LA/εCL copolymer obtained by transesterification of PLLA (Table 4, Entry 2, reprecipitated from MeOH), Figure S8: ^13^C NMR spectrum (CDCl_3_, 151 MHz) of *l*LA/εCL copolymer obtained by transesterification of PLLA (Table 4, Entry 2, reprecipitated from MeOH), Figure S9: DSC curves (second heat) of *l*LA/εCL copolymers obtained in the presence of **3**/BnOH by transesterification of PLLA after 5 h (top) and 15 h (bottom) (Table 4, Entries 1 and 2, respectively), Figure S10: ^1^H NMR spectrum (CDCl_3_, 600 MHz) of MeGL/εCL copolymer (Table 4, Entry 3c, reprecipitated from MeOH), Figure S11: ^13^C NMR spectrum (CDCl_3_, 151 MHz) of MeGL/εCL copolymer (Table 4, Entry 3c, reprecipitated from MeOH), Figure S12: ^1^H NMR spectrum (CDCl_3_, 600 MHz) of EtEP/εCL copolymer (Table 4, Entry 5c), Figure S13: ^13^C NMR spectrum (CDCl_3_, 151 MHz) of EtEP/εCL copolymer (Table 4, Entry 5c), Figure S14: ^31^P NMR spectra (CDCl_3_, 162 MHz) of EtEP/εCL copolymerization probes after 1, 3 and 5 h (Table 4, Entries 5a–c), Figure S15: ^1^H NMR spectrum (CDCl_3_, 400 MHz) of EtOEP/εCL copolymer (Table 4, Entry 6c, reprecipitated from MeOH), Figure S16: ^13^C NMR spectrum (CDCl_3_, 101 MHz) of EtOEP/εCL copolymer (Table 4, Entry 6c, reprecipitated from MeOH), Figure S17: ^31^P NMR spectrum (CDCl_3_, 162 MHz) of EtOEP/εCL copolymer (Table 4, Entry 6c, reprecipitated from MeOH), Table S1: Crystal data and structure refinement for **4**, Table S2: Bond lengths, Å; DFT calculations data: plots of the molecular geometries, energies and Cartesian coordinates for stationary points and transition states mentioned in the article.
