# Supplementary material for: Comparative Experimental and Theoretical Study of Mg, Al and Zn Aryloxy Complexes in Copolymerization of Cyclic Esters: The Role of the Metal Coordination in Formation of Random Copolymers
Source: Polymers (Basel). 2020 Oct 2;12(10):2273. doi: 10.3390/polym12102273 (PMC7600584; doi:10.3390/polym12102273)
Supplement: Supplementary file 1 [file polymers-12-02273-s001.zip › LA-CL_SM_rev.pdf]

Comparative experimental and theoretical study of Mg, Al and Zn aryloxy complexes in copolymerization of cyclic esters: the role of the metal coordination in formation of random copolymers

Ilya Nifant'ev [1,2,3,]*, Pavel Komarov [2], Valeriya Ovchinnikova [2], Artem Kiselev [2,3], Mikhail Minyaev [2,4] and Pavel Ivchenko [1,2,]*

[1] Department of Chemistry, M. V. Lomonosov Moscow State University, Leninskie Gory 1–3, Moscow 119991, Russian Federation; inif@org.chem.msu.ru (I.N.); inpv@org.chem.msu.ru (P.I.)
[2] A.V. Topchiev Institute of Petrochemical Synthesis RAS, Leninsky avenue 29, Moscow 119991, Russian Federation; komarrikov@yandex.ru (P.K.); valeriya.160001@gmail.com (V.O.); phpasha1@yandex.ru (P.I.)
[3] Faculty of Chemistry, National Research University Higher School of Economics, Miasnitskaya Str. 20, Moscow 101000, Russian Federation; metra77@mail.ru (A.K.)
[4] N.D. Zelinsky Institute of Organic Chemistry RAS, Leninsky pr. 47, 119991, Moscow, Russian Federation; mminyaev@mail.ru (M.M.)
* Correspondence: ilnif@yahoo.com (I.N.), phpasha1@yandex.ru (P.I.); Tel.: +7-495-939-4098

Supplementary Materials

S1. Homopolymerization of ʟLA and εCL S2–S3

S2. Copolymerization of ʟLA and εCL S3

S3. Crystal and molecular structure of the complex **4** S4–S7

S4. DFT modeling data S8–S70

S5. Synthesis of random εCL copolymers using PLLA and other comonomers S71–S76

S1. Homopolymerization of ʟLA and εCL

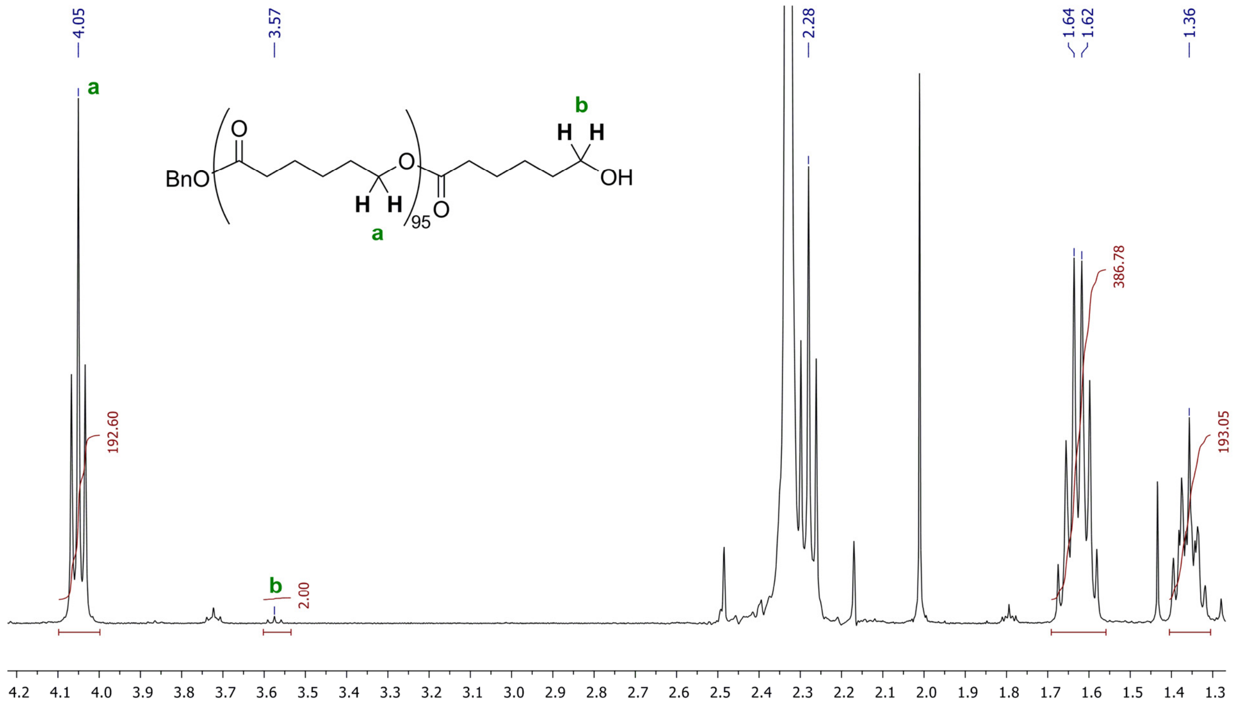

Figure S1. ^{1}H NMR spectrum of the reaction mixture of εCL polymerization, catalyzed by **1**/BnOH (Table 1, Entry 7, εCL/**1**/BnOH ratio 100:1:1).

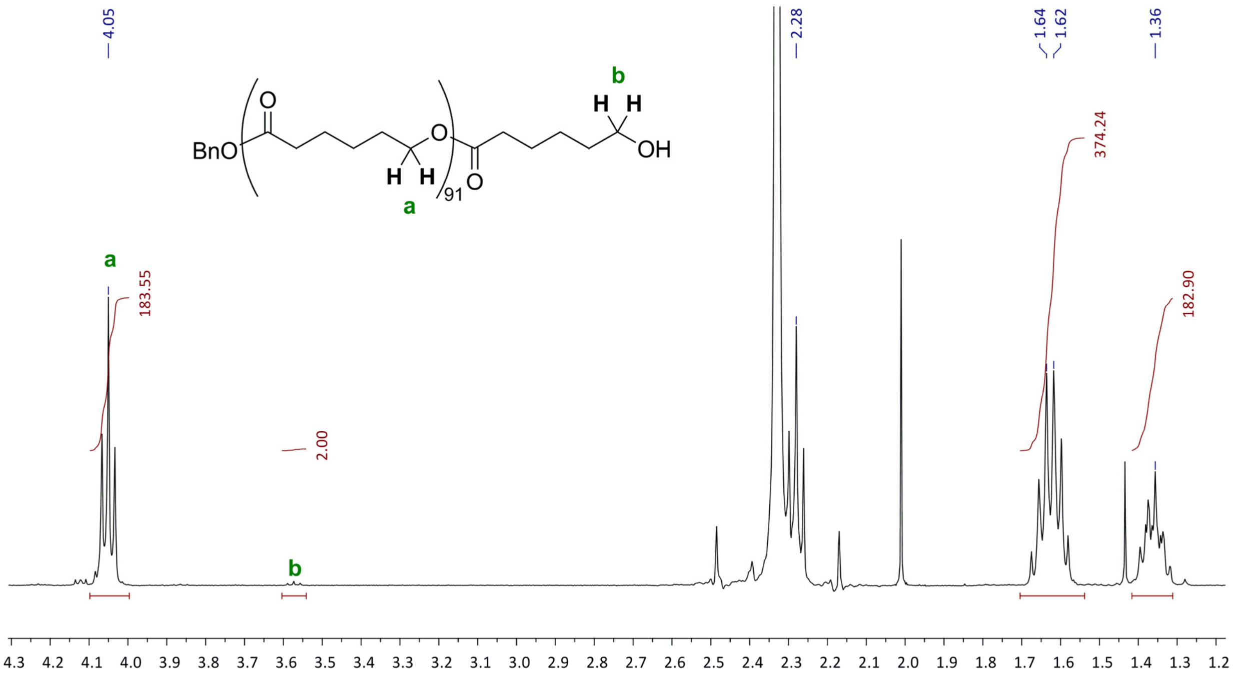

Figure S2. ^{1}H NMR spectrum of the reaction mixture of εCL polymerization, catalyzed by **3**/BnOH (Table 1, Entry 12, εCL/**3**/BnOH ratio 100:1:1).

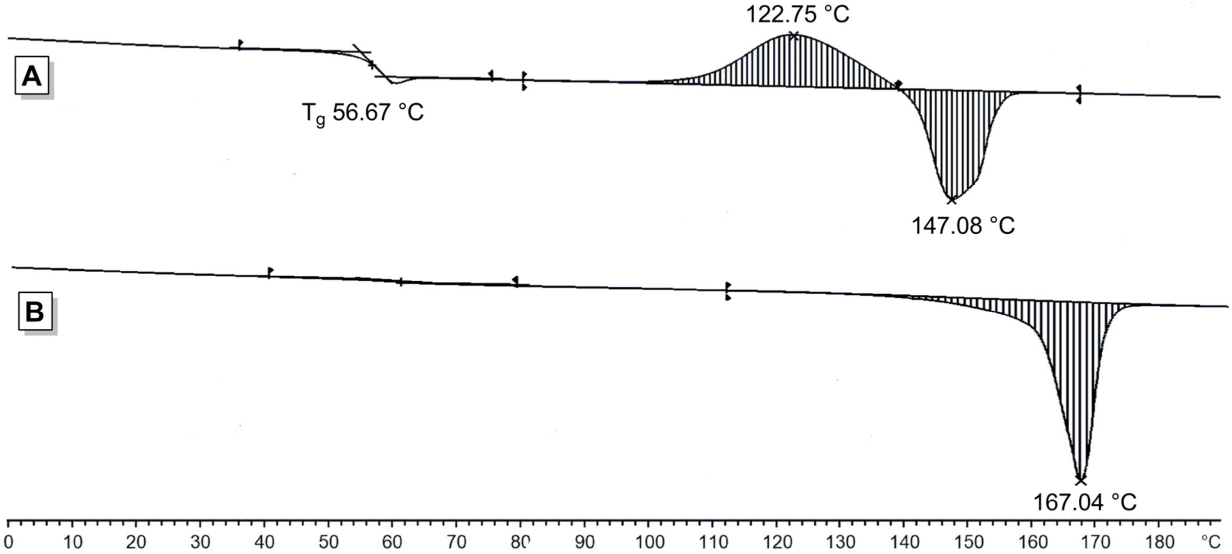

Figure S3. DSC curves (second heat) of PLLA obtained in the presence of **1**/BnOH (A) and **3**/BnOH (B) (Table 1, Entries 4 and 6, respectively).

S2. Copolymerization of LLA and εCL

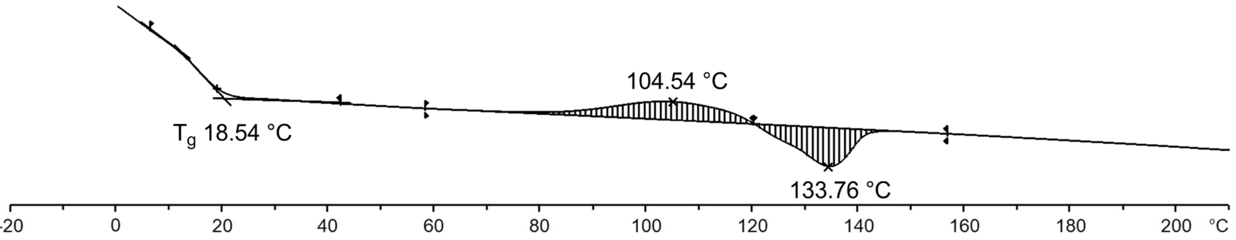

Figure S4. DSC curve (second heat) of *L*LA/εCL copolymer obtained in the presence of **3**/BnOH at 1:1 *L*LA/εCL ratio (Table 2, Entry 6).

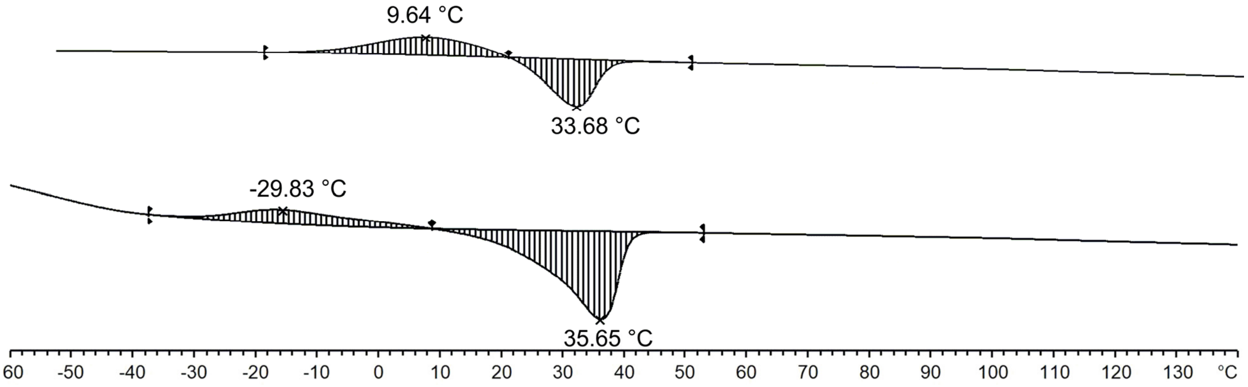

Figure S5. DSC curves (second heat) of *L*LA/εCL copolymers obtained in the presence of **3**/BnOH at 1:5 *L*LA/εCL ratio after 5 h (top) and 15 h (bottom) (Table 2, Entries 9c and 9d, respectively).

S3. Crystal and molecular structure of the complex 4

X-ray crystallographic data and refinement details.

X-ray diffraction data were collected at 100K on a Bruker Quest D8 diffractometer equipped with a Photon-III area-detector (graphite monochromator, shutterless φ- and ω-scan technique), using Mo K_{α}-radiation. The intensity data were integrated by the SAINT program [106] and corrected for absorption and decay using SADABS [107] (multi-scan method, semi-empirical from equivalents). The structure was solved by direct methods using SHELXT [108] and refined by full-matrix least-square method on F^2 using SHELXL-2018 [109]. All non-hydrogen atoms were refined with anisotropic displacement parameters. Hydrogen atoms were found from the electron density-difference map but placed geometrically in ideal calculated positions (C-H distance = 0.950 Å for aromatic, 0.980 Å for methyl, 0.990 Å for methylene and 1.000 Å for tertiary hydrogen atoms) and refined as riding atoms with relative isotropic displacement parameters taken as $U_{iso}(H)=1.5U_{eq}(C)$ for methyl groups and $U_{iso}(H)=1.2U_{eq}(C)$ otherwise. A rotating group model was applied for methyl groups. The SHELXTL program suite [S1] was used for molecular graphics below. Crystal data, data collection and structure refinement details for **4** are summarized in Table S1.

S1. Sheldrick, G.M. A short history of SHELX. *Acta Cryst. A*, **2008**, ***A64***, 112–122. DOI: 10.1107/S0108767307043930.

Table S1. Crystal data and structure refinement for **4**.

Empirical formula	$C_{33}H_{51}NO_2Zn$
Formula weight	559.11
Temperature, K	100(2)
Wavelength, Å	0.71073
Crystal system	Monoclinic
Space group	$P2_1/n$
Unit cell dimensions	
a, Å	9.1404(3)
b, Å	21.7341(7)
c, Å	15.5897(5)
β, °	98.2625(18)
Volume, $Å^3$	3064.88(17)
Z	4
Density (calculated), g/cm^3	1.212
Absorption coefficient, mm^{-1}	0.830
F(000)	1208
Crystal size, mm	0.12 x 0.09 x 0.07
θ range for data collection, °	2.292 to 34.342
Index ranges	-14<=h<=14,
	-34<=k<=33,
	-24<=l<=24
Reflections collected	97780
Independent reflections [R(int)]	12860 [0.0713]
Observed reflections	9852
Completeness to $θ_{max}$, %	100.0
Max. and min. transmission	0.5668 and 0.3880
Data / restraints / parameters	12860 / 0 / 345
Goodness-of-fit on F^2	1.076
Final R1, wR2 indices [I>2σ(I)]	0.0468, 0.1066
Final R1, wR2 indices (all data)	0.0699, 0.1228
Largest diff. peak and hole, e•$Å^3$	0.846, -1.064
CCDC	2021607

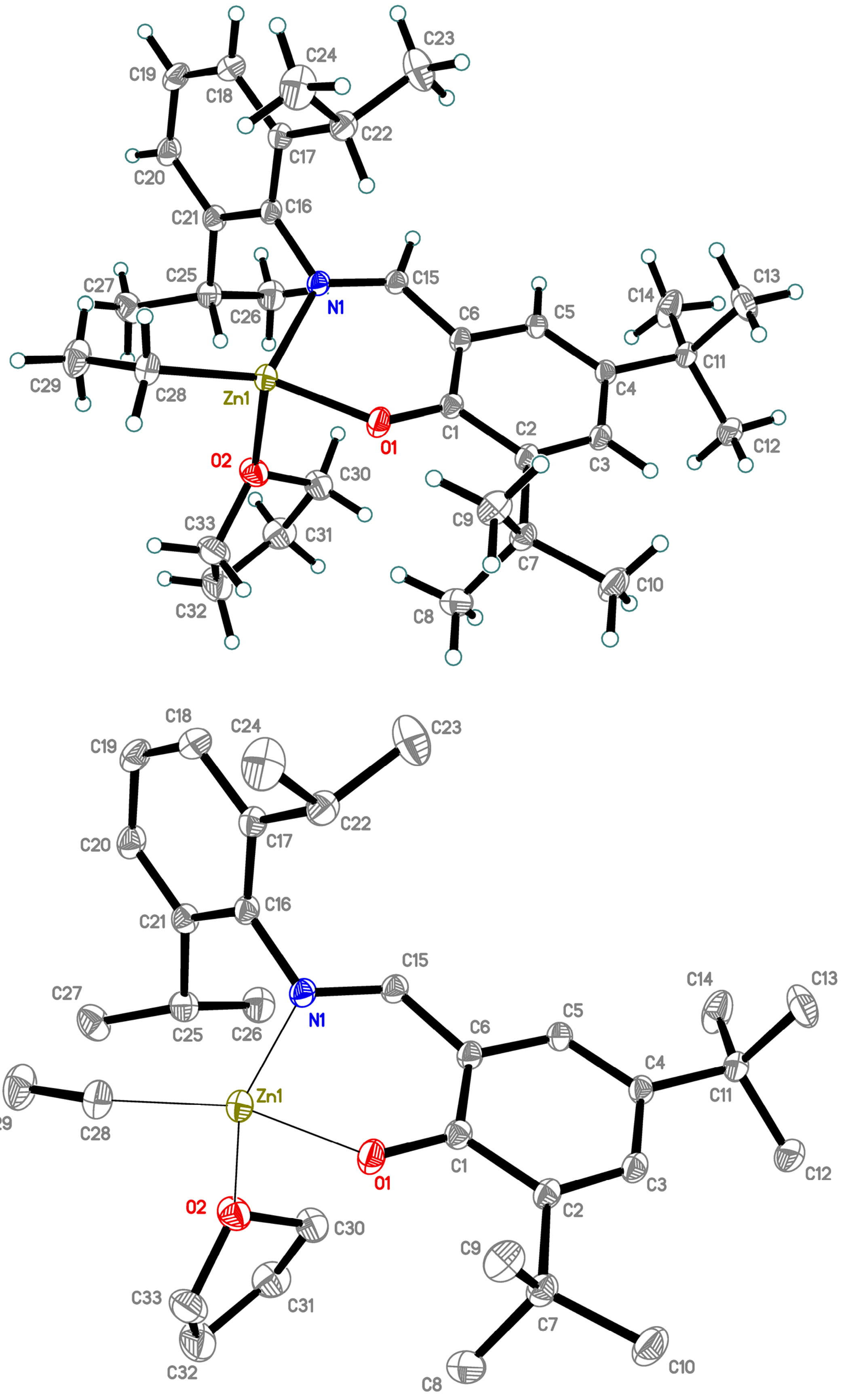

Figure S6. Molecular structure of the complex **4**. The probability for thermal ellipsoids is set to the 50% level.

Table S2. Bond lengths, Å.

Bond	Distance	Bond	Distance	Bond	Distance
Zn(1)-O(1)	1.9265(10)	C(7)-C(10)	1.535(2)	C(20)-C(21)	1.396(2)
Zn(1)-C(28)	1.9733(15)	C(7)-C(9)	1.539(2)	C(21)-C(25)	1.516(2)
Zn(1)-N(1)	2.0591(13)	C(7)-C(8)	1.541(2)	C(22)-C(23)	1.521(3)
Zn(1)-O(2)	2.2274(12)	C(11)-C(14)	1.528(2)	C(22)-C(24)	1.529(2)
O(1)-C(1)	1.2999(17)	C(11)-C(12)	1.532(2)	C(25)-C(27)	1.532(2)
C(1)-C(6)	1.427(2)	C(11)-C(13)	1.533(2)	C(25)-C(26)	1.535(2)
C(1)-C(2)	1.4422(19)	C(15)-N(1)	1.2983(19)	C(28)-C(29)	1.533(2)
C(2)-C(3)	1.382(2)	N(1)-C(16)	1.4379(18)	O(2)-C(33)	1.436(2)
C(2)-C(7)	1.534(2)	C(16)-C(17)	1.404(2)	O(2)-C(30)	1.442(2)
C(3)-C(4)	1.416(2)	C(16)-C(21)	1.410(2)	C(30)-C(31)	1.518(2)
C(4)-C(5)	1.3745(19)	C(17)-C(18)	1.399(2)	C(31)-C(32)	1.539(3)
C(4)-C(11)	1.532(2)	C(17)-C(22)	1.523(2)	C(32)-C(33)	1.529(3)
C(5)-C(6)	1.417(2)	C(18)-C(19)	1.385(2)		
C(6)-C(15)	1.4387(19)	C(19)-C(20)	1.391(2)		

Deviation of Zn1 from the plane defined by atoms O1, N1 and C28 is 0.3096(9)Å. Deviations from the O1, C1..C6, C15, N1 plane are 0.0522(23)A for C28 and 0.2957(11)A for Zn1.

S4. DFT modeling data

Data for structures presented in Scheme 5

εCL

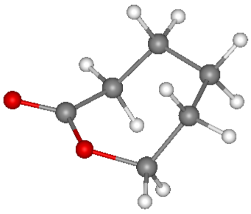

Zero-point vibrational energy	413924.6 (Joules/Mol)
	98.93035 (Kcal/Mol)
Zero-point correction=	0.157656 (Hartree/Particle)
Thermal correction to Energy=	0.164853
Thermal correction to Enthalpy=	0.165798
Thermal correction to Gibbs Free Energy=	0.126149
Sum of electronic and zero-point Energies=	-384.860246
Sum of electronic and thermal Energies=	-384.853048
Sum of electronic and thermal Enthalpies=	-384.852104
Sum of electronic and thermal Free Energies=	-384.891753

cartesian

8	-2.49640012	0.09650000	-0.41440001	1	0.34410000	1.60000002	-1.28910005
6	-1.36909997	-0.02400000	0.01080000	1	-0.27320001	0.89160001	1.62860000
8	-0.77679998	-1.24290001	-0.07160000	1	-1.30460000	1.95330000	0.67290002
6	-0.58899999	1.13250005	0.60570002	6	1.86199999	0.67570001	-0.04630000
6	0.55019999	-1.48150003	0.42449999	1	0.60909998	-1.21550000	1.48740005
6	0.62889999	1.55929995	-0.23090000	1	1.42149997	-0.92960000	-1.44739997
6	1.64820004	-0.79979998	-0.38229999	1	2.58159995	-1.34459996	-0.19520000
1	0.65160000	-2.56620002	0.35339999	1	2.20040011	0.75480002	0.99610001
1	0.88940001	2.58470011	0.05370000	1	2.67910004	1.06920004	-0.66170001

***L*LA**

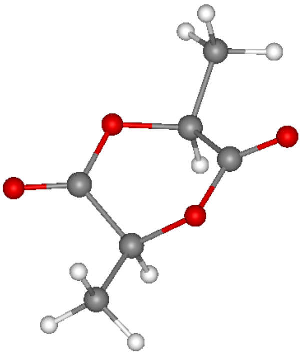

Zero-point vibrational energy	370616.7 (Joules/Mol)
	88.57951 (Kcal/Mol)
Zero-point correction=	0.141160 (Hartree/Particle)
Thermal correction to Energy=	0.150579

Thermal correction to Enthalpy= 0.151523
Thermal correction to Gibbs Free Energy= 0.106579
Sum of electronic and zero-point Energies= -534.087278
Sum of electronic and thermal Energies= -534.077859
Sum of electronic and thermal Enthalpies= -534.076915
Sum of electronic and thermal Free Energies= -534.121860

cartesian

8	-0.25319999	-1.35280001	-0.11950000	1	-3.39499998	-0.39100000	0.48490000
6	-1.25360000	-0.46730000	0.42940000	1	-2.73480010	-1.02769995	-1.02980006
6	1.03890002	-0.96079999	-0.06110000	1	-2.71370006	-2.03559995	0.43599999
6	-1.03890002	0.96079999	-0.06120000	1	1.14250004	0.46200001	1.52349997
6	-2.61220002	-1.01740003	0.05490000	1	2.71329999	2.03600001	0.43540001
1	-1.14269996	-0.46180001	1.52359998	1	3.39499998	0.39160001	0.48590001
6	1.25360000	0.46730000	0.42940000	1	2.73539996	1.02680004	-1.02960002
6	2.61220002	1.01740003	0.05500000	8	1.92960000	-1.71019995	-0.37459999
8	0.25319999	1.35270000	-0.11990000	8	-1.92960000	1.71029997	-0.37439999

MeOCOCHMeOMe

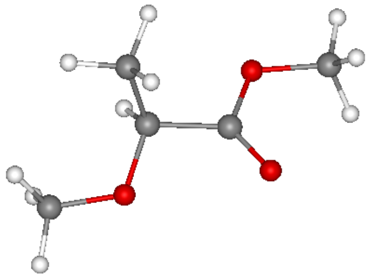

Zero-point vibrational energy 399062.4 (Joules/Mol)
95.37821 (Kcal/Mol)
Zero-point correction= 0.151995 (Hartree/Particle)
Thermal correction to Energy= 0.161841
Thermal correction to Enthalpy= 0.162785
Thermal correction to Gibbs Free Energy= 0.116332
Sum of electronic and zero-point Energies= -421.955227
Sum of electronic and thermal Energies= -421.945381
Sum of electronic and thermal Enthalpies= -421.944437
Sum of electronic and thermal Free Energies= -421.990890

cartesian

8	1.81224990	0.14504446	0.49837777	6	-2.72735000	-0.71005553	0.40547779
6	3.16174984	-0.26985553	0.23547778	1	-3.23594999	0.18624447	0.02507779
1	3.40944982	-0.12455554	-0.81832218	1	-2.63935018	-0.62855554	1.49917781
1	3.29414988	-1.32305551	0.49197778	1	-3.33665013	-1.58315551	0.16517779
8	-1.46875012	-0.91875553	-0.20252222	1	0.08144993	2.08634424	-0.43282220
6	0.86184990	-0.51795554	-0.20052221	1	-1.66675007	1.82704437	-0.43332222
6	-0.51145005	0.06714447	0.11327779	1	-0.68075001	1.15984452	-1.74952221

8	1.09494996	-1.41335547	-0.97732222	1	-0.53295004	0.29144448	1.19077778
6	-0.70515013	1.36724448	-0.67642224	1	3.78924990	0.35894448	0.86597776

MOMP

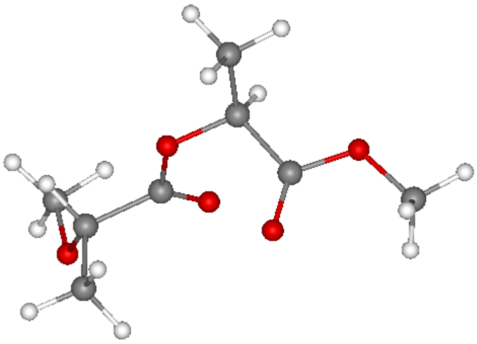

Zero-point vibrational energy	587574.5 (Joules/Mol)
	140.43367 (Kcal/Mol)
Zero-point correction=	0.223795 (Hartree/Particle)
Thermal correction to Energy=	0.239138
Thermal correction to Enthalpy=	0.240082
Thermal correction to Gibbs Free Energy=	0.179949
Sum of electronic and zero-point Energies=	-689.010214
Sum of electronic and thermal Energies=	-688.994872
Sum of electronic and thermal Enthalpies=	-688.993928
Sum of electronic and thermal Free Energies=	-689.054061

cartesian

6	3.81060004	0.40250000	-1.19819999	1	1.82110000	-2.10730004	1.20060003
8	3.34100008	-0.56029999	-0.27059999	8	-3.02309990	-0.14229999	-0.71429998
6	2.30380011	-0.10370000	0.55860001	8	-1.69560003	-1.07609999	0.86369997
6	0.99890000	0.10800000	-0.21230000	6	-3.88910007	-1.28760004	-0.63459998
8	0.16970000	0.92170000	0.49000001	1	-4.69390011	-1.09389997	-1.34210002
6	-1.13680005	1.10640001	-0.06220000	1	-3.34389997	-2.19199991	-0.91100001
6	2.08260012	-1.14690006	1.65050006	1	-4.28450012	-1.39800000	0.37720001
1	2.57060003	0.86059999	1.02040005	1	4.66039991	-0.04890000	-1.71329999
8	0.71630001	-0.37670001	-1.28390002	1	3.04489994	0.66289997	-1.93859994
1	-1.05540001	1.32710004	-1.12969995	1	4.14909983	1.31579995	-0.68640000
6	-1.95070004	-0.17659999	0.09590000	1	-2.78390002	2.45260000	0.28740001
6	-1.78470004	2.26340008	0.68540001	1	-1.86249995	2.03880000	1.75209999
1	1.27779996	-0.84230000	2.32189989	1	-1.18139994	3.16599989	0.56150001
1	3.00620008	-1.26610005	2.22199988				

I-0_Mg

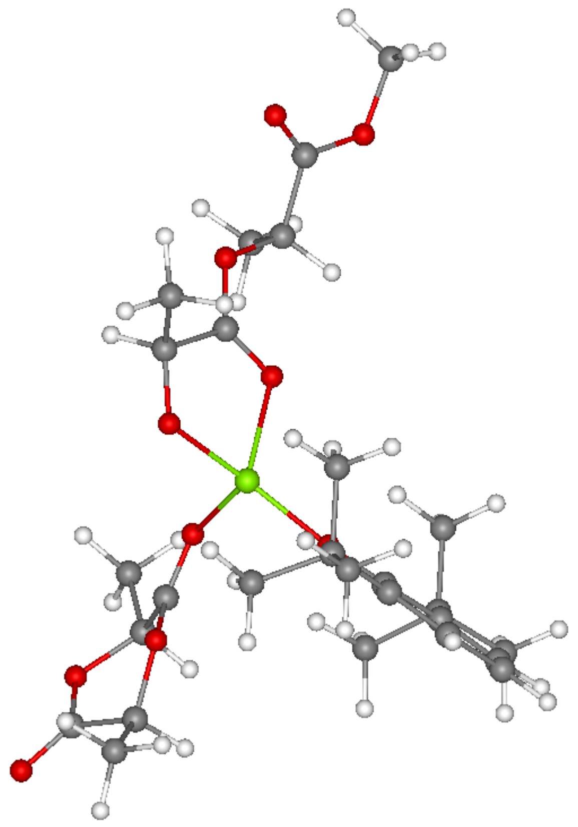

Zero-point vibrational energy	1699528.2 (Joules/Mol)
	406.19700 (Kcal/Mol)
Zero-point correction=	0.647316 (Hartree/Particle)
Thermal correction to Energy=	0.691171
Thermal correction to Enthalpy=	0.692115
Thermal correction to Gibbs Free Energy=	0.562495
Sum of electronic and zero-point Energies=	-2004.261220
Sum of electronic and thermal Energies=	-2004.217365
Sum of electronic and thermal Enthalpies=	-2004.216421
Sum of electronic and thermal Free Energies=	-2004.346041

cartesian

8	1.93717539	1.41153085	-0.79625374	6	2.64087486	-3.43496919	1.53594601
6	3.54897451	2.96373105	-1.68945408	6	3.57667446	-0.79426944	-3.26125383
6	3.07737446	1.85213077	-0.77305377	1	3.26227450	-0.37276965	-2.30605412
8	3.97127438	1.35553098	0.06494641	1	3.32017446	-0.09466958	-4.06825352
6	5.36168003	1.76263034	-0.08865345	1	4.66607666	-0.91736943	-3.25025392
6	5.45557976	3.27473092	-0.26275352	6	1.36537564	-1.96936905	-3.63445377
8	4.46137619	3.83372855	-0.99405390	1	0.91567570	-1.56746924	-2.72645378
8	6.33758020	3.94922757	0.20014632	1	0.88587570	-2.92986894	-3.85215378
1	4.08517551	2.47813106	-2.51805377	1	1.14397550	-1.28776932	-4.46595287
6	2.41767502	3.80952859	-2.23375392	6	3.40067458	-2.69856906	-4.82985258
6	6.12237978	1.25393069	1.11384594	1	2.94167471	-3.65856910	-5.08785248
1	5.73538017	1.28763068	-1.00495386	1	4.48887587	-2.82236886	-4.84395218

1	7.17668009	1.51293087	1.00874591	1	3.14297462	-1.99046910	-5.62595224
1	6.02118015	0.16953048	1.18004596	6	1.09887552	-3.40656924	1.59284616
1	5.74298000	1.70953071	2.03024626	1	0.69977564	-4.40117073	1.36634612
1	2.82767487	4.58112717	-2.88795376	1	0.66337568	-2.72106886	0.86334592
1	1.86877537	4.28692722	-1.41995382	1	0.74797571	-3.11256909	2.58794618
1	1.72967541	3.18423104	-2.80405378	6	3.25607467	-2.12856889	2.08024621
12	1.11257553	-0.32806972	0.08304644	1	4.33827543	-2.25096869	2.20314622
8	0.35197553	0.29973048	1.70954609	1	2.82857490	-1.87046909	3.05514622
6	-0.99142480	0.56373084	1.70374632	1	3.10827446	-1.28136933	1.40904605
6	-1.57222521	0.29483050	0.31374633	6	3.07957458	-4.54327154	2.51044631
8	-2.86262417	0.55623078	0.16334635	1	4.16917515	-4.63997221	2.56444621
6	-3.46182203	0.31293049	-1.12335384	1	2.65937495	-5.51917315	2.24604630
6	-1.77222550	-0.24056971	2.75824618	1	2.72357488	-4.29737091	3.51734686
8	-0.86742455	-0.13186958	-0.61355364	1	4.78037691	-6.07527304	-1.70935404
6	-3.46762156	1.59583092	-1.94595408	1	-1.20982492	1.63643062	1.88644636
6	-4.86791992	-0.21196967	-0.85315377	1	-2.83822441	0.00363044	2.76384616
1	-2.89482450	-0.46386969	-1.64175391	1	-1.34392500	-0.00846960	3.73614693
8	2.14617538	-1.65106905	-0.73265374	1	-1.65022516	-1.31376946	2.58324623
6	2.79887486	-2.78566885	-0.97025388	8	-5.42732000	-0.59696954	-2.01585412
6	3.20707464	-3.09686923	-2.30695415	8	-5.40802002	-0.27676964	0.22534633
6	3.91387439	-4.28117085	-2.53435373	6	-6.76652145	-1.11336935	-1.91205406
1	4.23307514	-4.53527164	-3.53965378	1	-7.06042242	-1.36416936	-2.92985392
6	4.22757530	-5.16167307	-1.50455391	1	-7.43412447	-0.35816967	-1.49285400
6	3.81657457	-4.86107302	-0.21075350	1	-6.78162146	-2.00156903	-1.27765393
1	4.06017494	-5.56377316	0.57934588	1	-3.95212030	1.41923082	-2.90835381
6	3.10297465	-3.69726920	0.09144646	1	-2.44022560	1.91933072	-2.12815404
6	2.88867474	-2.15636873	-3.48415375	1	-4.00201988	2.38963103	-1.41785383

I-0_Zn

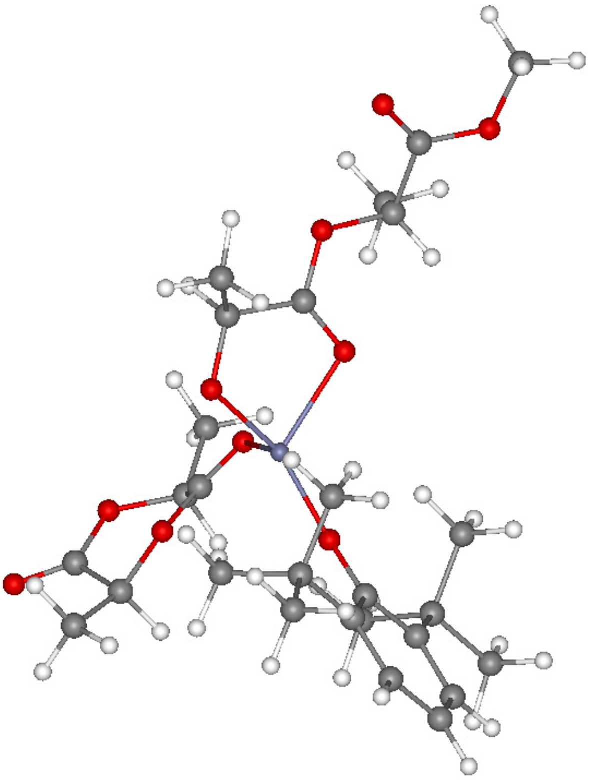

Zero-point vibrational energy	1698085.9 (Joules/Mol)
	405.85226 (Kcal/Mol)
Zero-point correction=	0.646767 (Hartree/Particle)
Thermal correction to Energy=	0.690963
Thermal correction to Enthalpy=	0.691907
Thermal correction to Gibbs Free Energy=	0.561499
Sum of electronic and zero-point Energies=	-3583.223637
Sum of electronic and thermal Energies=	-3583.179440
Sum of electronic and thermal Enthalpies=	-3583.178496
Sum of electronic and thermal Free Energies=	-3583.308905

cartesian

8	0.21799113	2.12526679	-0.30192560	6	1.31329107	-2.46203327	2.25217438
6	1.79439116	3.76546669	-1.10462558	6	2.71769118	0.02906677	-2.52152562
6	1.29929113	2.67436671	-0.17322557	1	2.30039120	0.49566677	-1.62792552
8	2.12269115	2.32436681	0.80927444	1	2.55089116	0.68656677	-3.38522553
6	3.47759128	2.85106659	0.79117441	1	3.79969120	-0.08403322	-2.38612556
6	3.46009111	4.35166693	0.52057445	6	0.55379111	-1.16613317	-3.05712557
8	2.52579117	4.76496649	-0.36842561	1	0.02909113	-0.70553321	-2.22002554
8	4.20939112	5.13746691	1.04027438	1	0.08859113	-2.13663340	-3.26162553
1	2.47859120	3.28246665	-1.81712556	1	0.41659111	-0.53533322	-3.94472551
6	0.67919111	4.46206665	-1.85412550	6	2.69579124	-1.93413329	-4.02702570
6	4.12599087	2.49576664	2.10947442	1	2.26269126	-2.90373325	-4.29482555
1	4.00659084	2.35986662	-0.03592558	1	3.78009129	-2.05563331	-3.93082571
1	5.15529108	2.85706663	2.11477447	1	2.51789117	-1.25523329	-4.86872530

1	4.12279081	1.41256666	2.24027443	6	-0.22890887	-2.51903319	2.15407443
1	3.59049129	2.96126676	2.93897438	1	-0.55050886	-3.56093335	2.05117440
1	1.10539114	5.22316694	-2.51032543	1	-0.63070887	-2.00743318	1.27327442
1	-0.01220887	4.93986654	-1.15732551	1	-0.70420891	-2.09373331	3.04367447
1	0.12859112	3.73716664	-2.45502543	6	1.82789111	-1.11293316	2.80257440
30	-0.19570887	0.19866677	0.83147442	1	2.88459134	-1.21143317	3.07597446
8	-1.02800882	0.88676679	2.36597443	1	1.26939106	-0.81323320	3.69537449
6	-2.36550879	1.19816685	2.23577452	1	1.77709115	-0.29553324	2.08027434
6	-2.91970873	0.74676681	0.88617438	6	1.69789112	-3.50723338	3.31637454
8	-4.20330906	1.02536678	0.69357443	1	2.78049111	-3.55623341	3.47347450
6	-4.79080915	0.64296675	-0.56422561	1	1.33909106	-4.51063347	3.06607437
6	-3.20400882	0.60446680	3.37807441	1	1.24069107	-3.22853327	4.27267456
8	-2.22560883	0.18346676	0.03447443	1	3.77059126	-5.19403315	-0.67022562
6	-4.64290905	1.76936674	-1.58052552	1	-2.52830887	2.29416680	2.23907447
6	-6.25080919	0.31436676	-0.27332556	1	-4.25640917	0.89176679	3.30907440
1	-4.29630899	-0.25983322	-0.93092555	1	-2.79310870	0.97046679	4.32177448
8	1.07559109	-0.72443324	-0.16372557	1	-3.12860870	-0.48683321	3.37687445
6	1.72289109	-1.89493322	-0.24722557	8	-6.81120920	-0.22083324	-1.37442553
6	2.26139116	-2.24693322	-1.52562559	8	-6.82880878	0.50346678	0.77057439
6	2.98809123	-3.43403339	-1.64032555	6	-8.20230865	-0.56693321	-1.25002551
1	3.40659118	-3.71953321	-2.59942555	1	-8.48730850	-0.97513324	-2.21832561
6	3.19789124	-4.27733326	-0.55392557	1	-8.79550838	0.31916678	-1.01592553
6	2.66039133	-3.93433332	0.68017441	1	-8.33870888	-1.31163335	-0.46362561
1	2.82619119	-4.60473347	1.51647449	1	-5.11450911	1.48386669	-2.52312565
6	1.91899121	-2.76263332	0.87097442	1	-3.58340883	1.95876670	-1.76542556
6	2.05639124	-1.34483314	-2.75782561	1	-5.11130905	2.68506670	-1.21052551

I-0'_Mg

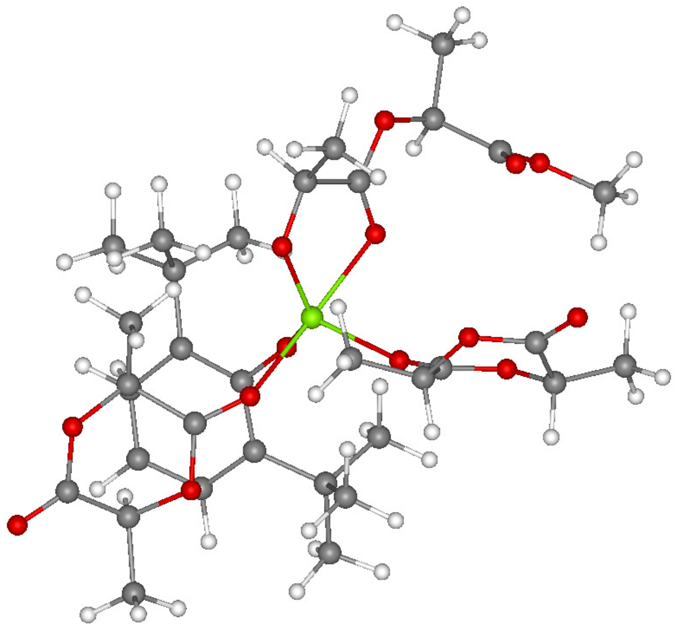

Zero-point vibrational energy	2074414.0 (Joules/Mol)
	495.79685 (Kcal/Mol)
Zero-point correction=	0.790103 (Hartree/Particle)
Thermal correction to Energy=	0.844707
Thermal correction to Enthalpy=	0.845651
Thermal correction to Gibbs Free Energy=	0.694664
Sum of electronic and zero-point Energies=	-2538.360821
Sum of electronic and thermal Energies=	-2538.306217
Sum of electronic and thermal Enthalpies=	-2538.305273
Sum of electronic and thermal Free Energies=	-2538.456260

cartesian

8	-0.97570002	1.06260002	0.75880003	6	2.73230004	0.49540001	-3.52099991
6	-2.22029996	1.29859996	1.13259995	8	5.01840019	1.31789994	-0.09780000
6	-2.78020000	0.63639998	2.27900004	1	5.24609995	1.12500000	2.57270002
6	-4.15679979	0.74860001	2.50309992	1	5.83839989	2.66020012	3.28160000
1	-4.61049986	0.22700000	3.33999991	1	3.80550003	5.20660019	-1.50800002
6	-4.97889996	1.53610003	1.69879997	1	4.89270020	5.08580017	-0.11000000
6	-4.39169979	2.32259989	0.71039999	1	5.28310013	4.22840023	-1.61969995
1	-5.02510023	3.01449990	0.16460000	1	3.45339990	1.24880004	-3.85050011
6	-3.02060008	2.26909995	0.43450001	1	3.27010012	-0.28680000	-2.97690010
6	-1.89300001	-0.10640000	3.30010009	1	2.26410007	0.04370000	-4.39919996
6	-2.38919997	3.30999994	-0.51560003	1	1.19140005	1.95179999	-3.21609998
6	-1.32070005	-1.41709995	2.73270011	1	2.87369990	3.63949990	0.19949999
1	-0.68980002	-1.23930001	1.86339998	6	-4.75930023	-1.91050005	-0.08390000
1	-0.71170002	-1.92240000	3.49349999	8	-3.32920003	-2.19239998	-0.09110000
1	-2.12100005	-2.10039997	2.43099999	6	-2.57640004	-1.49039996	-0.92780000
6	-0.73799998	0.81590003	3.74440002	6	-3.28609991	-0.80220002	-2.07999992
1	-0.11630000	1.11459994	2.89940000	8	-4.49230003	-1.50119996	-2.45709991

1	-1.13139999	1.72220004	4.21780014	6	-5.31440020	-1.97379994	-1.49720001
1	-0.10630000	0.30039999	4.47860003	8	-1.36769998	-1.43669999	-0.75849998
6	-2.67459989	-0.47889999	4.57310009	1	-4.88149977	-0.88800001	0.29840001
1	-3.12910008	0.39640000	5.04879999	6	-5.41349983	-2.89919996	0.85270000
1	-3.46329999	-1.21560001	4.38299990	6	-2.42210007	-0.70539999	-3.32299995
1	-1.98679996	-0.92790002	5.29829979	1	-3.55990005	0.19800000	-1.71679997
6	-3.37779999	4.43839979	-0.86500001	8	-6.40240002	-2.41470003	-1.77419996
1	-4.22840023	4.08620024	-1.45850003	1	-2.95199990	-0.12410000	-4.08059978
1	-3.76519990	4.93569994	0.03030000	1	-2.23390007	-1.70599997	-3.72149992
1	-2.86129999	5.19479990	-1.46650004	1	-1.46179998	-0.23260000	-3.10080004
6	-1.18690002	3.98040009	0.18279999	1	-6.48290014	-2.69129992	0.90520000
1	-0.42230001	3.25049996	0.45190001	1	-4.98019981	-2.80010009	1.84959996
1	-0.74239999	4.73420000	-0.47929999	1	-5.27799988	-3.92260003	0.49610001
1	-1.51209998	4.48780012	1.09770000	6	2.23449993	-3.45169997	-0.62169999
6	-1.93830001	2.69549990	-1.85200000	8	3.57489991	-3.67260003	-1.11109996
1	-2.79660010	2.30220008	-2.40939999	6	4.56850004	-3.77500010	-0.20119999
1	-1.47019994	3.46059990	-2.48429990	6	4.16979980	-3.42630005	1.23060000
1	-1.21399999	1.88870001	-1.72710001	8	3.24309993	-2.30850005	1.25129998
1	-6.04740000	1.59669995	1.89139998	6	2.24550009	-2.30200005	0.37329999
12	0.16750000	0.07190000	-0.41389999	8	5.67509985	-4.12680006	-0.51889998
8	0.66360003	0.18790001	-2.27979994	1	1.91900003	-4.34889984	-0.06800000
6	4.58549976	2.36849999	0.31810001	6	1.31980002	-3.23140001	-1.80390000
6	3.69099998	3.33260012	-0.45800000	6	5.35340023	-3.05220008	2.09400010
8	3.14759994	2.67770004	-1.61459994	1	3.64899993	-4.29510021	1.65579998
6	2.21869993	1.74539995	-1.39979994	8	1.41340005	-1.41659999	0.41610000
6	1.62030005	1.10049999	-2.64809990	1	5.01389980	-2.82279992	3.10619998
8	4.85480022	2.88400006	1.52950001	1	5.86770010	-2.18420005	1.67799997
6	5.71390009	2.08879995	2.36319995	1	6.05480003	-3.88669991	2.12960005
1	6.67780018	1.92859995	1.87570000	1	0.28310001	-3.22029996	-1.46449995
8	1.83860004	1.44630003	-0.26719999	1	1.45330000	-4.05280018	-2.51169991
6	4.47219992	4.53959990	-0.95660001	1	1.51859999	-2.27810001	-2.29769993

I-0'_Zn

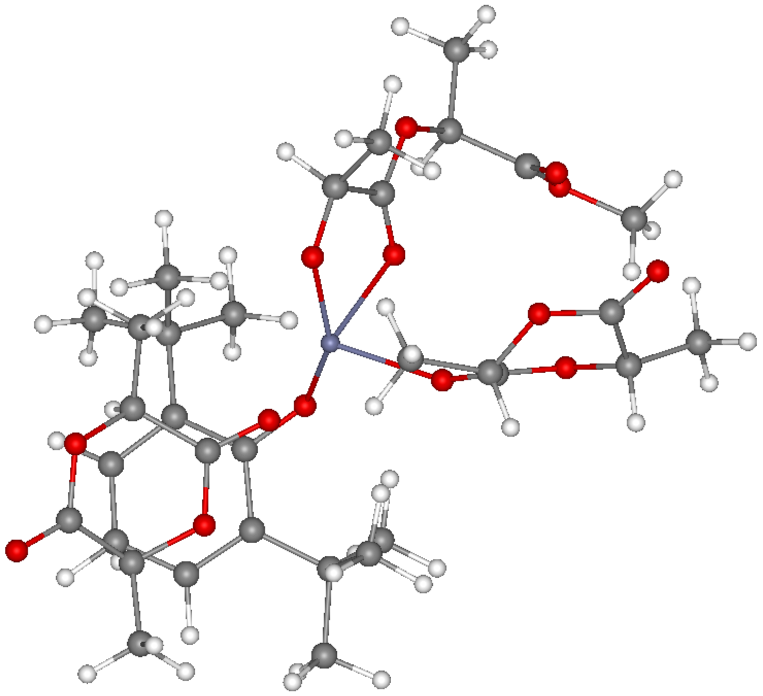

Zero-point vibrational energy	2073160.9 (Joules/Mol)
	495.49736 (Kcal/Mol)
Zero-point correction=	0.789625 (Hartree/Particle)
Thermal correction to Energy=	0.844762
Thermal correction to Enthalpy=	0.845706
Thermal correction to Gibbs Free Energy=	0.692996
Sum of electronic and zero-point Energies=	-4117.315679
Sum of electronic and thermal Energies=	-4117.260542
Sum of electronic and thermal Enthalpies=	-4117.259598
Sum of electronic and thermal Free Energies=	-4117.412308

cartesian

8	-0.82130003	1.08469999	1.06610000	6	5.01130009	4.08799982	-1.25000000
6	-2.12010002	1.32790005	1.26100004	6	2.76880002	0.09520000	-3.34990001
6	-2.83100009	0.58899999	2.26309991	8	5.13600016	0.95980000	-0.03270000
6	-4.21689987	0.75790000	2.35109997	1	5.29740000	1.00320005	2.65030003
1	-4.78359985	0.18960001	3.08159995	1	6.06409979	2.51719999	3.22580004
6	-4.90460014	1.66240001	1.54460001	1	4.44269991	4.77239990	-1.88399994
6	-4.17799997	2.49510002	0.69900000	1	5.51160002	4.66650009	-0.47080001
1	-4.71579981	3.26500010	0.15549999	1	5.76480007	3.58699989	-1.86259997
6	-2.78710008	2.39089990	0.56599998	1	3.55640006	0.73820001	-3.75149989
6	-2.09699988	-0.29170001	3.29469991	1	3.23160005	-0.67570001	-2.72779989
6	-2.02029991	3.47070003	-0.23029999	1	2.25410008	-0.39520001	-4.17980003
6	-1.43889999	-1.52330005	2.64960003	1	1.37989998	1.72150004	-3.19479990
1	-0.67170000	-1.23559999	1.93379998	1	3.31209993	3.56909990	-0.00640000
1	-0.96420002	-2.13899994	3.42470002	6	-5.04440022	-1.77110004	-0.26870000
1	-2.17820001	-2.14129996	2.13059998	8	-3.62470007	-2.08119988	-0.25580001

6	-1.02119994	0.55620003	4.00699997	6	-2.81900001	-1.30799997	-0.98509997
1	-0.28610000	0.94150001	3.29889989	6	-3.47219992	-0.47009999	-2.07209992
1	-1.48010004	1.40509999	4.52619982	8	-4.69070005	-1.07169998	-2.56250000
1	-0.49869999	-0.05260000	4.75530005	6	-5.55159998	-1.64150000	-1.69529998
6	-3.04870009	-0.81370002	4.38579988	8	-1.61909997	-1.31480002	-0.77920002
1	-3.55769992	-0.00300000	4.91800022	1	-5.16620016	-0.80000001	0.22950000
1	-3.80859995	-1.49640000	3.98850012	6	-5.75479984	-2.85319996	0.51239997
1	-2.46849990	-1.37609994	5.12529993	6	-2.56399989	-0.28320000	-3.27279997
6	-2.91269994	4.68620014	-0.54659998	1	-3.72070003	0.49750000	-1.61399996
1	-3.72720003	4.44700003	-1.23880005	8	-6.64120007	-2.01220012	-2.05769992
1	-3.34699988	5.12220001	0.35890001	1	-3.03220010	0.40880001	-3.97609997
1	-2.30509996	5.46049976	-1.02800000	1	-2.41459990	-1.24249995	-3.77500010
6	-0.83800000	4.00409985	0.60570002	1	-1.58749998	0.09920000	-2.96939993
1	-0.12330000	3.21749997	0.84710002	1	-6.82250023	-2.63290000	0.54820001
1	-0.31900001	4.79710007	0.05270000	1	-5.36089993	-2.89000010	1.52980006
1	-1.19970000	4.43489981	1.54579997	1	-5.61959982	-3.82730007	0.03730000
6	-1.51110005	2.94330001	-1.58200002	6	1.99430001	-3.50189996	-0.43220001
1	-2.34360003	2.62829995	-2.22049999	8	3.27010012	-3.78069997	-1.05149996
1	-0.96649998	3.73000002	-2.11870003	6	4.35470009	-3.85840011	-0.24959999
1	-0.83170003	2.09459996	-1.47930002	6	4.11670017	-3.43210006	1.19610000
1	-5.98350000	1.77040005	1.63059998	8	3.22490001	-2.29119992	1.25779998
30	0.11450000	0.22130001	-0.32429999	6	2.13190007	-2.30270004	0.49290001
8	0.67690003	0.09870000	-2.13599992	8	5.41480017	-4.24889994	-0.66729999
6	4.82880020	2.08979988	0.27180001	1	1.72469997	-4.36299992	0.19760001
6	4.07030010	3.07329988	-0.61760002	6	0.96200001	-3.31890011	-1.51950002
8	3.42910004	2.36890006	-1.69180000	6	5.39279985	-3.05250001	1.91320002
6	2.38899994	1.59609997	-1.36679995	1	3.62570000	-4.26800013	1.71379995
6	1.72969997	0.89240003	-2.54809999	8	1.32560003	-1.39929998	0.58420002
8	5.14319992	2.68569994	1.43350005	1	5.16629982	-2.77600002	2.94510007
6	5.88450003	1.87750006	2.36319995	1	5.87449980	-2.21210003	1.41040003
1	6.82889986	1.55480003	1.92009997	1	6.07880020	-3.90059996	1.91009998
8	1.97829998	1.49489999	-0.21480000	1	-0.02940000	-3.23959994	-1.07149994
				1	0.98710001	-4.18739986	-2.18190002
				1	1.14300001	-2.40700006	-2.09159994

I-0''k^2_Mg

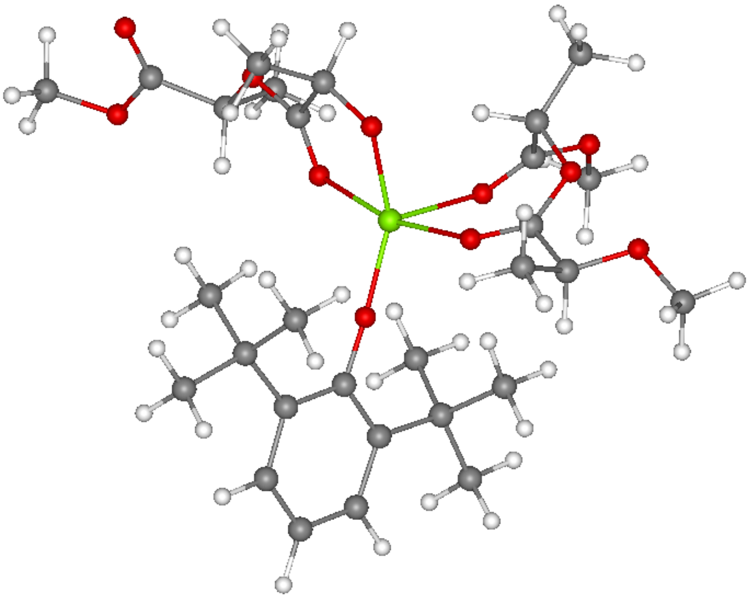

Zero-point vibrational energy	1917623.6 (Joules/Mol)
	458.32304 (Kcal/Mol)
Zero-point correction=	0.730384 (Hartree/Particle)
Thermal correction to Energy=	0.779677
Thermal correction to Enthalpy=	0.780621
Thermal correction to Gibbs Free Energy=	0.640769
Sum of electronic and zero-point Energies=	-2159.186740
Sum of electronic and thermal Energies=	-2159.137447
Sum of electronic and thermal Enthalpies=	-2159.136503
Sum of electronic and thermal Free Energies=	-2159.276355

cartesian

8	0.25483000	1.20981121	-0.11270005	1	-4.26807022	-0.30468869	-0.67980003
6	0.40192997	2.51471138	-0.26680005	6	-6.07207012	-1.20838869	0.04329995
6	0.86322999	3.04261136	-1.51850009	6	-4.63527012	-2.09018874	-1.83590007
6	1.02232993	4.42491150	-1.65000010	1	0.87672997	6.37491131	-0.73840004
1	1.37222993	4.84121132	-2.58920002	1	-3.48076987	-2.41038871	3.06970000
6	0.74352998	5.30351114	-0.60850006	1	-1.86117005	-2.60628867	3.78769994
6	0.28412998	4.78981161	0.59969997	1	-2.39406991	-1.00798869	3.25139999
1	0.06282997	5.48821115	1.40019989	8	-6.79736996	-0.36938870	-0.72100002
6	0.09932998	3.41941142	0.80479997	8	-6.49427032	-1.79158878	1.01379991
6	1.18332994	2.11901140	-2.70800018	6	-8.16586971	-0.19578870	-0.31410003
6	-0.43287003	2.91581130	2.15869999	1	-8.59667015	0.49931133	-1.03290009
6	-0.07137002	1.32341123	-3.12150002	1	-8.21137047	0.21801130	0.69509995
1	-0.47307003	0.74301130	-2.29130006	1	-8.69437027	-1.15098870	-0.33710003
1	0.16302998	0.63941133	-3.94830012	1	-5.26857042	-1.59578872	-2.57570004
1	-0.85587001	2.00681138	-3.46510005	1	-4.99477005	-3.11138868	-1.68370008
6	2.33683014	1.16451120	-2.33870006	1	-3.61386991	-2.12198877	-2.22100019
1	2.09573007	0.57661134	-1.45280004	8	5.47642994	-1.99848878	0.96289998

1	3.24863005	1.73621130	-2.13070011	6	1.33292997	-2.90648866	-1.22300005
1	2.55313015	0.48051131	-3.17100000	6	1.82812989	-3.44658852	0.11849995
6	1.64112997	2.89641142	-3.95440006	8	3.11963010	-2.83028865	0.39769995
1	2.56093001	3.46401143	-3.77660012	6	3.11812997	-1.57788873	0.84549999
1	0.87402999	3.58951139	-4.31589985	6	4.47982979	-1.05198872	1.27239990
1	1.84762990	2.18801141	-4.76550007	8	1.70802999	-3.62168860	-2.26990008
6	-0.70907003	4.06061125	3.15009999	6	1.29532993	-3.12508869	-3.56320000
1	-1.46347010	4.76131153	2.77670002	1	0.20642997	-3.10848856	-3.62540007
1	0.19622998	4.62701130	3.39369988	8	2.12453008	-0.87148869	0.92279994
1	-1.09107006	3.63891149	4.08690023	8	0.65192997	-1.89118874	-1.32540011
6	0.60162997	1.99771130	2.84029984	6	2.04223013	-4.94388866	0.15749995
1	0.89492995	1.16171122	2.20539999	6	4.43202972	-0.67958868	2.75839996
1	0.20352997	1.59471130	3.77979994	1	1.68582988	-2.11888862	-3.71730018
1	1.50932992	2.56591129	3.07399988	1	1.71562994	-3.82338858	-4.28399992
6	-1.77527010	2.18351150	1.95779991	6	6.77602959	-1.44538879	0.85119998
1	-2.53306985	2.88291144	1.58689988	1	7.14643002	-1.05388868	1.80729985
1	-2.13537002	1.76811123	2.90759993	1	6.80592966	-0.64228868	0.10149995
1	-1.69267011	1.37621129	1.22949994	1	7.43072987	-2.25638866	0.52829999
12	0.05102998	-0.60788870	0.32219994	1	2.36903000	-5.23128843	1.15929985
8	-0.47717005	-1.97818875	1.59399986	1	2.79262996	-5.26198864	-0.56810004
6	-1.79367006	-2.35858870	1.65919995	1	1.10212994	-5.45848846	-0.05730005
6	-2.58046985	-1.65978873	0.55159998	1	3.61223006	0.01471131	2.95139980
8	-3.86946988	-1.97998869	0.47359997	1	5.36592960	-0.19568869	3.05369997
6	-4.66167021	-1.31208873	-0.52510005	1	4.29652977	-1.57588875	3.36959982
6	-2.43976998	-2.07748866	3.02629995	1	4.62192965	-0.13448870	0.67999995
1	-1.92867005	-3.44128871	1.45409989	1	1.09642994	-3.11208868	0.86869997
8	-2.04257011	-0.85958868	-0.21700005				

I-0''k^2_Zn

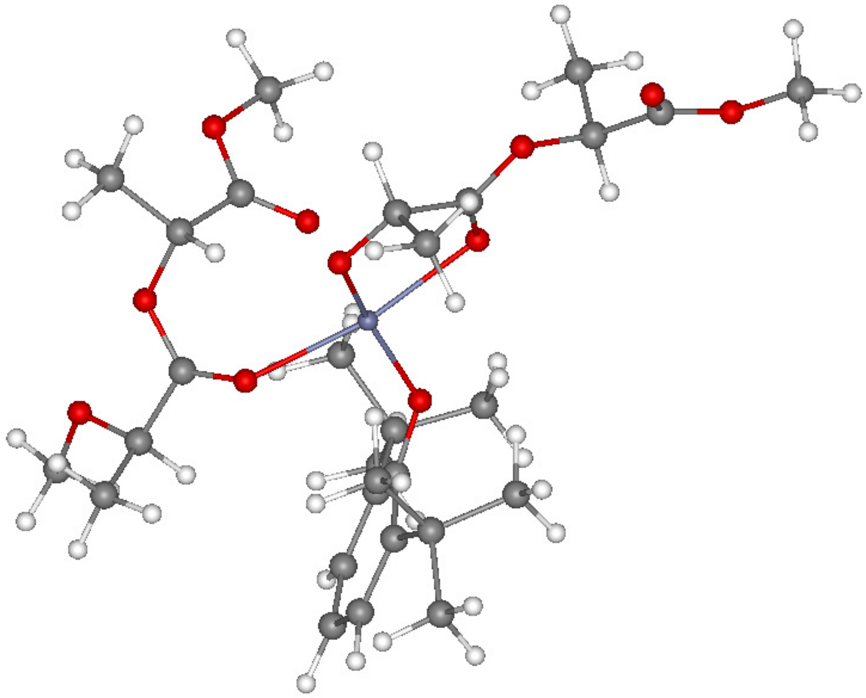

Zero-point vibrational energy	1915749.1 (Joules/Mol)
	457.87502 (Kcal/Mol)
Zero-point correction=	0.729670 (Hartree/Particle)
Thermal correction to Energy=	0.779467
Thermal correction to Enthalpy=	0.780411
Thermal correction to Gibbs Free Energy=	0.639072
Sum of electronic and zero-point Energies=	-3738.141437
Sum of electronic and thermal Energies=	-3738.091640
Sum of electronic and thermal Enthalpies=	-3738.090696
Sum of electronic and thermal Free Energies=	-3738.232035

cartesian

8	-0.36849999	-1.37950003	0.58050001	1	4.43610001	-0.71359998	1.03709996
6	-1.51810002	-2.06730008	0.52730000	6	6.38000011	-0.29660001	0.24230000
6	-2.42989993	-1.98609996	1.62899995	6	5.05490017	1.21689999	1.77419996
6	-3.68070006	-2.59960008	1.50279999	1	-5.01319981	-3.78839993	0.29100001
1	-4.40280008	-2.52889991	2.30990005	1	4.07410002	0.34590000	-3.11820006
6	-4.03060007	-3.32870007	0.37009999	1	2.54710007	0.67710000	-3.97490001
6	-3.07789993	-3.52670002	-0.62339997	1	2.69560003	-0.77679998	-2.97970009
1	-3.33899999	-4.16830015	-1.45829999	8	6.95879984	-0.98409998	1.24600005
6	-1.80420005	-2.94880009	-0.56250000	8	6.89909983	-0.07570000	-0.82639998
6	-2.04279995	-1.30299997	2.95589995	6	8.28890038	-1.46070004	0.97719997
6	-0.75510001	-3.32559991	-1.63220000	1	8.59930038	-1.98090005	1.88199997
6	-0.72479999	-1.90540004	3.48699999	1	8.28230000	-2.14299989	0.12500000
1	0.08980000	-1.76709998	2.77489996	1	8.95800018	-0.62440002	0.76499999
1	-0.44790000	-1.42780006	4.43540001	1	5.58010006	0.82270002	2.64680004
1	-0.84210002	-2.97869992	3.67330003	1	5.57730007	2.10800004	1.41610003

6	-1.88279998	0.21619999	2.78160000	1	4.04099989	1.49020004	2.07430005
1	-1.08640003	0.45130000	2.07859993	8	-4.96309996	2.05209994	-0.21439999
1	-2.81430006	0.67030001	2.42240000	6	-0.46140000	3.16249990	0.30039999
1	-1.63390005	0.68529999	3.74270010	6	-1.19110000	3.22429991	-1.03960001
6	-3.10380006	-1.51849997	4.04949999	8	-2.54259992	2.74110007	-0.79939997
1	-4.06160021	-1.04589999	3.80430007	6	-2.75780010	1.42960000	-0.92240000
1	-3.28449988	-2.58030009	4.24760008	6	-4.21190023	1.00820005	-0.79610002
1	-2.75250006	-1.06560004	4.98350000	8	-0.34639999	4.33909988	0.90560001
6	-1.20480001	-4.54710007	-2.45889997	6	0.30309999	4.32749987	2.19339991
1	-1.44889998	-5.40490007	-1.82360005	1	1.32790005	3.96639991	2.09310007
1	-2.06949997	-4.33160019	-3.09570003	8	-1.88530004	0.60890001	-1.15579998
1	-0.38780001	-4.84880018	-3.12369990	8	-0.03260000	2.12360001	0.78369999
6	-0.52440000	-2.18759990	-2.64289999	6	-1.31200004	4.60260010	-1.65219998
1	-0.03870000	-1.31050003	-2.21079993	6	-4.72809982	0.58780003	-2.17729998
1	0.12700000	-2.53040004	-3.45639992	1	-0.24529999	3.68460011	2.88310003
1	-1.46940005	-1.85469997	-3.08349991	1	0.28889999	5.36189985	2.53099990
6	0.57880002	-3.72379994	-0.96679997	6	-6.16330004	1.61389995	0.39809999
1	0.43599999	-4.59399986	-0.31639999	1	-6.88740015	1.22940004	-0.33210000
1	1.31309998	-3.99679995	-1.73520005	1	-5.96880007	0.83579999	1.14880002
1	0.98830003	-2.91619992	-0.35949999	1	-6.59539986	2.48600006	0.89179999
30	0.19870000	0.12210000	-0.39629999	1	-1.82720006	4.52379990	-2.61240005
8	1.04020000	1.05540001	-1.82889998	1	-1.86899996	5.28160000	-1.00460005
6	2.41880012	1.07529998	-1.87080002	1	-0.31750000	5.01959991	-1.82869995
6	3.02090001	0.54490000	-0.57290000	1	-4.08939981	-0.19140001	-2.59800005
8	4.34590006	0.68110001	-0.49239999	1	-5.73960018	0.18390000	-2.09100008
6	4.99230003	0.15680000	0.68000001	1	-4.75239992	1.44659996	-2.85400009
6	2.98440003	0.27559999	-3.05760002	1	-4.19129992	0.12570000	-0.13980000
1	2.80049992	2.10999990	-1.96050000	1	-0.65910000	2.52920008	-1.69809997
8	2.34619999	0.03460000	0.31729999				

I-0''k^3_Mg

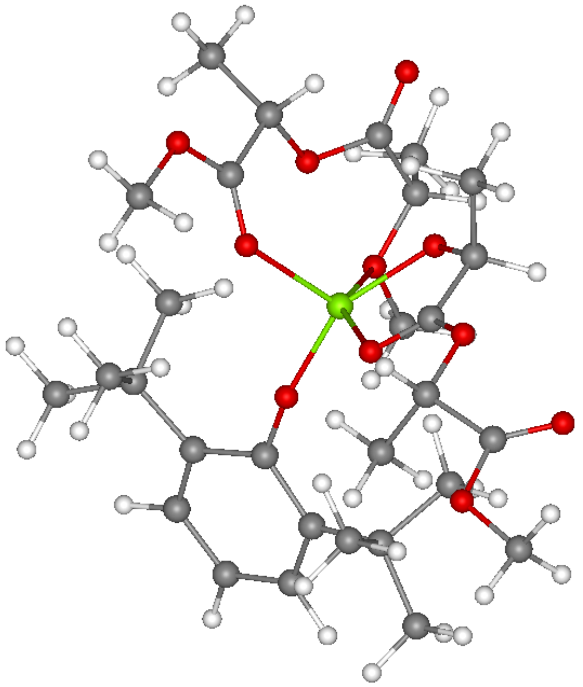

Zero-point vibrational energy	1918742.9 (Joules/Mol)
	458.59055 (Kcal/Mol)
Zero-point correction=	0.730811 (Hartree/Particle)
Thermal correction to Energy=	0.779749
Thermal correction to Enthalpy=	0.780693
Thermal correction to Gibbs Free Energy=	0.643622
Sum of electronic and zero-point Energies=	-2159.188147
Sum of electronic and thermal Energies=	-2159.139209
Sum of electronic and thermal Enthalpies=	-2159.138265
Sum of electronic and thermal Free Energies=	-2159.275336

cartesian

8	0.80801147	0.99192524	-0.24661033	6	-3.65964389	0.56607938	-1.04852605
6	1.35941148	2.19682550	-0.23361035	6	-5.85748863	-0.25167468	-0.22311033
6	0.69121146	3.29932547	0.40008965	1	-4.61061382	-1.27728975	-1.70896876
6	1.37131143	4.51582527	0.51808965	1	3.15531158	5.65932560	0.11418965
1	0.89701152	5.35192537	1.02088964	8	-6.44588852	0.59462529	-1.09081042
6	2.64661145	4.70532560	-0.00231035	8	-6.33778858	-0.59637469	0.83068967
6	3.24041152	3.66982532	-0.71591032	6	-7.73228884	1.09392524	-0.68501031
1	4.21081114	3.85232544	-1.16561043	1	-8.05478859	1.75332534	-1.48921037
6	2.62521148	2.42402530	-0.87191033	1	-7.64638853	1.64582527	0.25298965
6	-0.77128851	3.20062542	0.88028967	1	-8.43728828	0.27032530	-0.55601031
6	3.29951143	1.34852529	-1.74601042	8	1.94261146	-0.79837471	1.97228956
6	-0.93648851	2.20972538	2.04758978	6	0.95341146	-3.01277447	-1.63851035
1	-0.63808852	1.19572532	1.77868962	6	1.66641152	-3.77317452	-0.53481030
1	-1.98398852	2.17042542	2.37108970	8	1.92641139	-2.78357458	0.46118963
1	-0.33408853	2.52182531	2.90788984	6	1.81111145	-3.17647457	1.75988960

6	-1.66538846	2.78802538	-0.30681035
1	-1.35058856	1.83572519	-0.73291034
1	-1.62098849	3.54942536	-1.09351039
1	-2.71098852	2.69962549	0.01698965
6	-1.31088853	4.54982519	1.39038956
1	-1.26338851	5.32982540	0.62348968
1	-0.77708852	4.90562534	2.27828979
1	-2.36358857	4.43202543	1.67188966
6	4.54421139	1.88502538	-2.47661018
1	5.34451103	2.17232537	-1.78611040
1	4.31091118	2.74642539	-3.11131024
1	4.94731140	1.09852529	-3.12541032
6	2.32241154	0.87422526	-2.84181023
1	1.40211153	0.47542530	-2.41491032
1	2.79551148	0.09742530	-3.45781016
1	2.05861139	1.70862532	-3.50151038
6	3.77131152	0.15792532	-0.89191031
1	4.52271128	0.48452532	-0.16381034
1	4.23481131	-0.60987467	-1.52611041
1	2.95431137	-0.30087468	-0.33701035
12	0.26721144	-0.73507470	0.32158965
8	-0.59538853	-1.96067476	1.55268967
6	-1.94638848	-2.18537474	1.51448965
6	-2.55848861	-1.35057473	0.39258963
8	-3.87658858	-1.48127472	0.23568965
6	-4.50928879	-0.69077468	-0.78391033
1	-2.44849133	-1.97081125	2.46940088
6	-2.28685188	-3.64129233	1.14572370
8	-1.86778855	-0.62147468	-0.31811035
6	2.02231145	-2.01507473	2.71458983
8	0.87901151	-3.69377470	-2.77091026
6	0.19071145	-3.04067469	-3.86201024
1	-0.85098851	-2.86287451	-3.59181023
8	1.65271151	-4.32297468	2.10418963
8	0.47511148	-1.89627469	-1.49481034
6	2.94781137	-4.46587467	-0.97611028
6	3.35371137	-2.20577455	3.44298983
1	0.67701149	-2.09347463	-4.09601068
1	0.26111144	-3.73357463	-4.69781065
6	2.15421152	0.38062531	2.75458980
1	3.19091153	0.43842530	3.09918976
1	1.47281146	0.40312529	3.61388969
1	1.95131159	1.22692525	2.10098982
1	3.37751150	-4.99327469	-0.12241034
1	3.67301154	-3.73867464	-1.34831035
1	2.73481154	-5.19137478	-1.76351035
1	3.39031148	-3.21557450	3.85848975
1	3.45651150	-1.50117469	4.27088928
1	4.19671106	-2.07237458	2.75888968
1	1.18511152	-2.06487465	3.41958976
1	0.96031153	-4.50727463	-0.12901035
1	-3.37924910	-3.76926160	1.12862337
1	-1.84968305	-4.32042551	1.89249086
1	-1.87475717	-3.87389731	0.15271187
1	-4.13944387	1.17535388	-1.82863903
1	-2.65565062	0.26608065	-1.38318741
1	-3.57708430	1.15435457	-0.12272048

I-0''k^3_Zn

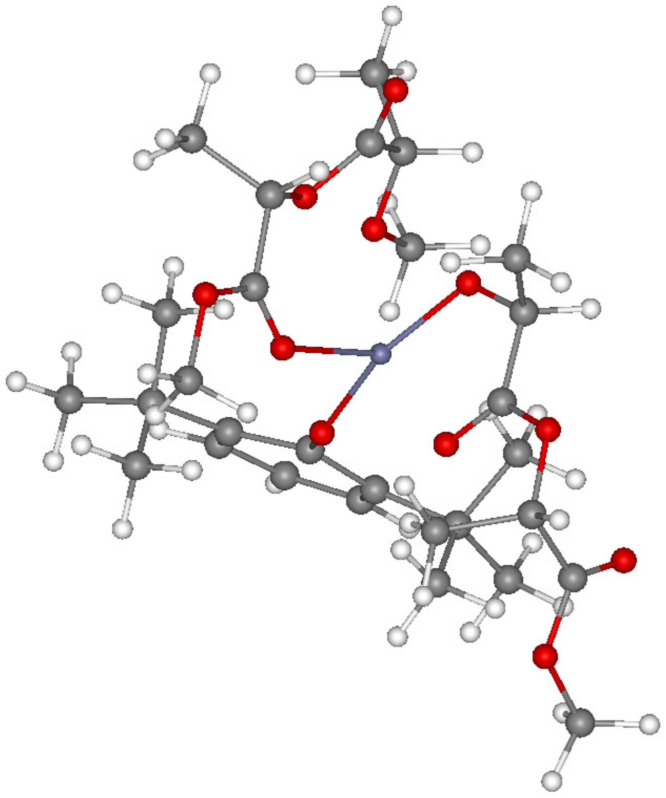

Zero-point vibrational energy	1917316.5 (Joules/Mol)
	458.24965 (Kcal/Mol)
Zero-point correction=	0.730267 (Hartree/Particle)
Thermal correction to Energy=	0.779681
Thermal correction to Enthalpy=	0.780625
Thermal correction to Gibbs Free Energy=	0.643575
Sum of electronic and zero-point Energies=	-3738.147122
Sum of electronic and thermal Energies=	-3738.097709
Sum of electronic and thermal Enthalpies=	-3738.096765
Sum of electronic and thermal Free Energies=	-3738.233814

cartesian

8	0.61922878	0.98534709	-0.62834138	6	-3.89766455	-1.17755914	-1.94317114
6	1.32802892	2.09384704	-0.37544140	6	-5.14087105	0.19884710	-0.29454139
6	0.76962882	3.17024708	0.38575861	1	-5.62190104	-1.87560725	-0.78619587
6	1.59372890	4.26044703	0.69115859	1	3.52092886	5.20554733	0.49315861
1	1.20682883	5.07634735	1.29215860	8	-5.65687084	0.80884713	-1.37494135
6	2.90252876	4.35004711	0.23195861	8	-5.04137087	0.69814706	0.80185860
6	3.39072871	3.35814691	-0.61204141	6	-6.10717106	2.15964699	-1.16494143
1	4.39112902	3.47584701	-1.01514137	1	-6.50377083	2.48594713	-2.12514138
6	2.62912869	2.23724699	-0.95614141	1	-5.27197123	2.79214692	-0.86024141
6	-0.70787120	3.19304705	0.82855862	1	-6.88377094	2.18654704	-0.39804140
6	3.18372869	1.21904719	-1.97124147	8	2.09112883	-0.70825291	1.89305866
6	-0.98017120	2.17454696	1.94985867	6	0.67332882	-3.07075310	-1.59114134
1	-0.74447119	1.14824712	1.66045856	6	1.45772886	-3.78195310	-0.50114137

1	-2.03887129	2.18904710	2.23435855
1	-0.38647121	2.40984702	2.83975863
6	-1.62857115	2.92734694	-0.37984139
1	-1.42957115	1.96044719	-0.84014142
1	-1.48667109	3.70504713	-1.13884139
1	-2.67717123	2.95134711	-0.05914139
6	-1.12087119	4.56624746	1.39215863
1	-0.95027119	5.37294722	0.67155862
1	-0.59847116	4.81804705	2.32145858
1	-2.19197130	4.54954720	1.62245858
6	4.51132917	1.68534708	-2.59564137
1	5.31802893	1.76234710	-1.85864139
1	4.41322899	2.65144706	-3.10224128
1	4.83102894	0.95304710	-3.34584141
6	2.18682885	1.03864717	-3.13544130
1	1.22042882	0.68014705	-2.77964139
1	2.58712864	0.32104707	-3.86374140
1	2.03072882	1.99014723	-3.65624142
6	3.46082878	-0.13475290	-1.29614139
1	4.21762896	-0.03025290	-0.51114142
1	3.83762884	-0.85625291	-2.03374147
1	2.56492877	-0.54845291	-0.83684140
30	0.09032881	-0.55515289	0.28805861
8	-0.59337121	-1.68995285	1.63955855
6	-1.93167114	-2.02455282	1.67035866
6	-2.68427134	-1.39435291	0.50575864
8	-3.95857120	-1.79665291	0.40465862
6	-4.74027109	-1.23095286	-0.65524137
1	-2.40504122	-1.74034476	2.62175059
6	-2.12537313	-3.54216337	1.49446833
8	-2.16797137	-0.61815292	-0.28784138
8	1.82712889	-2.76315308	0.42595860
6	1.74192882	-3.07985306	1.74185860
6	2.12632871	-1.91715288	2.63975859
8	0.50632882	-3.83385301	-2.66734147
6	-0.25557119	-3.24675298	-3.74404144
1	-1.26577115	-3.01205301	-3.40514135
8	1.48052883	-4.18715286	2.15485859
8	0.21532881	-1.94305289	-1.49974144
6	2.68582869	-4.53015280	-1.00254142
6	3.49642873	-2.20745301	3.26025867
1	0.23192880	-2.33645296	-4.09484148
1	-0.27527118	-4.00375271	-4.52554131
6	2.27872872	0.45834708	2.69255853
1	3.29682875	0.50694710	3.09345865
1	1.55622888	0.48544705	3.51855850
1	2.11992884	1.31674719	2.04095864
1	3.17132878	-5.01955271	-0.15584140
1	3.39452863	-3.84265304	-1.46984136
1	2.39792871	-5.29285288	-1.72834134
1	3.48792863	-3.20195293	3.71255851
1	3.73352885	-1.48525286	4.04455853
1	4.28072882	-2.16765285	2.49895859
1	1.35512888	-1.89255285	3.41895866
1	0.77222878	-4.48145294	-0.00804139
1	-3.19888258	-3.78042936	1.52287316
1	-1.60997295	-4.07419014	2.30767822
1	-1.70559132	-3.85563469	0.52724600
1	-4.49798107	-0.74336433	-2.75624871
1	-3.58670497	-2.19565582	-2.22026944
1	-3.00658131	-0.55552757	-1.77273834

I-1_Mg

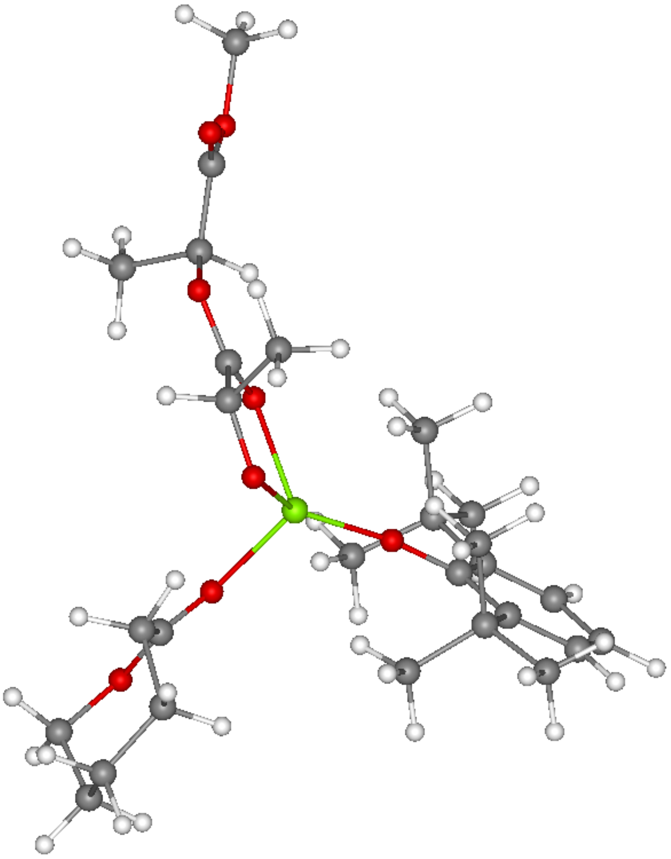

Zero-point vibrational energy	1744226.9 (Joules/Mol)
	416.88024 (Kcal/Mol)
Zero-point correction=	0.664341 (Hartree/Particle)
Thermal correction to Energy=	0.705700
Thermal correction to Enthalpy=	0.706644
Thermal correction to Gibbs Free Energy=	0.584697
Sum of electronic and zero-point Energies=	-1855.042185
Sum of electronic and thermal Energies=	-1855.000826
Sum of electronic and thermal Enthalpies=	-1854.999882
Sum of electronic and thermal Free Energies=	-1855.121829

cartesian

8	1.01289999	1.66999996	-1.12650001	1	5.19140005	-1.99300003	2.61120009
6	1.46860003	2.81509995	-1.11969995	1	4.31459999	-1.20869994	3.92689991
8	1.95980000	3.25460005	-2.27239990	6	0.59990001	-4.26630020	-3.10890007
6	1.49140000	3.67980003	0.11100000	1	1.53659999	-4.32340002	-3.67359996
6	2.54839993	4.57040024	-2.40350008	1	0.42340001	-5.23780012	-2.63479996
6	2.91370010	3.95530009	0.63419998	1	-0.20649999	-4.10650015	-3.83410001
6	3.84840012	4.74160004	-1.63180006	6	-0.78939998	-3.11980009	-1.42120004
1	2.72560000	4.64659977	-3.47679996	1	-0.88970000	-2.31999993	-0.68730003
1	2.81879997	4.27019978	1.67799997	1	-1.57790005	-3.00110006	-2.17549992
1	3.48350000	3.01959991	0.65219998	1	-0.95679998	-4.07229996	-0.90600002
1	0.98720002	4.62939978	-0.11150000	6	0.80720001	-1.81579995	-2.90079999
1	0.89190000	3.15400004	0.86220002	1	1.75100005	-1.86010003	-3.45569992

6	3.67289996	5.03100014	-0.14090000	1	-0.00420000	-1.68239999	-3.62779999
1	1.80579996	5.32819986	-2.12989998	1	0.84480000	-0.93390000	-2.26099992
1	4.46490002	3.84899998	-1.79050004	12	0.17260000	0.35839999	0.22229999
1	4.38810015	5.57539988	-2.09610009	8	-0.39430001	1.54770005	1.62380004
1	3.14739990	5.98890018	-0.02710000	6	-1.74679995	1.66849995	1.82009995
1	4.66029978	5.16930008	0.31330001	6	-2.49729991	0.95810002	0.69270003
8	1.06099999	-1.26010001	0.09670000	8	-3.81469989	1.09389997	0.70200002
6	1.86539996	-2.31369996	0.02700000	6	-4.57270002	0.41540000	-0.31920001
6	2.88980007	-2.50659990	1.00800002	6	-2.21569991	1.11389995	3.17729998
6	3.72280002	-3.62360001	0.89310002	1	-2.08690000	2.72250009	1.76300001
1	4.50710011	-3.79130006	1.62409997	8	-1.90040004	0.30190000	-0.17240000
6	3.58100009	-4.54659986	-0.13720000	1	-4.03929996	-0.49000001	-0.61799997
6	2.57540011	-4.35839987	-1.07980001	6	-5.89529991	0.01460000	0.32499999
1	2.47569990	-5.09299994	-1.87230003	6	-4.77159977	1.32900000	-1.52260005
6	1.70469999	-3.26600003	-1.02950001	1	4.24300003	-5.40689993	-0.20190001
6	3.08380008	-1.52190006	2.17569995	1	-3.29089999	1.24389994	3.33049989
6	0.60009998	-3.11179996	-2.09109998	1	-1.67299998	1.64709997	3.96129990
6	3.46519995	-0.12639999	1.63979995	1	-1.96500003	0.05180000	3.25950003
1	2.74889994	0.23130000	0.89749998	8	-6.56930017	-0.79290003	-0.51490003
1	3.51469994	0.60280001	2.45799994	8	-6.29199982	0.36430001	1.41129994
1	4.44600010	-0.16220000	1.15240002	6	-7.85109997	-1.24230003	-0.03960000
6	1.80960000	-1.44970000	3.04169989	1	-8.24890041	-1.87409997	-0.83200002
1	0.93720001	-1.13380003	2.46950006	1	-7.73369980	-1.81200004	0.88410002
1	1.58949995	-2.43429995	3.46849990	1	-8.50979996	-0.39070001	0.14160000
1	1.94669998	-0.74360001	3.86960006	1	-5.38030005	0.82400000	-2.27539992
6	4.22270012	-1.94509995	3.12019992	1	-5.26999998	2.25489998	-1.22430003
1	4.03079987	-2.91689992	3.58710003	1	-3.80310011	1.57040000	-1.96619999

I-1_Zn

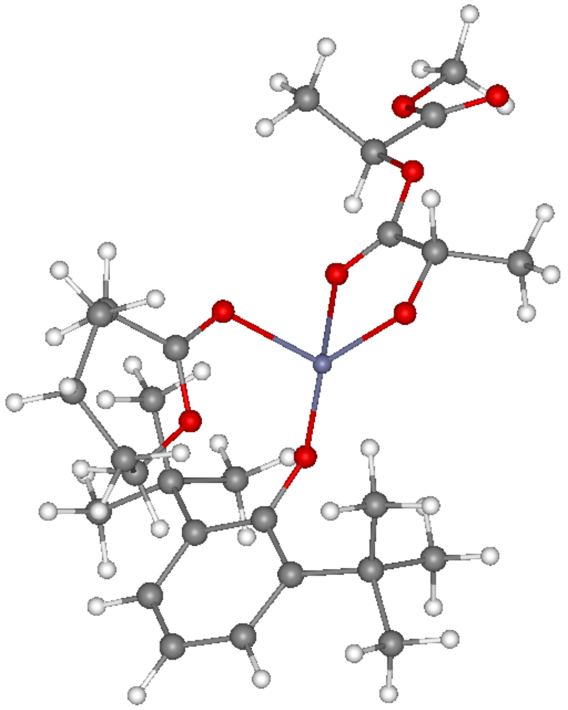

Zero-point vibrational energy	1742262.5 (Joules/Mol)
	416.41074 (Kcal/Mol)
Zero-point correction=	0.663593 (Hartree/Particle)
Thermal correction to Energy=	0.705320
Thermal correction to Enthalpy=	0.706264
Thermal correction to Gibbs Free Energy=	0.583538
Sum of electronic and zero-point Energies=	-3434.003343
Sum of electronic and thermal Energies=	-3433.961615
Sum of electronic and thermal Enthalpies=	-3433.960671
Sum of electronic and thermal Free Energies=	-3434.083397

cartesian

8	-0.35839999	2.01460004	0.34869999	1	-4.71360016	-1.63849998	-3.10039997
6	-1.52709997	2.30649996	0.61339998	1	-3.55749989	-2.38039994	-4.20170021
8	-2.47679996	1.50750005	0.16159999	6	-1.74930000	-2.25830007	4.04040003
6	-1.86350000	3.54800010	1.39569998	1	-2.46469998	-1.52030003	4.42089987
6	-3.87010002	1.64080000	0.54619998	1	-2.23740005	-3.23839998	4.03249979
6	-2.75060010	4.54680014	0.63099998	1	-0.92530000	-2.30999994	4.76070023
6	-4.54860020	2.84410000	-0.08500000	6	-0.13160001	-2.94379997	2.29649997
1	-4.30219984	0.70270002	0.19830000	1	0.33329999	-2.73020005	1.33340001
1	-2.64420009	5.51949978	1.12249994	1	0.65060002	-2.97709990	3.06520009
1	-2.36039996	4.67490005	-0.38550001	1	-0.59329998	-3.93600011	2.24370003
1	-2.35030007	3.25539994	2.33450007	6	-0.50559998	-0.50730002	2.82340002
1	-0.90700001	4.00339985	1.65540004	1	-1.24000001	0.26240000	3.09089994
6	-4.23430014	4.17929983	0.59009999	1	0.23909999	-0.54710001	3.62800002
1	-3.93409991	1.65050006	1.63909996	1	0.00940000	-0.19790000	1.91460001
1	-4.28840017	2.87210011	-1.14960003	30	0.15979999	0.22059999	-0.70770001

1	-5.62909985	2.66499996	-0.03640000	8	0.99339998	0.85850000	-2.28250003
1	-4.62179995	4.15749979	1.61769998	6	2.36590004	0.95429999	-2.24670005
1	-4.78079987	4.97749996	0.07570000	6	2.91849995	0.60250002	-0.86809999
8	-0.71319997	-1.33980000	-0.14520000	8	4.23150015	0.77039999	-0.73820001
6	-1.97140002	-1.63409996	0.19540000	6	4.82679987	0.43130001	0.52640003
6	-2.97819996	-1.82760000	-0.80330002	6	3.03699994	0.06280000	-3.30649996
6	-4.29120016	-2.06410003	-0.37650001	1	2.70300007	1.99399996	-2.42429996
1	-5.08090019	-2.19619989	-1.10860002	8	2.20650005	0.19580001	0.05380000
6	-4.62760019	-2.15840006	0.97060001	1	4.24420023	-0.36530000	0.99540001
6	-3.61919999	-2.07800007	1.92680001	6	6.22119999	-0.10190000	0.21850000
1	-3.88870001	-2.21959996	2.96830010	6	4.86740017	1.65380001	1.43640006
6	-2.28559995	-1.84420002	1.57640004	1	-5.65700006	-2.34330010	1.26919997
6	-2.65869999	-1.82840002	-2.31369996	1	4.12500000	0.17280000	-3.31279993
6	-1.18750000	-1.87779999	2.65890002	1	2.64059997	0.34860000	-4.28350019
6	-2.32879996	-0.41460001	-2.82789993	1	2.78020000	-0.98689997	-3.13369989
1	-1.40439999	-0.00890000	-2.41319990	8	6.75169992	-0.62889999	1.33940005
1	-2.18889999	-0.42860001	-3.91499996	8	6.78079987	-0.06000000	-0.85140002
1	-3.14059997	0.28479999	-2.60060000	6	8.08100033	-1.15980005	1.19799995
6	-1.49179995	-2.79020000	-2.62019992	1	8.34799957	-1.54050004	2.18260002
1	-0.58359998	-2.50850010	-2.08809996	1	8.09160042	-1.96350002	0.45940000
1	-1.75440001	-3.81270003	-2.32719994	1	8.77390003	-0.37520000	0.88779998
1	-1.28020000	-2.79089999	-3.69619989	1	5.35059977	1.39859998	2.38190007
6	-3.85660005	-2.31940007	-3.14969993	1	5.42059994	2.46810007	0.96130002
1	-4.19070005	-3.31660008	-2.84439993	1	3.84899998	1.98870003	1.64520001

I-1'_Mg

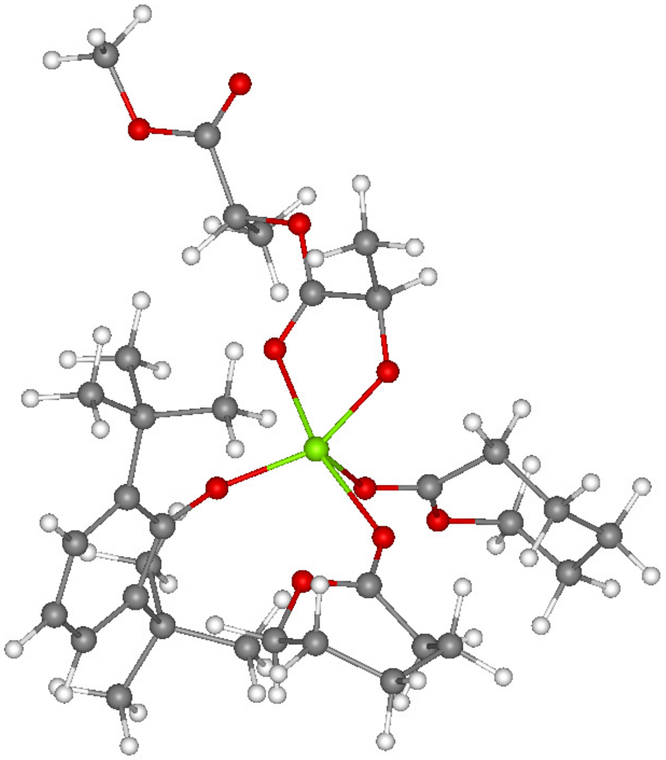

Zero-point vibrational energy	2160679.6 (Joules/Mol)
	516.41482 (Kcal/Mol)
Zero-point correction=	0.822959 (Hartree/Particle)
Thermal correction to Energy=	0.873200
Thermal correction to Enthalpy=	0.874144
Thermal correction to Gibbs Free Energy=	0.733456
Sum of electronic and zero-point Energies=	-2239.913590
Sum of electronic and thermal Energies=	-2239.863349
Sum of electronic and thermal Enthalpies=	-2239.862405
Sum of electronic and thermal Free Energies=	-2240.003094

cartesian

8 -0.20738840 -1.11118734 -0.66721988	1 4.51151180 1.38461268 -1.60311985 8 -
6 -0.94868839 -2.15068746 -0.98971987	1.63388836 1.20891261 0.86798012
6 -0.94908845 -3.33648753 -0.17561986	6 -2.73298836 0.67161262 1.00168014
6 -1.85488844 -4.35848713 -0.47811985	6 -3.97528863 1.48421264 1.27448010
1 -1.88938832 -5.24858713 0.14218014	8 -2.80088830 -0.64468741 0.93068016
6 -2.71818829 -4.28878736 -1.56831980	6 -4.67398834 1.13631272 2.60108018
6 -2.62978840 -3.19198751 -2.42131996	1 -4.67388821 1.35861266 0.43768016
1 -3.26268840 -3.18058753 -3.30321980	1 -3.65558839 2.52691245 1.27428019
6 -1.75178838 -2.13028741 -2.18271995	6 -4.05008793 -1.37358737 0.87898016
6 0.04481159 -3.52488756 0.98728013	1 -3.92328858 1.05611265 3.39618015
6 -1.64498842 -0.98528737 -3.20871997	1 -5.31218815 1.98531270 2.86858010
6 -0.24718839 -2.55668736 2.14838004	1 -4.67238808 -0.95958740 0.07838015
1 -0.15428841 -1.50998735 1.86028016	1 -3.73188829 -2.36968756 0.57008016
1 0.44871160 -2.73308754 2.97798014	6 -5.53618813 -0.12568736 2.55548024

1	-1.26408839	-2.70718741	2.52778006
6	1.48751163	-3.33128738	0.47588015
1	1.62001157	-2.35518742	0.00878015
1	1.73061156	-4.09848738	-0.26811984
1	2.20131159	-3.42868733	1.30438018
6	-0.01758841	-4.94348717	1.58418012
1	0.17231160	-5.71668720	0.83228016
1	-0.98028839	-5.15718699	2.06228018
1	0.75291157	-5.04058743	2.35758018
6	-2.45538831	-1.26688731	-4.48681974
1	-3.53348851	-1.32318735	-4.29851961
1	-2.14288831	-2.19558740	-4.97581959
1	-2.29548836	-0.44998735	-5.19991970
6	-0.17728841	-0.80478740	-3.64951992
1	0.47401160	-0.59938741	-2.80011988
1	-0.09358841	0.02441263	-4.36321974
1	0.18261160	-1.71398735	-4.14431953
6	-2.18758845	0.33201262	-2.62361979
1	-3.24838829	0.22501263	-2.36311984
1	-2.10218835	1.14481270	-3.35581994
1	-1.63878846	0.62831259	-1.73151982
12	0.32211161	0.46681264	0.26668015
8	1.10661161	1.09461272	1.93238020
6	2.45671153	0.98931259	2.13168025
6	3.12471151	0.46081263	0.86258012
8	4.45461178	0.35811263	0.91148013
6	5.11521149	-0.17528737	-0.24861985
6	2.83011150	0.10441263	3.33418012
1	2.93721151	1.97751272	2.30298018
8	2.47041154	0.16321264	-0.13601986
1	4.46851158	-0.92018741	-0.71851987
6	6.37771130	-0.86528736	0.25268015
6	5.43901157	0.93371260	-1.24441981
1	-3.41338849	-5.09878731	-1.77691984
1	-6.33898830	0.02091263	1.81988013
1	-6.03148842	-0.24998736	3.52518010
6	-4.77868843	-1.40828729	2.21218014
1	-4.05508804	-1.65688729	2.99718022
1	-5.48828793	-2.24298739	2.17038012
8	0.40711159	2.18221259	-0.97291988
6	0.49301159	3.38761258	-0.76321983
6	0.58641160	3.97951245	0.61838013
8	0.48861158	4.17691278	-1.84181988
6	-0.64248842	4.81911278	1.00888014
1	1.49441159	4.59321260	0.68758017
1	0.70331156	3.12901258	1.30088019
6	0.59141159	5.61271286	-1.73611987
1	-1.55008841	4.26821280	0.73838013
1	-0.64898843	4.89541292	2.10088015
1	1.51261163	5.87711287	-1.20451987
1	0.70571160	5.92441273	-2.77521992
6	-0.66918838	6.22661257	0.41398016
1	0.18551159	6.79421282	0.80658013
1	-1.56688845	6.74901295	0.76428014
6	-0.62928838	6.27471256	-1.11321986
1	-1.52738845	5.81711292	-1.54511988
1	-0.62978840	7.32221270	-1.43781984
1	3.91071153	0.06341264	3.50058007
1	2.34501171	0.52101260	4.22068024
1	2.44931149	-0.91038740	3.18828011
8	6.89381123	-1.62118733	-0.73701984
8	6.86651134	-0.75138742	1.35218012
6	8.11251163	-2.30978751	-0.41071984
1	8.38741207	-2.85708737	-1.31111979
1	7.94871140	-2.99738741	0.42128015
1	8.89371204	-1.59628737	-0.14021985
1	5.97601128	0.52031261	-2.10101986
1	6.05691147	1.70341265	-0.77391988

I-1'_Zn

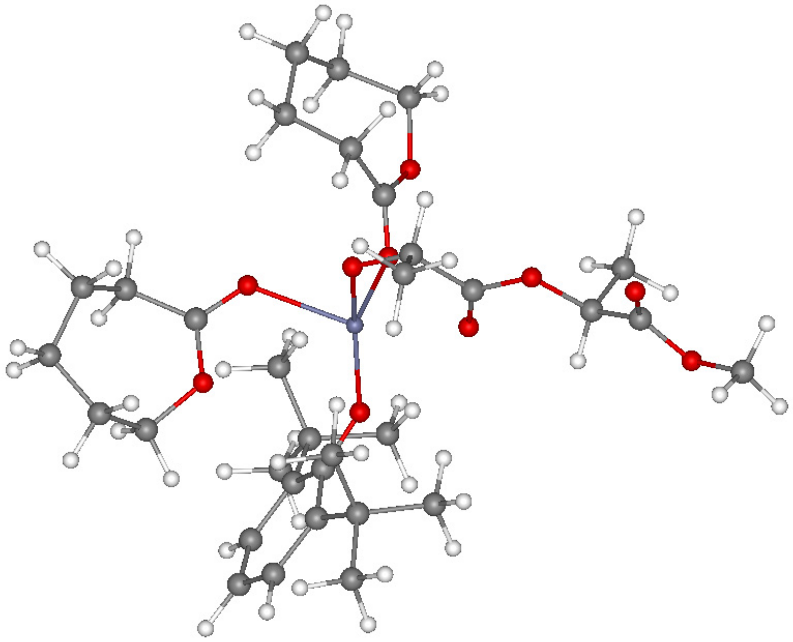

Zero-point vibrational energy	2158667.9 (Joules/Mol)
	515.93402 (Kcal/Mol)
Zero-point correction=	0.822193 (Hartree/Particle)
Thermal correction to Energy=	0.872945
Thermal correction to Enthalpy=	0.873889
Thermal correction to Gibbs Free Energy=	0.731130
Sum of electronic and zero-point Energies=	-3818.867564
Sum of electronic and thermal Energies=	-3818.816812
Sum of electronic and thermal Enthalpies=	-3818.815868
Sum of electronic and thermal Free Energies=	-3818.958627

cartesian

8	-0.55190003	-1.02680004	1.10380006	8	-1.88160002	1.26960003	-0.93049997
6	-1.49220002	-1.95029998	1.27820003	6	-2.85759997	0.63950002	-1.32869995
6	-1.40690005	-3.22729993	0.62900001	6	-4.05649996	1.33630002	-1.92480004
6	-2.47059989	-4.12500000	0.77999997	8	-2.82249999	-0.68290001	-1.25109994
1	-2.43869996	-5.08799982	0.28080001	6	-4.32740021	0.95709997	-3.39179993
6	-3.57509995	-3.83879995	1.57640004	1	-4.94110012	1.12530005	-1.31089997
6	-3.58640003	-2.65339994	2.30579996	1	-3.85130000	2.40409994	-1.83910000
1	-4.41720009	-2.47950006	2.98219991	6	-3.97129989	-1.51370001	-1.54299998
6	-2.55839992	-1.71010005	2.20889997	1	-3.38299990	0.96770000	-3.94849992
6	-0.16320001	-3.66120005	-0.17260000	1	-4.94799995	1.74769998	-3.82750010
6	-2.56590009	-0.47880000	3.13689995	1	-4.82590008	-1.16390002	-0.95420003
6	-0.04650000	-2.88759995	-1.49740005	1	-3.67319989	-2.48300004	-1.14349997
1	0.05780000	-1.81110001	-1.35650003	6	-5.04099989	-0.38190001	-3.57999992
1	0.82969999	-3.22880006	-2.06310010	1	-6.02759981	-0.32640001	-3.09990001
1	-0.93099999	-3.05290008	-2.12269998	1	-5.23089981	-0.53420001	-4.64860010
6	1.10810006	-3.46560001	0.67949998	6	-4.29020023	-1.59300005	-3.02679992

1	1.23650002	-2.42650008	0.98100001	1	-3.35489988	-1.75790000	-3.57450008
1	1.05410004	-4.08010006	1.58529997	1	-4.90049982	-2.49099994	-3.17930007
1	1.99319994	-3.78220010	0.11230000	8	0.27509999	2.05690002	1.08309996
6	-0.20829999	-5.15439987	-0.54869998	6	0.38909999	3.24289989	0.79560000
1	-0.31110001	-5.79769993	0.33149999	6	0.37120000	3.76270008	-0.61809999
1	-1.02059996	-5.39109993	-1.24520004	8	0.52719998	4.08809996	1.82550001
1	0.72920001	-5.42579985	-1.04709995	6	-0.84840000	4.64629984	-0.93470001
6	-3.69700003	-0.54409999	4.17869997	1	1.29630005	4.32460022	-0.80489999
1	-4.69309998	-0.51099998	3.72259998	1	0.38350001	2.87820005	-1.26289999
1	-3.63420010	-1.44270003	4.80200005	6	0.68470001	5.50890017	1.63320005
1	-3.62030005	0.32280001	4.84480000	1	-1.75049996	4.15889978	-0.54990000
6	-1.24100006	-0.40390000	3.92479992	1	-0.95719999	4.67000008	-2.02379990
1	-0.38409999	-0.32120001	3.25640011	1	1.56050003	5.70030022	1.00220001
1	-1.24919999	0.46689999	4.59219980	1	0.91430002	5.86590004	2.63829994
1	-1.11380005	-1.30040002	4.54230022	6	-0.74720001	6.08080006	-0.41740000
6	-2.77270007	0.82080001	2.33990002	1	0.09260000	6.58179998	-0.91829997
1	-3.72869992	0.79460001	1.80180001	1	-1.64800000	6.63280010	-0.70950001
1	-2.79390001	1.68400002	3.01719999	6	-0.55860001	6.20120001	1.09420002
1	-1.97220004	0.98130000	1.62070000	1	-1.43089998	5.81040001	1.63170004
30	0.01240000	0.27649999	-0.15320000	1	-0.48019999	7.26200008	1.36150002
8	0.78909999	0.78160000	-1.83329999	1	3.58139992	-0.21910000	-3.42880011
6	2.14790010	0.68320000	-2.03010011	1	1.99460006	0.21100000	-4.11600018
6	2.86100006	0.18070000	-0.77780002	1	2.14510012	-1.21800005	-3.08570004
8	4.19379997	0.12340000	-0.89749998	8	6.81010008	-1.68830001	0.67159998
6	4.92869997	-0.35859999	0.23830000	8	6.62979984	-0.87930000	-1.43410003
6	2.50449991	-0.19690000	-3.23939991	6	8.05090046	-2.31200004	0.30160001
1	2.60080004	1.67980003	-2.21449995	1	8.39850044	-2.82270002	1.19830000
8	2.27020001	-0.12390000	0.25060001	1	7.89099979	-3.02570009	-0.50889999
1	4.34259987	-1.12979996	0.74460000	1	8.77569962	-1.56029999	-0.01820000
6	6.20279980	-0.98949999	-0.30860001	1	5.82749987	0.40320000	2.04679990
6	5.23559999	0.77789998	1.20879996	1	5.79379988	1.57190001	0.70520002
1	-4.38840008	-4.55480003	1.67159998	1	4.30140018	1.18540001	1.60039997

I-2_Mg

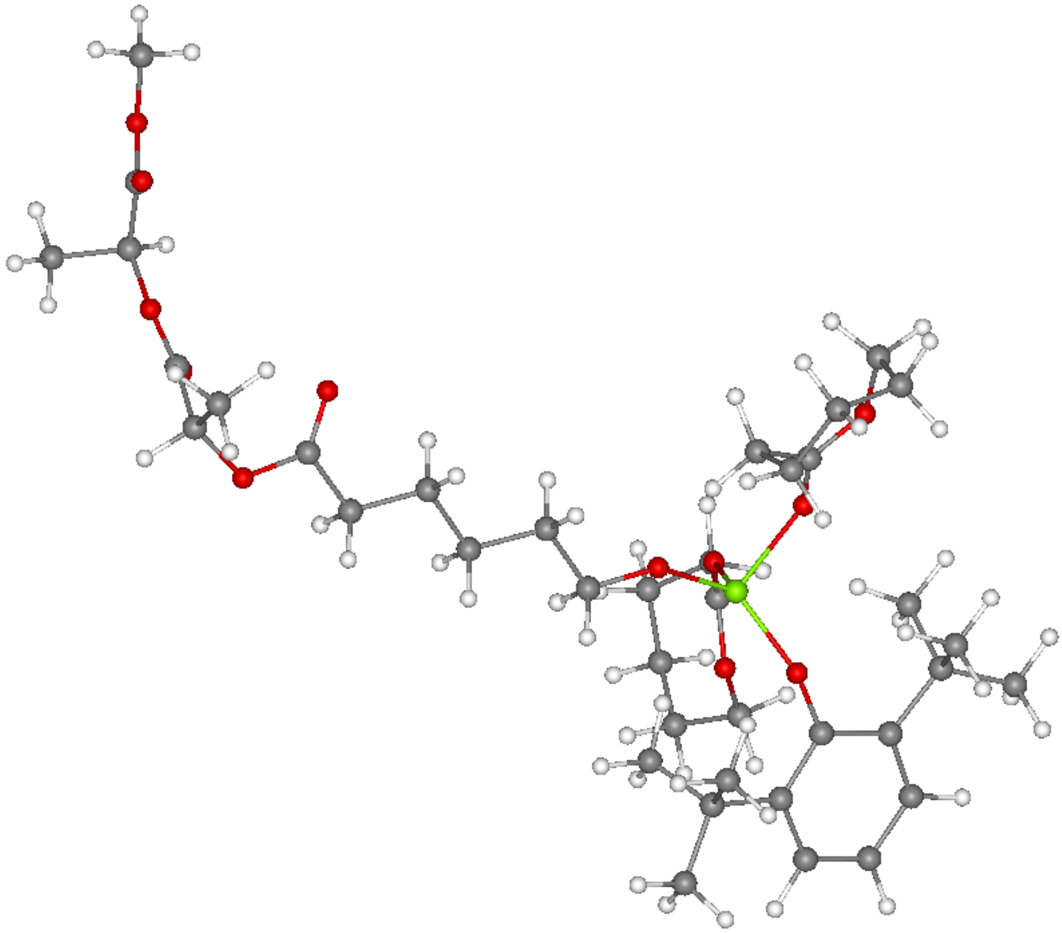

Zero-point vibrational energy	2574647.1 (Joules/Mol)
	615.35544 (Kcal/Mol)
Zero-point correction=	0.980631 (Hartree/Particle)
Thermal correction to Energy=	1.040956
Thermal correction to Enthalpy=	1.041900
Thermal correction to Gibbs Free Energy=	0.872263
Sum of electronic and zero-point Energies=	-2624.767760
Sum of electronic and thermal Energies=	-2624.707436
Sum of electronic and thermal Enthalpies=	-2624.706492
Sum of electronic and thermal Free Energies=	-2624.876129

cartesian

12	2.96390009	0.45370001	0.16230001	1	3.56419992	-3.14730000	5.31379986
8	1.18719995	0.35519999	-0.41270000	1	6.45410013	-2.36019993	2.49009991
8	4.59999990	0.22380000	-0.76730001	1	6.21929979	-3.02119994	0.86979997
6	0.24250001	-0.54869998	-0.87889999	6	4.87060022	-4.11140013	3.90969992
1	0.41580001	-0.80080003	-1.94260001	1	5.76370001	-3.86139989	4.49830008
1	0.28870001	-1.51170003	-0.33270001	1	4.55310011	-5.10470009	4.24620008
6	5.71040010	-0.43509999	-1.05239999	6	5.25600004	-4.18520021	2.43210006
6	5.67910004	-1.60389996	-1.88689995	1	4.41919994	-4.55329990	1.82710004
6	6.85820007	-2.33839989	-2.05349994	1	6.06790018	-4.91169977	2.30990005
1	6.85080004	-3.23790002	-2.66039991	6	2.31539989	3.42370009	0.72039998

6	8.06420040	-1.94939995	-1.47739995
6	8.11030006	-0.75790000	-0.75940001
1	9.06890011	-0.43640000	-0.36530000
6	6.97340012	0.02830000	-0.54850000
6	4.39769983	-2.05089998	-2.61919999
6	7.10559988	1.37310004	0.19380000
6	3.34229994	-2.57310009	-1.62730002
1	3.03200006	-1.80820000	-0.91570002
1	2.44409990	-2.90750003	-2.15960002
1	3.73589993	-3.42179990	-1.05680001
6	3.82159996	-0.88569999	-3.45040011
1	3.59640002	-0.01870000	-2.82940006
1	4.53980017	-0.57679999	-4.21829987
1	2.90039992	-1.20009995	-3.95609999
6	4.66459990	-3.20040011	-3.60949993
1	5.41809988	-2.93219995	-4.35739994
1	4.98719978	-4.12060022	-3.10969996
1	3.73749995	-3.43420005	-4.14499998
6	8.57229996	1.73909998	0.48460001
1	9.05519962	1.03209996	1.16859996
1	9.17259979	1.79690003	-0.42960000
1	8.60690022	2.72429991	0.96359998
6	6.52990007	2.51449990	-0.66949999
1	5.48449993	2.33330011	-0.91930002
1	6.60290003	3.46930003	-0.13380000
1	7.09590006	2.60579991	-1.60329998
6	6.38719988	1.32550001	1.55509996
1	6.81990004	0.53909999	2.18619990
1	6.49830008	2.27940011	2.08529997
1	5.32149982	1.13619995	1.43920004
8	3.00370002	2.41100001	0.83980000
6	-1.18379998	-0.00560000	-0.74529999
1	8.96409988	-2.54220009	-1.62460005
6	-2.25749993	-0.97610003	-1.23699999
6	-3.67770004	-0.42309999	-1.10599995
6	-4.73579979	-1.40300000	-1.60220003
1	-3.76300001	0.51569998	-1.66569996
1	-3.88540006	-0.16930000	-0.06100000
8	3.17400002	-0.44580001	2.04660010
6	3.96409988	-1.31250000	2.43109989
6	4.10680008	-1.64429998	3.89299989
6	1.04610002	3.46029997	-0.08190000
8	2.76769996	4.51410007	1.33829999
6	1.15960002	4.32119989	-1.35290003
1	0.22970000	3.83089995	0.55150002
1	0.82950002	2.41450000	-0.33500001
6	2.07319999	5.77880001	1.24820006
1	2.09470010	4.08220005	-1.87310004
1	0.35380000	4.01300001	-2.02649999
1	1.05190003	5.66179991	1.62750006
1	2.62240005	6.40579987	1.95179999
6	1.05289996	5.82609987	-1.10979998
1	0.05120000	6.05170012	-0.71950001
1	1.13220000	6.35220003	-2.06780005
6	2.09400010	6.39340019	-0.14399999
1	3.10669994	6.28989983	-0.55190003
1	1.91890001	7.46910000	-0.02280000
1	-1.36070001	0.24640000	0.30899999
1	-1.25160003	0.93599999	-1.30760002
1	-2.06100011	-1.23459995	-2.28710008
1	-2.18149996	-1.91779995	-0.67519999
6	-6.14839983	-0.89829999	-1.44939995
1	-4.68060017	-2.35159993	-1.05330002
1	-4.57959986	-1.66579998	-2.65479994
8	-7.02489996	-1.74090004	-2.05909991
8	-6.48999977	0.10760000	-0.86449999
6	-8.41989994	-1.43550003	-1.93729997
6	-8.82839966	-1.43970001	-0.46250001
1	-8.90260029	-2.32229996	-2.36369991
6	-8.84399986	-0.21439999	-2.74289989
8	-9.99269962	-0.78189999	-0.29310000
8	-8.23540020	-2.02690005	0.41400000
6	-10.51329994	-0.74910003	1.04299998
6	-11.34560013	0.52190000	1.14960003
1	-9.68080044	-0.69080001	1.74860001
6	-11.35589981	-1.98800004	1.33150005
1	-9.93309975	-0.14139999	-2.75950003
1	-8.48649979	-0.32679999	-3.77010012
1	-8.44029999	0.70490003	-2.32010007
8	-11.57460022	0.79820001	2.44939995
8	-11.76910019	1.17770004	0.22669999
6	-12.39620018	1.95169997	2.69499993

8	4.65869999	-1.96159995	1.51810002	1	-12.48900032	2.01769996	3.77800012
6	3.76690006	-3.10520005	4.23850012	1	-11.91969967	2.84910011	2.29530001
1	5.13170004	-1.41219997	4.20879984	1	-13.37720013	1.82819998	2.23160005
1	3.44260001	-0.95810002	4.41979980	1	-11.77330017	-1.93030000	2.33960009
6	5.73990011	-2.87350011	1.83780003	1	-12.17490005	-2.07080007	0.61199999
1	2.83159995	-3.38840008	3.74119997	1	-10.72889996	-2.87960005	1.26699996

I-2_Zn

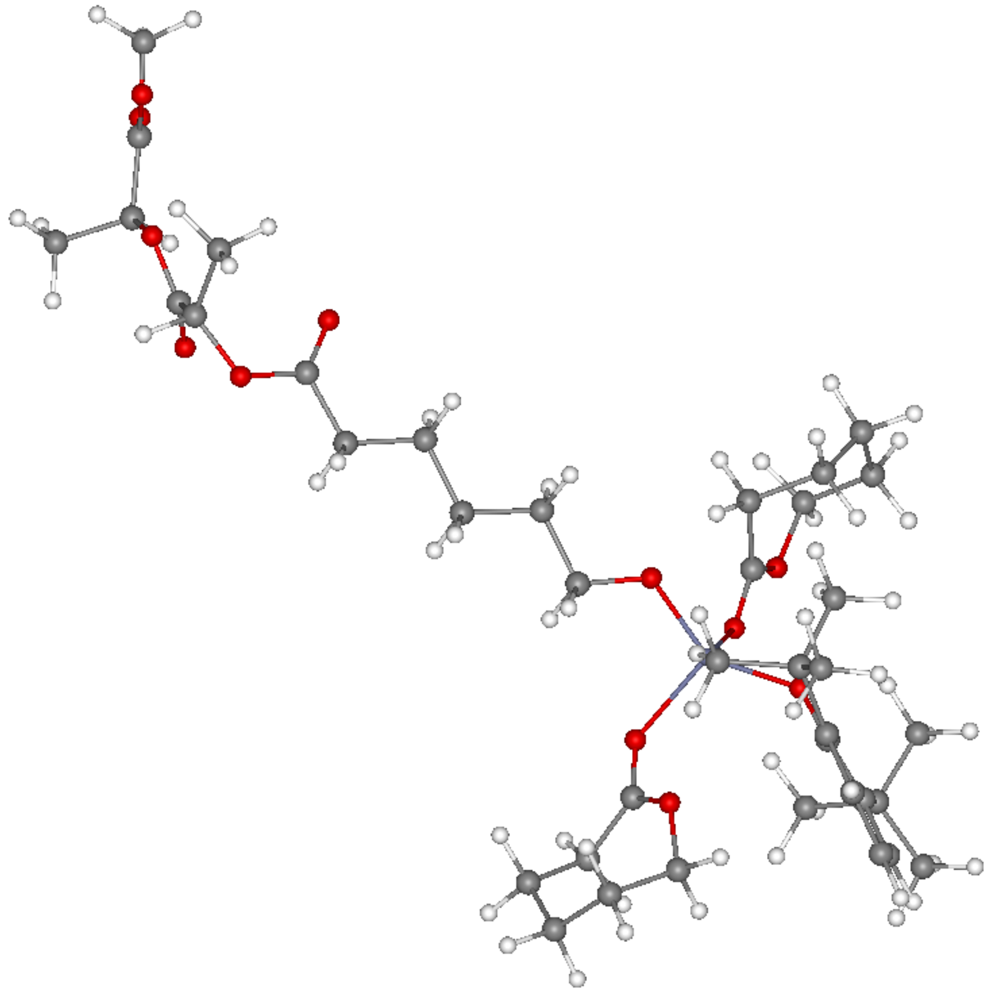

Zero-point vibrational energy	2573717.8 (Joules/Mol)
	615.13332 (Kcal/Mol)
Zero-point correction=	0.980277 (Hartree/Particle)
Thermal correction to Energy=	1.040895
Thermal correction to Enthalpy=	1.041839
Thermal correction to Gibbs Free Energy=	0.871473
Sum of electronic and zero-point Energies=	-4203.726712
Sum of electronic and thermal Energies=	-4203.666095
Sum of electronic and thermal Enthalpies=	-4203.665150
Sum of electronic and thermal Free Energies=	-4203.835516

cartesian

30	-2.81049991	0.35730001	-0.16790000	1	-2.79999995	-4.52769995	-4.09499979

8	-1.10389996	0.32010001	0.60949999
8	-4.53630018	0.65829998	0.56290001
6	-0.15940000	-0.66439998	0.29409999
1	-0.39120001	-1.62119997	0.80159998
1	-0.13770001	-0.88970000	-0.78839999
6	-5.60239983	-0.08040000	0.87419999
6	-5.62540007	-0.90369999	2.04749990
6	-6.74520016	-1.71730006	2.26209998
1	-6.77860022	-2.36789989	3.12949991
6	-7.84340000	-1.71340001	1.40820003
6	-7.86040020	-0.82290000	0.33849999
1	-8.75170040	-0.78179997	-0.27919999
6	-6.77950001	0.01900000	0.05940000
6	-4.48750019	-0.90630001	3.09089994
6	-6.89270020	1.05729997	-1.07490003
6	-3.25160003	-1.65869999	2.56399989
1	-2.79920006	-1.17270005	1.69770002
1	-2.47379994	-1.70210004	3.33590007
1	-3.51329994	-2.68689990	2.28940010
6	-4.10449982	0.53299999	3.49210000
1	-3.75119996	1.11010003	2.63870001
1	-4.96980000	1.05190003	3.91989994
1	-3.31279993	0.51120001	4.25059986
6	-4.90660000	-1.61670005	4.39309978
1	-5.81180000	-1.17949998	4.82840014
1	-5.07490015	-2.69039989	4.25509977
1	-4.10230017	-1.51400006	5.12989998
6	-8.29909992	1.08550000	-1.70029998
1	-8.55830002	0.14330000	-2.19630003
1	-9.07649994	1.31099999	-0.96249998
1	-8.33489990	1.87070000	-2.46390009
6	-6.62620020	2.47169995	-0.51849997
1	-5.63210011	2.53670001	-0.07560000
1	-6.70340014	3.21440005	-1.32229996
1	-7.36630011	2.72510004	0.24890000
6	-5.90630007	0.75199997	-2.21580005
1	-6.10239983	-0.24259999	-2.63639998
1	-6.01889992	1.48249996	-3.02609992
1	-4.87319994	0.79629999	-1.87660003
8	-2.70830011	2.17980003	-1.31840003
6	1.24080002	-0.22430000	0.72049999
1	-5.83260012	-3.13479996	-1.68869996
1	-5.59429979	-3.19510007	0.05720000
6	-4.04610014	-5.09329987	-2.44149995
1	-4.94080019	-5.14529991	-3.07649994
1	-3.62590003	-6.10510015	-2.42560005
6	-4.46689987	-4.70609999	-1.02370000
1	-3.61700010	-4.76219988	-0.33329999
1	-5.20699978	-5.42889977	-0.66100001
6	-2.21910000	3.25860000	-0.99229997
6	-1.40760005	3.45869994	0.25819999
8	-2.45919991	4.28590012	-1.81260002
6	-2.15229988	4.28749990	1.32079995
1	-0.46059999	3.94980001	0.00140000
1	-1.15939999	2.45849991	0.62790000
6	-2.01290011	5.62349987	-1.49960005
1	-3.18510008	3.93059993	1.40509999
1	-1.68139994	4.08209991	2.28719997
1	-0.92140001	5.63409996	-1.40160000
1	-2.27239990	6.18179989	-2.40019989
6	-2.12179995	5.79409981	1.06579995
1	-1.08200002	6.14260006	1.13279998
1	-2.66689992	6.30590010	1.86689997
6	-2.69829988	6.22959995	-0.28240001
1	-3.76939988	6.00299978	-0.34259999
1	-2.60780001	7.31890011	-0.37169999
1	1.48679996	0.70819998	0.19570000
1	1.21659994	0.01620000	1.79089999
1	2.07579994	-2.19540000	0.99570000
1	2.29340005	-1.54840004	-0.62169999
6	6.19080019	-1.42859995	0.81150001
1	4.75209999	-2.19930005	-0.50749999
1	4.59040022	-2.79040003	1.12750006
8	7.04150009	-2.49020004	0.80440003
8	6.55179977	-0.28600001	0.99540001
6	8.44009972	-2.21339989	0.94870001
6	8.92039967	-1.33459997	-0.20819999
1	8.90299988	-3.19039989	0.76840001
6	8.82499981	-1.74230003	2.34549999
8	10.11260033	-0.78020000	0.08800000
8	8.35369968	-1.22329998	-1.27160001
6	10.70790005	0.02990000	-0.93550003

1 -8.69649982	-2.35910010	1.60380006		6 11.59630013	1.04009998	-0.22139999	
6 2.31529999	-1.27590001	0.44319999		1 9.91959953	0.56339997	-1.47220004	
6 3.72550011	-0.81209999	0.81180000		6 11.51459980	-0.82510000	-1.90840006	
6 4.77820015	-1.88279998	0.54329997		1 9.91199970	-1.72749996	2.44449997	
1 3.75810003	-0.52719998	1.86989999		1 8.41740036	-2.44250011	3.07990003	
1 3.98099995	0.09160000	0.24860001		1 8.44169998	-0.74330002	2.55010009	
8 -2.75489998	-0.87459999	-1.90980005		8 11.90489960	2.04220009	-1.06900001	
6 -3.45199990	-1.88950002	-1.98469996		8 11.99769974	0.95859998	0.91589999	
6 -3.51729989	-2.69910002	-3.25209999		6 12.78730011	3.04430008	-0.53659999	
8 -4.11759996	-2.25629997	-0.90429997		1 12.93649960	3.75950003	-1.34410000	
6 -3.03349996	-4.15140009	-3.09349990		1 12.33160019	3.53080010	0.32800001	
1 -4.54790020	-2.68429995	-3.62809992		1 13.73779964	2.59680009	-0.23860000	
1 -2.90170002	-2.16409993	-3.97639990		1 11.98849964	-0.19000000	-2.66050005	
6 -5.09579992	-3.32879996	-0.90270001		1 12.28880024	-1.38450003	-1.37629998	
1 -2.08890009	-4.16179991	-2.53690004		1 10.84910011	-1.52590001	-2.41650009	

Data for structures presented in Scheme 6

I-2'_Mg

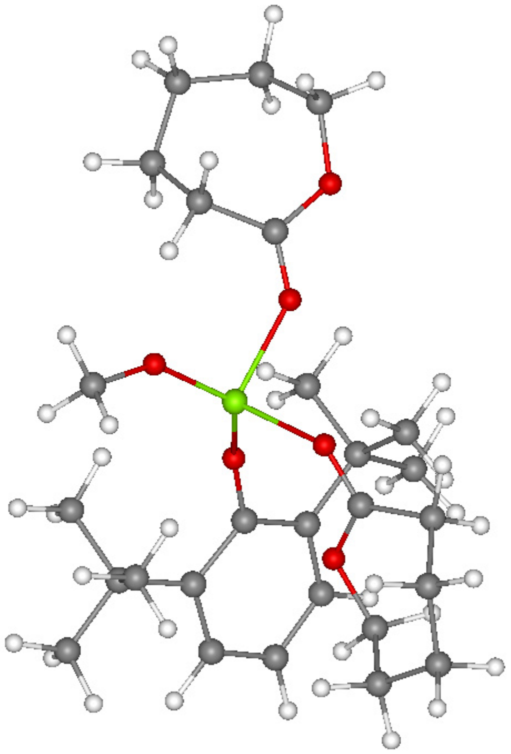

Zero-point vibrational energy	1784683.0 (Joules/Mol)
	426.54948 (Kcal/Mol)
Zero-point correction=	0.679750 (Hartree/Particle)
Thermal correction to Energy=	0.719103
Thermal correction to Enthalpy=	0.720047
Thermal correction to Gibbs Free Energy=	0.604580
Sum of electronic and zero-point Energies=	-1705.797246
Sum of electronic and thermal Energies=	-1705.757893
Sum of electronic and thermal Enthalpies=	-1705.756949
Sum of electronic and thermal Free Energies=	-1705.872416

cartesian

8	-1.63320374	0.75279218	-2.54900122	1	-0.22190370	0.54409218	1.85919869
6	-1.40160370	0.82269216	-3.91410136	8	0.18789630	2.31889224	-0.29470125
1	-1.03700376	-0.13080782	-4.33640146	6	1.31929624	2.47839212	0.17269874
1	-0.65780371	1.59449220	-4.18610144	8	2.16109633	1.46659219	0.10809873
12	-0.80780369	0.56899220	-0.88910127	6	1.73529625	3.79649210	0.77209872
8	0.13459630	-0.89550781	-0.14480126	6	3.46119618	1.46709216	0.74919873
6	1.22249627	-1.51050782	0.28239873	6	2.92789626	4.46169233	0.06189874
6	2.20499635	-1.99840784	-0.64440125	6	4.46029615	2.38929224	0.07159874
6	3.39919639	-2.52220774	-0.13730127	1	3.76279640	0.42199215	0.67419875
1	4.16589642	-2.87290788	-0.82050127	1	2.92339635	5.52069235	0.34079874
6	3.64339638	-2.62950778	1.22909880	1	2.76799631	4.43219233	-1.02230132
6	2.64119625	-2.25680780	2.12049866	1	1.96859622	3.64189219	1.83319879

1	2.82149625	-2.40140772	3.18099880	1	0.85299629	4.43609238	0.72429872
6	1.42389631	-1.72120786	1.68899870	6	4.29299641	3.86909223	0.41459873
6	1.96959639	-1.98390794	-2.16870117	1	3.33159637	1.70519221	1.81019878
6	0.32569629	-1.39340782	2.71959877	1	4.40389633	2.22809219	-1.01120126
6	2.00169635	-0.54830784	-2.72600126	1	5.45969629	2.06109214	0.38019875
1	1.22779632	0.08459218	-2.29080129	1	4.47169638	4.00689220	1.48959875
1	1.84669626	-0.55240786	-3.81130123	1	5.07019615	4.44599199	-0.09900127
1	2.97029638	-0.07790782	-2.52240133	8	-2.44980359	0.88429219	0.33769873
6	0.62799633	-2.66250777	-2.51400137	6	-3.67240381	0.92199218	0.20779873
1	-0.21590370	-2.15730786	-2.04460120	6	-4.36730385	0.67469215	-1.10120130
1	0.63289630	-3.70440793	-2.17460132	8	-4.36890364	1.18019211	1.31499875
1	0.47189629	-2.66190791	-3.59970117	6	-5.16870356	-0.64060783	-1.11420131
6	3.05809641	-2.76440787	-2.93000126	1	-5.03230381	1.51969218	-1.32210124
1	3.12749624	-3.80500793	-2.59570122	1	-3.56980371	0.66189218	-1.85710120
1	4.04889631	-2.30600786	-2.83710122	6	-5.81350374	1.21099222	1.31009877
1	2.80979633	-2.77670789	-3.99730134	1	-4.55880356	-1.44370782	-0.68430126
6	0.69249630	-1.87250781	4.13579845	1	-5.32900381	-0.91320783	-2.16200137
1	1.56999624	-1.35650778	4.54119873	1	-6.16040373	1.96529222	0.59519875
1	0.88229632	-2.95060778	4.16879845	1	-6.05050373	1.56709218	2.31359863
1	-0.14370370	-1.66490781	4.81319857	6	-6.52230358	-0.56210786	-0.40940127
6	-0.98840368	-2.10860777	2.34219885	1	-7.16130352	0.15109217	-0.94790125
1	-1.32730365	-1.81810784	1.34779871	1	-7.02310371	-1.53420782	-0.48150125
1	-1.77440369	-1.86410785	3.06779885	6	-6.45480347	-0.14640781	1.06049871
1	-0.84570372	-3.19510794	2.35229874	1	-5.91880369	-0.89490783	1.65619874
6	0.09229630	0.12619218	2.81439877	1	-7.47280359	-0.10070782	1.46559870
1	1.01079631	0.63259214	3.13669872	1	-2.32560372	1.07689226	-4.46610165
1	-0.69010371	0.35279217	3.54929876	1	4.58229637	-3.04120779	1.59229875

I-2'_Zn

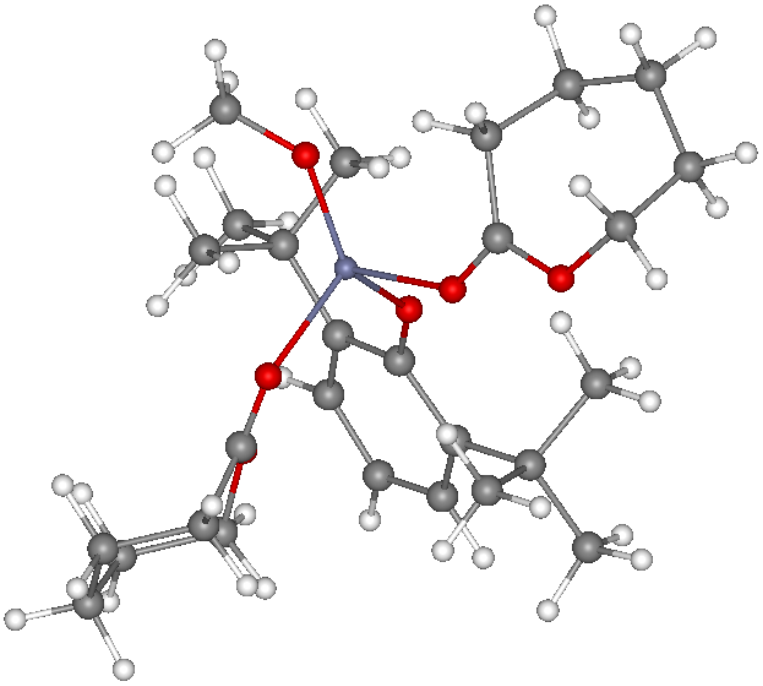

Zero-point vibrational energy	1783329.2 (Joules/Mol)
	426.22590 (Kcal/Mol)
Zero-point correction=	0.679234 (Hartree/Particle)
Thermal correction to Energy=	0.718836
Thermal correction to Enthalpy=	0.719781
Thermal correction to Gibbs Free Energy=	0.603588
Sum of electronic and zero-point Energies=	-3284.754707
Sum of electronic and thermal Energies=	-3284.715105
Sum of electronic and thermal Enthalpies=	-3284.714161
Sum of electronic and thermal Free Energies=	-3284.830353

cartesian

8	-1.64441121	1.01127183	-2.59066296	8	0.24238871	2.38187194	-0.28546283
6	-1.08541131	1.18947184	-3.85936284	6	1.35708869	2.44447184	0.23803715
1	-0.69671124	0.25337183	-4.29436302	8	2.15158868	1.39507174	0.13423716
1	-0.25871128	1.92117178	-3.87256289	6	1.80978870	3.69367194	0.94893712
8	-0.04091127	-0.94942814	-0.18866284	6	3.42598867	1.29267180	0.81773716
6	1.09318876	-1.52952826	0.19113715	6	3.05418873	4.35267210	0.32913718
6	2.06688881	-1.97652829	-0.76076281	6	4.48928881	2.21377182	0.24343716
6	3.26138878	-2.52772808	-0.28036284	1	3.68718863	0.24337181	0.67823720
1	4.02128887	-2.85832810	-0.98036283	1	3.08648872	5.38827181	0.68403715
6	3.51198864	-2.69062805	1.07833707	1	2.93288875	4.40797186	-0.75926280
6	2.52228880	-2.33842802	1.99183702	1	1.99788880	3.45117188	2.00243711
1	2.71038866	-2.51962805	3.04503703	1	0.95838869	4.37487173	0.92043716
6	1.30718875	-1.77772820	1.58863711	6	4.37688875	3.67307186	0.68413717
6	1.84148872	-1.88472831	-2.28416300	1	3.26778865	1.45877182	1.88833714
6	0.22318871	-1.46542823	2.63893723	1	4.46668863	2.13217187	-0.84966284

6	1.95978880	-0.42632821	-2.76216292	1	5.46088886	1.81827176	0.56183720
1	1.23568869	0.23437181	-2.27916288	1	4.52168894	3.72807193	1.77173710
1	1.79528868	-0.35592818	-3.84336281	1	5.19838858	4.24557209	0.23893715
1	2.95658875	-0.02982818	-2.54086280	8	-2.37591124	0.99547184	0.32963717
6	0.47358876	-2.47742820	-2.68246293	6	-3.59671116	0.99357182	0.20363717
1	-0.35821128	-1.94842827	-2.21856284	6	-4.30211115	0.69667184	-1.09066284
1	0.41378874	-3.52852821	-2.37916279	8	-4.29911137	1.25807178	1.31183708
1	0.34938872	-2.43352818	-3.77126288	6	-5.05841112	-0.64462817	-1.06766284
6	2.89768863	-2.68182802	-3.07456279	1	-5.00061131	1.51287174	-1.31556284
1	2.92008877	-3.73622799	-2.77956295	1	-3.52641129	0.70557183	-1.86476290
1	3.90748882	-2.27172804	-2.96516299	6	-5.74181128	1.24467182	1.31543708
1	2.65168881	-2.64232802	-4.14156294	1	-4.42001104	-1.41752827	-0.62376285
6	0.61538875	-1.95462811	4.04463720	1	-5.21891117	-0.94722813	-2.10736299
1	1.50378871	-1.44572818	4.43543720	1	-6.11891127	1.97027183	0.58573717
1	0.79798871	-3.03422809	4.07033730	1	-5.98551130	1.61707175	2.31163716
1	-0.20621127	-1.74472821	4.73873711	6	-6.40891123	-0.59222817	-0.35436282
6	-1.09451127	-2.18242812	2.27603722	1	-7.07381105	0.08657182	-0.90586281
1	-1.45891130	-1.86862826	1.29803717	1	-6.87901115	-1.58132827	-0.39826283
1	-1.86261129	-1.96032822	3.02763700	6	-6.34471130	-0.13712817	1.10393715
1	-0.94511127	-3.26802802	2.25753713	1	-5.78221130	-0.85412818	1.71393716
6	-0.01461127	0.05247182	2.74613714	1	-7.36131144	-0.11232818	1.51463711
1	0.90518868	0.55947185	3.06483722	1	-1.85451126	1.56927180	-4.55156279
1	-0.78861123	0.26897183	3.49263716	1	4.44898891	-3.12582803	1.41833711
1	-0.34511128	0.47577181	1.79913712	30	-0.72821128	0.60907185	-1.01216280

I-3_Mg

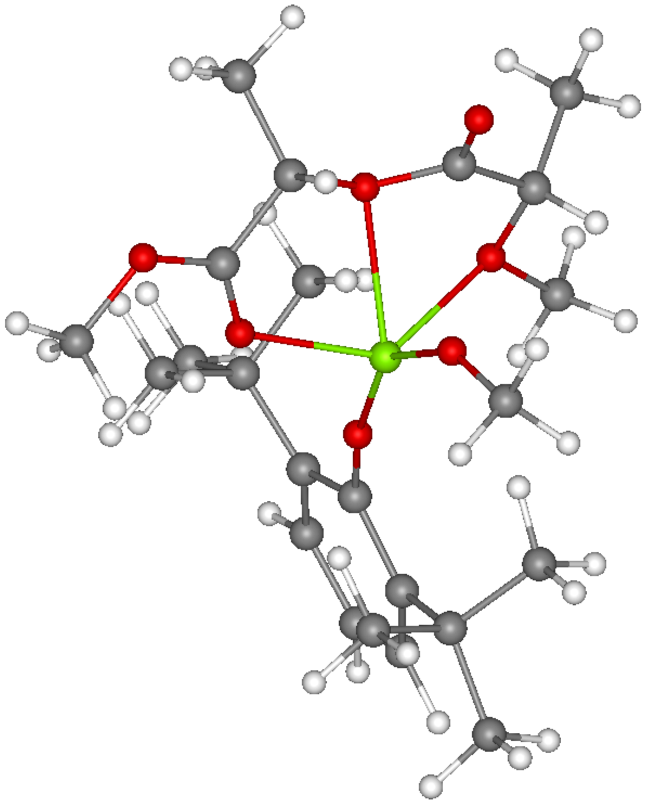

Zero-point vibrational energy	1543097.8 (Joules/Mol)
	368.80923 (Kcal/Mol)
Zero-point correction=	0.587735 (Hartree/Particle)
Thermal correction to Energy=	0.625726
Thermal correction to Enthalpy=	0.626670
Thermal correction to Gibbs Free Energy=	0.516054
Sum of electronic and zero-point Energies=	-1625.072400
Sum of electronic and thermal Energies=	-1625.034410
Sum of electronic and thermal Enthalpies=	-1625.033465
Sum of electronic and thermal Free Energies=	-1625.144081

cartesian

8	0.86555380	0.04294493	0.16889279	1	0.11495383	1.51364493	-1.57450724
6	2.17635393	0.21564494	0.05699278	12	-0.91024619	-0.53215510	0.24549279
6	3.08435392	-0.82875508	0.42779279	8	-2.04794621	-1.96925509	0.62599277
6	4.45195341	-0.62975508	0.21229279	6	-2.18274617	-3.25415492	1.13329279
1	5.15815353	-1.41235507	0.46889278	1	-1.46084619	-3.96795487	0.69839275
6	4.95265341	0.55244493	-0.32140723	1	-3.19054604	-3.65665483	0.93089277
6	4.07065344	1.58584487	-0.61830723	1	6.02075386	0.67504495	-0.48410723
1	4.48205376	2.51314497	-1.00270724	8	-1.23294616	-0.73145509	-1.90290725
6	2.69075394	1.46154487	-0.43050721	6	-2.90034604	1.05194497	1.73959279
6	2.59765387	-2.13795495	1.07999277	6	-3.87514615	0.69934493	0.63199276
6	1.76875377	2.66454506	-0.71140724	8	-3.05414605	0.28544492	-0.47040722
6	1.71805382	-2.95225501	0.11119278	6	-3.51824617	-0.79645509	-1.17880726
1	0.81465381	-2.41465497	-0.18100722	6	-2.52524614	-1.24605513	-2.23740721

1	1.40455389	-3.89485502	0.57509273	8	-3.48944616	1.60744488	2.78269267
1	2.27525377	-3.19245505	-0.80130726	6	-2.63254619	1.92314494	3.90569282
6	1.82875395	-1.82915509	2.38159275	1	-2.16074610	1.01364493	4.27919292
1	0.98975378	-1.15225506	2.21219277	8	-4.63544655	-1.23675501	-1.07550728
1	2.49395394	-1.35125506	3.10899282	8	-1.69574618	0.83564490	1.68219280
1	1.44255388	-2.75255489	2.82949281	6	-4.79304647	1.84434497	0.22899279
6	3.76365399	-3.06195498	1.47709274	6	-3.00604606	-0.79405504	-3.61520720
1	4.44895363	-2.58135486	2.18279266	1	-1.86824620	2.63864493	3.60089278
1	4.34205341	-3.40005493	0.61059278	1	-3.29384613	2.35424495	4.65409279
1	3.36305380	-3.95595503	1.96789277	6	-0.16694617	-1.19245505	-2.74610734
6	2.55225396	3.90954494	-1.16320729	1	-0.23844619	-0.73755509	-3.73760724
1	3.08295393	3.74724507	-2.10770726	1	-0.19034618	-2.28495502	-2.82830715
1	3.27845383	4.23824501	-0.41250721	1	0.75925380	-0.87815505	-2.26720715
1	1.85155392	4.73704481	-1.32290721	1	-5.47024632	1.49904490	-0.55430722
6	1.01675391	3.05594516	0.57739276	1	-4.21604633	2.69304514	-0.14650722
1	0.43275380	2.22194505	0.96829277	1	-5.38754654	2.16984510	1.08489275
1	0.34085381	3.89954495	0.38399279	1	-4.03654623	-1.12505507	-3.76290727
1	1.72675383	3.36554503	1.35249281	1	-2.39624619	-1.23435509	-4.40710688
6	0.76355380	2.34934497	-1.83610725	1	-2.96494603	0.29514492	-3.70260715
1	1.29075384	2.09594512	-2.76310730	1	-2.50704622	-2.33945489	-2.17440724
1	0.13115381	3.22314501	-2.04010725	1	-4.45754623	-0.16315506	0.97169274
				1	-2.04144621	-3.29155493	2.22829270

I-3_Zn

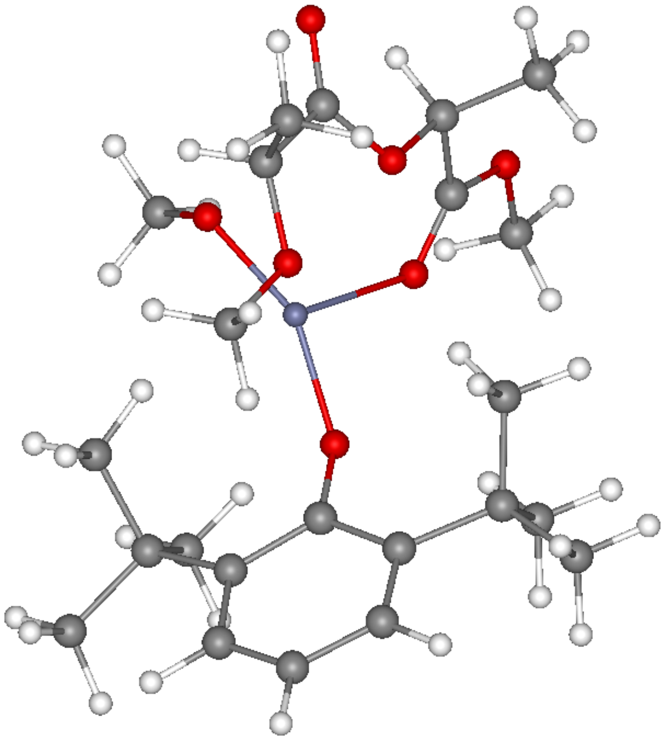

Zero-point vibrational energy	1540690.4 (Joules/Mol)
	368.23384 (Kcal/Mol)
Zero-point correction=	0.586818 (Hartree/Particle)
Thermal correction to Energy=	0.625189
Thermal correction to Enthalpy=	0.626133
Thermal correction to Gibbs Free Energy=	0.514591
Sum of electronic and zero-point Energies=	-3204.032040
Sum of electronic and thermal Energies=	-3203.993669
Sum of electronic and thermal Enthalpies=	-3203.992725
Sum of electronic and thermal Free Energies=	-3204.104267

cartesian

8	-0.81183052	0.21369863	-0.79138547	1	0.54246950	1.67589867	0.64201450
6	-2.01143050	0.37649861	-0.21028548	30	0.71256948	-0.76590139	-0.35738549
6	-3.02393055	-0.62760139	-0.31428549	8	1.73526955	-2.32240152	-0.39578548
6	-4.22303057	-0.42350137	0.38021451	1	0.95166945	-3.56010151	-1.89078546
1	-5.00193071	-1.17750132	0.34431452	1	2.58896947	-3.93750143	-1.32818544
6	-4.46583080	0.73219860	1.11311448	1	-5.40443087	0.85699862	1.64761448
6	-3.51563048	1.74839866	1.11251450	8	0.84496951	-0.81290138	1.93161452
1	-3.74773049	2.66729879	1.64011455	6	3.20066953	0.71689862	-1.49538553
6	-2.29403043	1.61629868	0.44521451	6	3.92616963	0.43209863	-0.19098550
6	-2.85483050	-1.89850140	-1.17528546	8	2.91286945	0.04219863	0.74151450
6	-1.31593049	2.80769873	0.38381451	6	3.21566963	-1.00520134	1.55391455
6	-1.89373040	-2.89920139	-0.50708550	6	2.03546953	-1.45090139	2.40171456
1	-0.87463045	-2.51730156	-0.39998549	8	4.01886940	1.21659863	-2.41368556
1	-1.82183051	-3.81840134	-1.10088551	6	3.43226957	1.48019862	-3.70768547
1	-2.25333047	-3.17400122	0.49061453	1	3.04896951	0.55449861	-4.13918591
6	-2.36133051	-1.54280138	-2.59288549	8	4.31806946	-1.48890138	1.64991450
1	-1.40253043	-1.02510142	-2.57568550	8	2.01546955	0.50349861	-1.69928551
1	-3.08443046	-0.89020139	-3.09438539	6	4.71926928	1.61879861	0.34041452
1	-2.25953054	-2.45500135	-3.19348550	6	2.32866955	-1.15990138	3.87301445
6	-4.19043064	-2.64490128	-1.36088550	1	2.62216949	2.20409870	-3.61128545
1	-4.96263075	-2.00100136	-1.79438543	1	4.24326944	1.88209867	-4.31138563
1	-4.57543087	-3.06330156	-0.42498550	6	-0.35243049	-1.26400137	2.57771444
1	-4.03803062	-3.48600149	-2.04628539	1	-0.37643048	-0.93510139	3.62051463
6	-1.90123057	4.06789827	1.04551446	1	-0.43073049	-2.35610151	2.52611446
1	-2.07663035	3.93379831	2.11861444	1	-1.18703043	-0.81020141	2.04361463
1	-2.84103036	4.38049841	0.57861453	1	5.22026920	1.32539856	1.26511455
1	-1.19083047	4.89479828	0.93481451	1	4.06046963	2.46579885	0.54621452
6	-1.02633047	3.16719866	-1.08898544	1	5.47606945	1.92379868	-0.38468549
1	-0.57953048	2.32639885	-1.62138546	1	3.31096959	-1.56320143	4.12841415
1	-0.34093049	4.02299833	-1.14068544	1	1.59236956	-1.63540137	4.52431440
1	-1.95173049	3.44889879	-1.60358548	1	2.32536960	-0.08290138	4.06351423
6	0.00316951	2.49679852	1.11391449	1	1.95666957	-2.53020144	2.22681451

1	-0.17973050	2.22719860	2.15971446	1	4.59196949	-0.41990137	-0.36358547
1	0.65706950	3.37839842	1.10471451	1	2.31806946	-2.55090141	-2.39908552
6	1.89676952	-3.10990143	-1.54468548				

TS-1_Mg

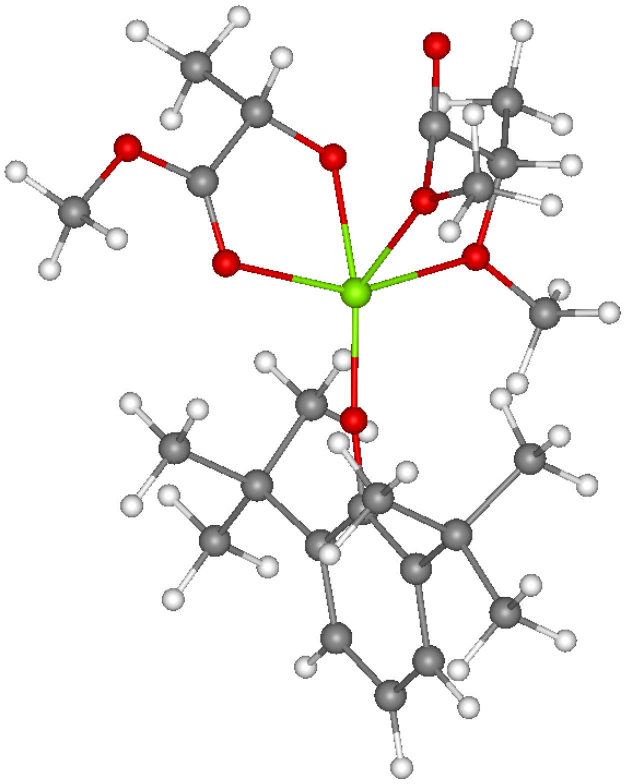

Zero-point vibrational energy	1542598.4 (Joules/Mol)
	368.68986 (Kcal/Mol)
Zero-point correction=	0.587545 (Hartree/Particle)
Thermal correction to Energy=	0.624118
Thermal correction to Enthalpy=	0.625062
Thermal correction to Gibbs Free Energy=	0.518925
Sum of electronic and zero-point Energies=	-1625.073658
Sum of electronic and thermal Energies=	-1625.037085
Sum of electronic and thermal Enthalpies=	-1625.036141
Sum of electronic and thermal Free Energies=	-1625.142278

cartesian

8	-0.87167847	0.06515653	-0.24615948	12	0.96152151	-0.13884348	-0.07875948
6	-2.18987870	0.16995652	-0.13215949	8	2.29462147	-1.70444357	-0.34525949
6	-3.03277874	-0.89954346	-0.57345951	6	2.54172134	-3.01074338	-0.85945952
6	-4.41247845	-0.78264344	-0.37955949	1	2.12632132	-3.78064346	-0.19915947
1	-5.07207870	-1.58504355	-0.69265950	1	3.61852145	-3.15874338	-0.97015947
6	-4.98347855	0.34315655	0.20414053	1	-6.05967855	0.40365654	0.34714052
6	-4.16197872	1.40045643	0.57984048	8	1.11272144	-0.95384347	1.89734054
1	-4.62717867	2.28265667	1.00674045	6	2.91802144	1.19065642	-1.66315949
6	-2.77377868	1.35605645	0.41724050	6	3.70832133	0.96365654	-0.38425949

6	-2.45787859	-2.14554334	-1.27415955	8	2.85152125	0.38695654	0.56054050
6	-1.91697848	2.57895660	0.79874051	6	3.21192145	-1.27884352	0.79014051
6	-1.53907847	-2.93924332	-0.32405949	6	2.43462133	-1.49454355	2.09884071
1	-0.69177854	-2.34164333	0.01544052	8	3.60302138	1.73975646	-2.64965940
1	-1.14827847	-3.83334351	-0.82595950	6	2.88632131	1.96895647	-3.88315940
1	-2.09467864	-3.26744342	0.56154048	1	2.52832127	1.02165651	-4.28875923
6	-1.68677855	-1.73194349	-2.54475927	8	4.39802122	-1.56394351	0.68024051
1	-0.88827848	-1.02264357	-2.32115936	8	1.72602153	0.89005655	-1.76895952
1	-2.36317873	-1.25404358	-3.26165938	6	4.32302141	2.26045656	0.14484052
1	-1.24987853	-2.61334348	-3.03145933	6	3.14382148	-0.89144343	3.29594064
6	-3.55857873	-3.12164330	-1.72675943	1	2.04162145	2.63635659	-3.70775938
1	-4.25977850	-2.65694332	-2.42745948	1	3.61062145	2.42735648	-4.55325937
1	-4.12997866	-3.52444339	-0.88375950	6	0.10792145	-1.40744352	2.80424070
1	-3.09807873	-3.97214341	-2.24245930	1	0.29672146	-1.02544355	3.81224060
6	-2.76997852	3.76145649	1.29234052	1	0.07542145	-2.50304341	2.82184052
1	-3.32307863	3.52435660	2.20744061	1	-0.84327853	-1.02154350	2.43884063
1	-3.48517871	4.09855652	0.53484052	1	4.89242125	2.02705669	1.04654050
1	-2.11327863	4.60855627	1.52054048	1	3.54372144	2.98255658	0.40454051
6	-1.13947856	3.08645654	-0.43375948	1	4.99512148	2.70905662	-0.59055948
1	-0.51967859	2.30545664	-0.87645948	1	4.16462135	-1.27834356	3.33424067
1	-0.49647853	3.93225670	-0.15835948	1	2.64232135	-1.15494347	4.23144054
1	-1.83617842	3.43225670	-1.20525944	1	3.18252134	0.19545652	3.20174074
6	-0.94677854	2.23965669	1.94734061	1	2.33862138	-2.58324337	2.21764064
1	-1.50247848	1.94365644	2.84394073	1	4.51782131	0.25305653	-0.61915952
1	-0.33257857	3.11115670	2.20584059	1	2.05402136	-3.07374334	-1.83365953
1	-0.27527854	1.41845644	1.69234049				

TS-1_Zn

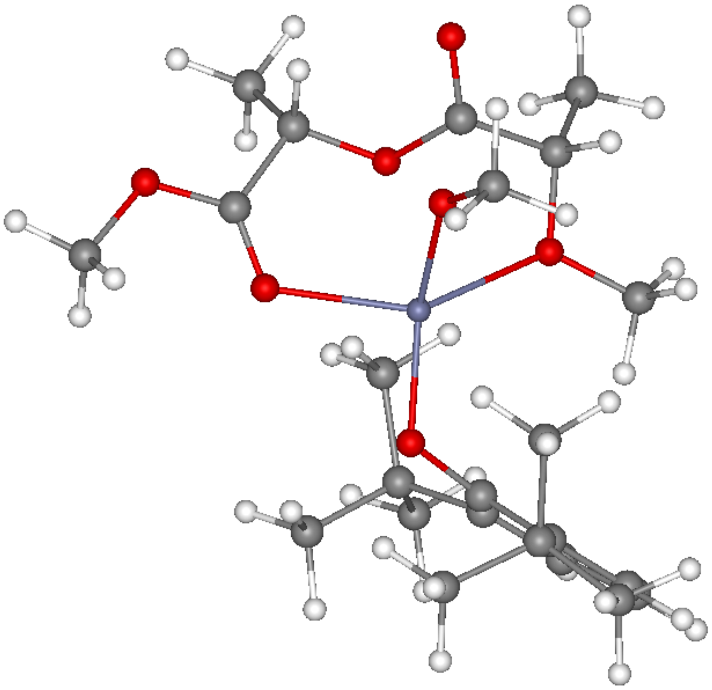

Zero-point vibrational energy	1541839.0 (Joules/Mol)
	368.50837 (Kcal/Mol)
Zero-point correction=	0.587255 (Hartree/Particle)
Thermal correction to Energy=	0.623939
Thermal correction to Enthalpy=	0.624883
Thermal correction to Gibbs Free Energy=	0.519254
Sum of electronic and zero-point Energies=	-3204.025503
Sum of electronic and thermal Energies=	-3203.988820
Sum of electronic and thermal Enthalpies=	-3203.987876
Sum of electronic and thermal Free Energies=	-3204.093505

cartesian

8	-0.80293465	0.37481734	-1.00353909	30	0.79246533	-0.36968267	-0.35783911
6	-1.95393455	0.38091734	-0.30303910	8	2.03156519	-1.90028262	-0.37663910
6	-2.90433478	-0.66768265	-0.48873910	6	1.97946537	-3.28898287	-0.62533909
6	-4.02763462	-0.69138265	0.34566090	1	1.14036536	-3.77678275	-0.11083909
1	-4.75283432	-1.49348259	0.25676090	1	2.92166519	-3.73748279	-0.28823909
6	-4.25633478	0.29831734	1.29676092	1	-5.12883472	0.24691734	1.94356096
6	-3.38833475	1.38341737	1.37256086	8	0.58776534	-1.00218272	1.76146090
1	-3.62133479	2.17701721	2.07446098	6	3.27516532	0.83851737	-1.40983903
6	-2.24683475	1.47361732	0.56796092	6	3.84486532	0.46501735	-0.05323909
6	-2.74673486	-1.71298265	-1.61023915	8	2.78366518	0.00191734	0.76206088
6	-1.38883471	2.75481725	0.57976091	6	2.87236524	-1.43748260	1.09386086
6	-1.49993467	-2.59478283	-1.41183913	6	1.79636538	-1.67738259	2.16116095
1	-0.57883465	-2.02888274	-1.56683910	8	4.19096518	1.31081736	-2.24293900
1	-1.49443471	-3.41568279	-2.13893890	6	3.72766519	1.68071735	-3.55943894
1	-1.47983468	-3.03738284	-0.40983909	1	3.30656528	0.81101733	-4.06583929

6	-2.65353489	-0.99098265	-2.97083902	8	3.96896529	-1.98198259	1.16886091
1	-1.80693471	-0.30248266	-2.99453902	8	2.09226537	0.72981739	-1.72153914
1	-3.56763482	-0.41978267	-3.16533899	6	4.55396557	1.63641739	0.62226093
1	-2.53393483	-1.72148263	-3.78073907	6	2.28886533	-1.21338260	3.52456093
6	-3.95293498	-2.66578269	-1.68133914	1	2.97216511	2.46411729	-3.48553896
1	-4.89373446	-2.12908268	-1.84023905	1	4.61126566	2.04201722	-4.08183908
1	-4.05423450	-3.28068280	-0.78003907	6	-0.58893466	-1.41618264	2.46226096
1	-3.82223487	-3.34978271	-2.52703905	1	-0.51453471	-1.16028261	3.52346110
6	-1.97503459	3.82741714	1.51506090	1	-0.73853469	-2.49728274	2.35376096
1	-1.96673477	3.51611733	2.56566095	1	-1.43123472	-0.88328266	2.02326107
1	-3.00043488	4.09741735	1.24286091	1	4.96456528	1.29061735	1.57296085
1	-1.36953461	4.73731709	1.44166088	1	3.85576510	2.45401716	0.82006091
6	-1.36063468	3.35511732	-0.84173912	1	5.37106562	2.00701714	-0.00033909
1	-0.94503462	2.64561725	-1.55963910	1	3.24946523	-1.68918264	3.73436093
1	-0.74993467	4.26671743	-0.85623908	1	1.59196532	-1.49048269	4.32006073
1	-2.37193489	3.62301731	-1.16593909	1	2.42166519	-0.12878266	3.53086114
6	0.05446531	2.49811721	1.05466092	1	1.60836530	-2.75818276	2.17156100
1	0.08126530	1.92891741	1.98906100	1	4.55396557	-0.35928267	-0.20453909
1	0.56896532	3.45131731	1.22796094	1	1.87346530	-3.46128273	-1.70163906
1	0.63626528	1.96321738	0.30106091				

I-4_Mg

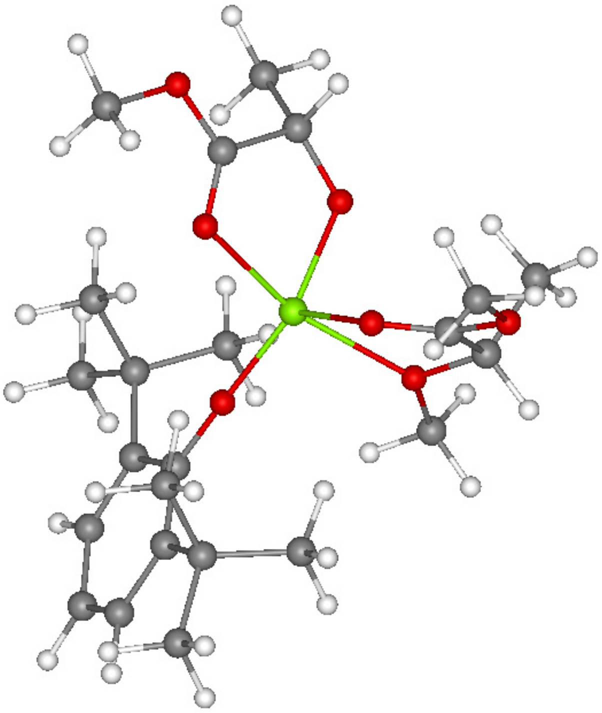

Zero-point vibrational energy	1544020.4 (Joules/Mol)
	369.02973 (Kcal/Mol)
Zero-point correction=	0.588086 (Hartree/Particle)
Thermal correction to Energy=	0.625788

Thermal correction to Enthalpy=	0.626733
Thermal correction to Gibbs Free Energy=	0.517743
Sum of electronic and zero-point Energies=	-1625.096261
Sum of electronic and thermal Energies=	-1625.058558
Sum of electronic and thermal Enthalpies=	-1625.057614
Sum of electronic and thermal Free Energies=	-1625.166604

cartesian

8	-0.80579251	-0.06235361	-0.09595655	12	1.05170751	0.15574639	0.07354344
6	-2.12369251	-0.19005361	-0.12445655	8	4.00430727	-2.80575371	0.33804345
6	-2.72349238	-1.35965359	-0.69845653	6	4.53620720	-2.95835376	-0.99665654
6	-4.11299276	-1.49895358	-0.63715655	1	4.87110710	-1.99245358	-1.37715662
1	-4.58539248	-2.38345361	-1.05165660	1	3.77360749	-3.37135363	-1.65805662
6	-4.92869282	-0.53125358	-0.06065655	1	-6.00639248	-0.66985357	-0.02045655
6	-4.34679270	0.62914640	0.43814346	8	1.21130741	-1.23465359	1.78224337
1	-5.00039291	1.38854635	0.85394347	6	2.40660763	2.15144634	-1.36515665
6	-2.96499252	0.84134638	0.40954345	6	2.92560744	2.29904628	0.06644346
6	-1.87709248	-2.44585371	-1.38905656	8	2.38090754	1.32364643	0.85944343
6	-2.38869238	2.18134618	0.90714347	6	2.95540762	-2.00875354	0.46054345
6	-0.94089252	-3.13015366	-0.37415653	6	2.47050762	-1.88045359	1.89454341
1	-0.28069252	-2.40835381	0.10624346	8	2.90440750	2.99814630	-2.25895643
1	-0.32519251	-3.89095378	-0.87225652	6	2.41270757	2.86704636	-3.60745645
1	-1.52579260	-3.62925363	0.40724346	1	2.64830756	1.87694645	-4.00195646
6	-1.06219256	-1.83195364	-2.54605651	8	2.42490745	-1.43455362	-0.48255655
1	-0.42389250	-1.01525366	-2.20745659	8	1.58250749	1.28484643	-1.67205656
1	-1.73459256	-1.43195355	-3.31305647	6	2.64980745	3.72184634	0.58344346
1	-0.43069249	-2.59595370	-3.01745653	6	3.46230745	-1.10615361	2.76204348
6	-2.74019241	-3.55975366	-2.00785661	1	1.33260739	3.01974630	-3.63265657
1	-3.43609238	-3.17455363	-2.76045656	1	2.92440748	3.64034629	-4.17785645
1	-3.31649256	-4.10835361	-1.25505662	6	0.32110748	-1.40915358	2.88664341
1	-2.08709240	-4.28495359	-2.50745654	1	0.67610747	-0.86515361	3.76734352
6	-3.48779249	3.15814638	1.36364341	1	0.21430749	-2.47495365	3.11834359
1	-4.05129290	2.77854633	2.22284341	1	-0.63979250	-1.01015365	2.56594348
1	-4.19639254	3.38994622	0.56164348	1	3.05030751	3.79534626	1.59764338
1	-3.02419233	4.10234642	1.67144334	1	1.57200742	3.90144634	0.63374346
6	-1.63219261	2.87974620	-0.24165654	1	3.11580753	4.49144650	-0.03985655
1	-0.84819251	2.24354625	-0.65425652	1	4.44260740	-1.58955359	2.74114347
1	-1.17839253	3.81504631	0.11004345	1	3.11510754	-1.09275365	3.79834342
1	-2.32349253	3.12584639	-1.05525661	1	3.53210759	-0.07975361	2.39094353
6	-1.46039259	1.98284638	2.12114358	1	2.32660747	-2.89895368	2.28164363
1	-2.00109243	1.49224639	2.93874359	1	4.02720737	2.19664621	-0.02925655
1	-1.09879255	2.95024633	2.49064350	1	5.37350750	-3.64525366	-0.89325655
1	-0.58579248	1.37844646	1.87874341				

I-4_Zn

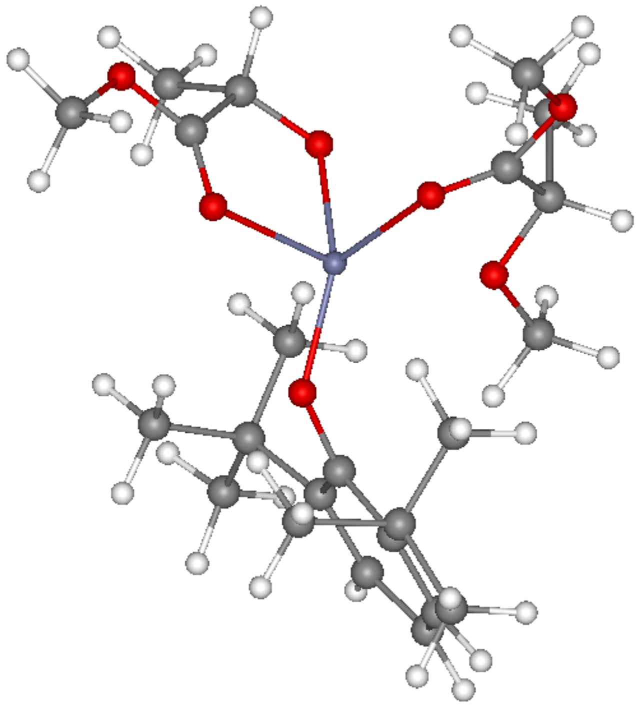

Zero-point vibrational energy	1541504.4 (Joules/Mol)
	368.42840 (Kcal/Mol)
Zero-point correction=	0.587128 (Hartree/Particle)
Thermal correction to Energy=	0.625382
Thermal correction to Enthalpy=	0.626326
Thermal correction to Gibbs Free Energy=	0.515230
Sum of electronic and zero-point Energies=	-3204.052312
Sum of electronic and thermal Energies=	-3204.014059
Sum of electronic and thermal Enthalpies=	-3204.013114
Sum of electronic and thermal Free Energies=	-3204.124210

cartesian

8	0.71273017	-0.33367103	-0.81637537	30	-0.97356975	-0.15227102	-0.00997535
6	1.96583021	-0.14577101	-0.38197535	8	-3.24877000	3.31842899	1.04402471
6	2.69113016	1.01022899	-0.81677538	6	-4.07956982	3.60832882	-0.09897535
6	3.95343018	1.24792898	-0.26487535	1	-4.64537001	2.72072887	-0.38647535
1	4.51063013	2.13332891	-0.55197537	1	-3.46376991	3.93872881	-0.93677539
6	4.53822994	0.36942896	0.64222461	1	5.51693010	0.58382899	1.06502473
6	3.88183022	-0.81647098	0.95092463	8	-0.43076980	1.32042897	1.73402464
1	4.38493013	-1.52387094	1.60142469	6	-3.12626982	-1.56437099	-1.18077528
6	2.61593008	-1.12417102	0.43662465	6	-3.26916981	-1.75137103	0.32802466
6	2.14163017	1.95012903	-1.90827537	8	-2.35626984	-0.98217094	1.01732469
6	1.99513018	-2.50517106	0.74052465	6	-2.33746982	2.36552882	0.87562460
6	0.88523018	2.69922900	-1.43217528	6	-1.53046978	2.11672878	2.13802457

1	0.06733021	2.01142883	-1.22527528	8	-4.07756996	-2.14907122	-1.90627527
1	0.54643023	3.40132880	-2.20467544	6	-3.95566988	-2.01737118	-3.33547544
1	1.09443021	3.27382898	-0.52237540	1	-3.99056983	-0.96517098	-3.62387538
6	1.81033027	1.13432896	-3.17607546	8	-2.17466974	1.76932895	-0.17877534
1	1.06483018	0.36522898	-2.96967554	8	-2.20826983	-0.92707098	-1.69267535
1	2.71163011	0.64632899	-3.56337547	6	-3.15397000	-3.24797106	0.66842461
1	1.42283022	1.79662895	-3.96057534	6	-2.38466978	1.46082902	3.22462463
6	3.16782999	3.02102900	-2.32087541	1	-3.01646996	-2.45547104	-3.67757535
1	4.10183001	2.58162880	-2.68667555	1	-4.80536985	-2.55817103	-3.74917555
1	3.40773010	3.70972896	-1.50297534	6	0.68373024	1.33702898	2.62842464
1	2.74863005	3.62202883	-3.13557553	1	0.44193020	0.83702898	3.57222462
6	3.03123021	-3.47567105	1.34182465	1	0.99393022	2.37072897	2.82322454
1	3.36723018	-3.17287111	2.33932447	1	1.49353027	0.80742902	2.12852454
1	3.91163015	-3.58417106	0.69972461	1	-3.25956988	-3.35787106	1.75042474
1	2.57433009	-4.46597099	1.44722474	1	-2.16836977	-3.62937117	0.38562465
6	1.48443019	-3.17077112	-0.55417538	1	-3.92586994	-3.84487104	0.17322466
1	0.73093021	-2.56337118	-1.05537534	1	-3.25286984	2.08492899	3.45172453
1	1.05433023	-4.15387106	-0.32487535	1	-1.80076981	1.35172904	4.14212465
1	2.31233001	-3.32297111	-1.25547528	1	-2.70857000	0.47452897	2.88202453
6	0.85703027	-2.39537120	1.77132463	1	-1.16546977	3.09582877	2.48072457
1	1.21743023	-1.93897104	2.69992447	1	-4.30616999	-1.44027102	0.55742460
1	0.46873018	-3.39067101	2.02032447	1	-4.74786997	4.40302896	0.22682464
1	0.00403020	-1.81227100	1.41822469				

I-5_Mg

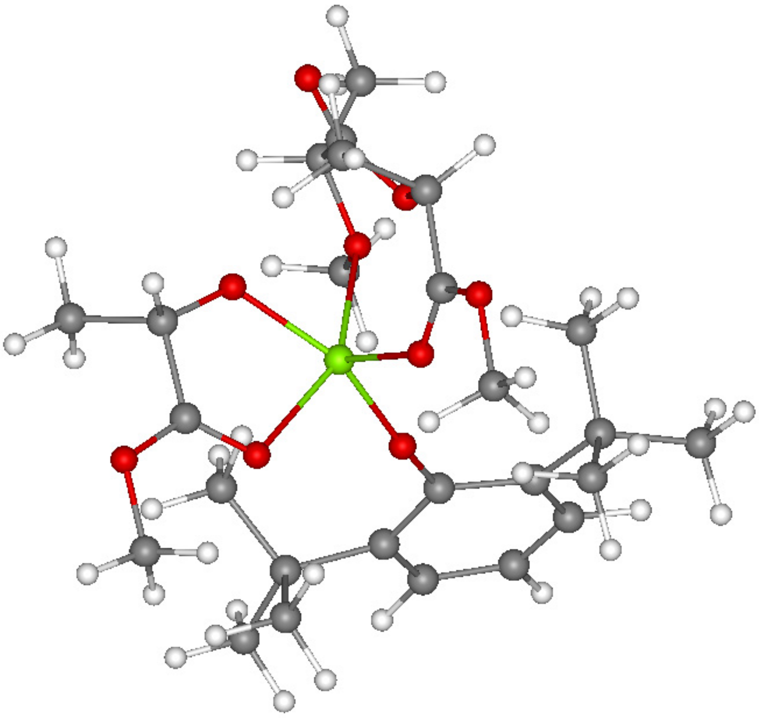

Zero-point vibrational energy	1732232.6 (Joules/Mol)
	414.01353 (Kcal/Mol)
Zero-point correction=	0.659773 (Hartree/Particle)
Thermal correction to Energy=	0.702782
Thermal correction to Enthalpy=	0.703726
Thermal correction to Gibbs Free Energy=	0.583494
Sum of electronic and zero-point Energies=	-1892.139992
Sum of electronic and thermal Energies=	-1892.096982
Sum of electronic and thermal Enthalpies=	-1892.096038
Sum of electronic and thermal Free Energies=	-1892.216270

cartesian

8	-1.04489851	-0.14175490	-0.34693706	1	5.26204109	-0.99009454	0.87993991
6	-2.31719851	-0.48455489	-0.20663702	1	5.43534470	-0.71179885	-0.88623255
6	-3.29779863	0.49164516	0.17816296	1	4.32500458	0.31747067	0.08051318 6
6	-4.59919834	0.05954510	0.45456296		2.53050137	2.52534509	0.56006300
1	-5.34839869	0.77524513	0.77626300	6	1.80100155	2.86144519	-0.73913699
6	-4.98309851	-1.26985490	0.31816298	8	2.14000154	4.01234484	-1.31703711
6	-4.05849838	-2.18895483	-0.16623703	6	1.45110154	4.33724499	-2.53913689
1	-4.39029837	-3.21015477	-0.32293701	6	2.32820153	3.64704514	1.59256291
6	-2.73819852	-1.83375490	-0.45863706	1	3.60670137	2.52514529	0.28956294
6	-2.97589850	1.99944520	0.21776296	8	0.96440142	2.10094523	-1.22443712
6	-1.78919852	-2.87915492	-1.07573712	1	0.37490150	4.38654518	-2.36723685
6	-1.95089853	2.34704518	1.31306291	1	-5.99969864	-1.57815492	0.55006295
1	-0.99609846	1.84194505	1.16296291	1	2.69260144	4.61404514	1.23286295
1	-1.75549841	3.42674518	1.32586288	1	2.87720132	3.37734509	2.49836302
1	-2.32979846	2.06564522	2.30196309	1	1.26870155	3.73624516	1.85146296
6	-2.45539856	2.44224524	-1.16523707	8	0.65660149	-0.83315486	2.11726308
1	-1.56719840	1.88424516	-1.46033704	6	2.89800143	-1.23195493	-1.57773709
1	-3.22919846	2.28604531	-1.92513704	6	3.59710145	-1.67355490	-0.30463701
1	-2.20719862	3.51194525	-1.15333712	8	2.60580134	-1.51735497	0.71046299
6	-4.22129869	2.85504508	0.51296300	6	3.03030133	-1.05935490	1.91956294
1	-5.01299858	2.70014524	-0.22713703	6	1.86750150	-0.85025489	2.87316298
1	-4.63659859	2.66234517	1.50826287	8	3.56730151	-1.55145490	-2.67383695
1	-3.94379854	3.91484499	0.47836298	6	2.97270131	-1.13485491	-3.92473698
6	-2.51079845	-4.19555521	-1.41723704	1	2.89040136	-0.04765489	-3.95423698
1	-2.89509869	-4.70635509	-0.52773702	8	4.19260120	-0.93055487	2.22136307
1	-3.34329867	-4.04215479	-2.11203694	8	1.83650160	-0.62475485	-1.59373713
1	-1.80219853	-4.87895489	-1.89933705	1	3.97987866	-2.70012736	-0.33372694
6	-1.21079850	-2.34125471	-2.40103698	6	1.90570152	-1.93855488	3.94756293
1	-0.66859859	-1.40715492	-2.25193691	1	1.98400140	-1.58115494	-4.03413677
1	-0.52969849	-3.08045483	-2.84453702	1	3.65120149	-1.49425495	-4.69553709
1	-2.01699853	-2.15625477	-3.11973691	6	-0.52229851	-0.65255487	2.90766311
6	-0.65319854	-3.24155474	-0.10213703	1	-0.70889848	-1.52945495	3.53446293

1	-1.06009841	-3.68305469	0.81466299	1	-0.43369853	0.24314512	3.53456306
1	0.02060148	-3.98005486	-0.55713701	1	-1.34899843	-0.53375489	2.20966315
1	-0.06499851	-2.37115479	0.18496297	1	2.90920138	-1.97875488	4.37786293
12	0.75770146	0.28744513	0.04916297	1	1.20510149	-1.72195494	4.75676298
8	2.10580158	1.30224502	1.00806296	1	1.66240156	-2.91665483	3.52256298
1	1.83870161	5.30904484	-2.84113693	1	2.03170156	0.13764511	3.31736302
1	1.65980160	3.58624530	-3.30383706	6	4.73370457	-0.69674551	-0.03919368

I-5_Zn

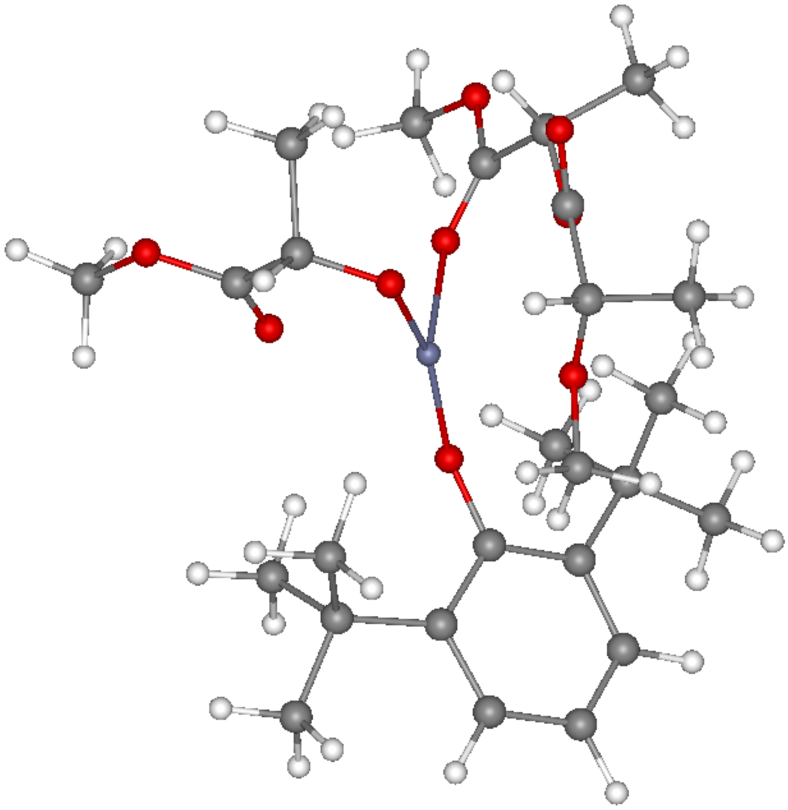

Zero-point vibrational energy	1729032.2 (Joules/Mol)
	413.24861 (Kcal/Mol)
Zero-point correction=	0.658554 (Hartree/Particle)
Thermal correction to Energy=	0.702328
Thermal correction to Enthalpy=	0.703272
Thermal correction to Gibbs Free Energy=	0.580004
Sum of electronic and zero-point Energies=	-3471.097411
Sum of electronic and thermal Energies=	-3471.053637
Sum of electronic and thermal Enthalpies=	-3471.052692
Sum of electronic and thermal Free Energies=	-3471.175961

cartesian

8	-0.99689466	0.09676404	0.73634225	8	2.60720539	4.08176374	0.96284229
6	-2.24199462	-0.21963596	0.36144227	6	2.14310527	4.57956409	2.23114228
6	-3.14139462	0.78336406	-0.12465774	1	2.40905046	3.25144172	-1.60557282
6	-4.40079451	0.37716404	-0.58195776	6	4.07198572	2.28808904	-0.61595452

1	-5.09119463	1.11146402	-0.98305774	8	1.19450533	2.34946418	1.18594229
6	-4.81639481	-0.94793594	-0.52465773	1	2.34980536	3.85616422	3.02244234
6	-3.98199463	-1.89143598	0.06524226	1	-5.79789448	-1.23423600	-0.89505774
1	-4.34759474	-2.90793586	0.16304226	8	0.40350533	-0.92713594	-2.14855766
6	-2.70859456	-1.56193602	0.53944224	6	2.91460538	-1.18653595	1.49134231
6	-2.79199457	2.28596425	-0.10755774	6	3.47590542	-1.72993600	0.18824226
6	-1.86769462	-2.62643576	1.27234221	8	2.42450547	-1.58083594	-0.76435775
6	-1.71819472	2.62616420	-1.15615773	6	2.78590536	-1.19143593	-2.01195765
1	-0.78759474	2.07696414	-0.99995774	6	1.58790541	-1.01963603	-2.92905760
1	-1.47279477	3.69506407	-1.12285769	8	3.66790533	-1.50973594	2.53714228
1	-2.07379460	2.39536405	-2.16625762	6	3.21940541	-1.00853598	3.81564236
6	-2.32299471	2.70426416	1.30164230	1	3.19960546	0.08216404	3.80514240
1	-1.43929458	2.15146422	1.61734223	8	3.93350530	-1.08143604	-2.37995768
1	-3.11999464	2.52466416	2.03174233	8	1.90400529	-0.50783598	1.58584225
1	-2.09009457	3.77676415	1.31634223	6	3.93870544	-3.17883587	0.26554227
6	-4.01039457	3.16756415	-0.43995774	6	1.56440520	-2.17843580	-3.93055773
1	-4.84469461	2.98946404	0.24644226	1	2.22190523	-1.38963604	4.03704214
1	-4.36959457	3.02416420	-1.46475780	1	3.94620538	-1.37663603	4.53694248
1	-3.72549486	4.22146368	-0.34515774	6	-0.75409466	-0.58253598	-2.90785766
6	-2.65929461	-3.92453575	1.51474226	1	-1.03259468	-1.38713598	-3.59675765
1	-2.93699455	-4.42863607	0.58274227	1	-0.58809471	0.34426403	-3.47215772
1	-3.56999469	-3.75033593	2.09794235	1	-1.56579471	-0.43663597	-2.19575763
1	-2.03509474	-4.62323618	2.08344245	1	4.34190559	-3.47353578	-0.70535773
6	-1.44479465	-2.10263586	2.66094232	1	3.10550547	-3.83813596	0.51974225
1	-0.84809464	-1.19403601	2.57954240	1	4.72260523	-3.28973579	1.01704228
1	-0.85899466	-2.86783576	3.18724227	1	2.54410529	-2.26943588	-4.40555763
1	-2.32799459	-1.88113594	3.27054238	1	0.82680529	-2.00513577	-4.71735764
6	-0.62679470	-3.00983596	0.44814226	1	1.32340527	-3.11843586	-3.42595768
1	-0.91929466	-3.43923593	-0.51625776	1	1.76240540	-0.07093596	-3.45045757
1	-0.03189468	-3.76153588	0.98414224	1	4.31410551	-1.08583593	-0.10265774
1	0.00950533	-2.14973593	0.24654226	1	1.07010531	4.77506399	2.19324231
30	0.67260528	0.46256405	-0.02615774	1	2.69670534	5.50176382	2.40074229
8	1.96510530	1.26256406	-1.16425776	1	4.51631212	3.25499940	-0.33728215
6	2.55720544	2.45826411	-0.81305772	1	4.52585363	1.93225455	-1.55264330
6	2.04300523	2.95036411	0.53614223	1	4.25764561	1.55524969	0.18309289

I-6_Mg

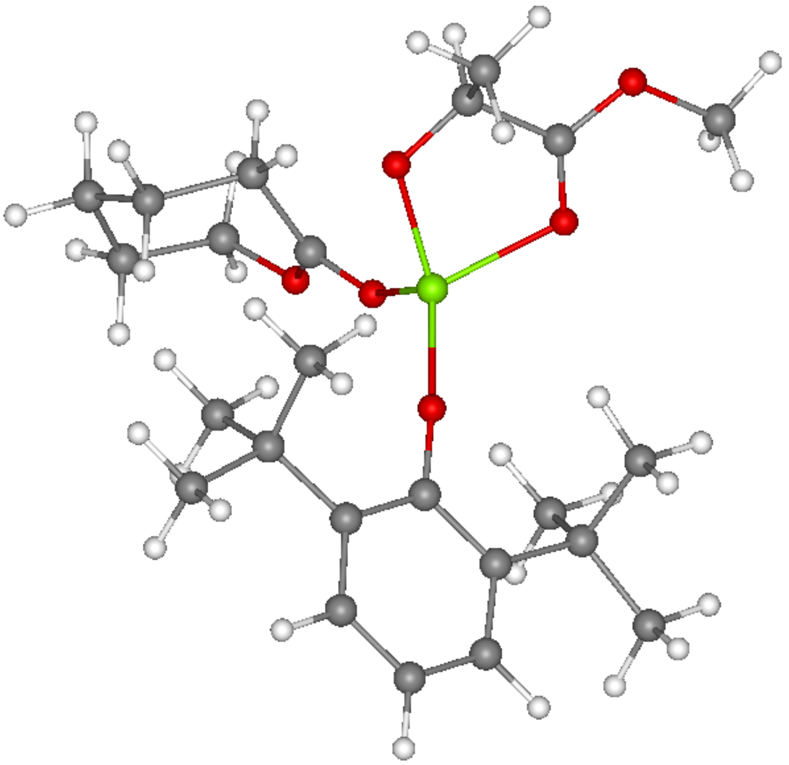

Zero-point vibrational energy	1556976.0 (Joules/Mol)
	372.12620 (Kcal/Mol)
Zero-point correction=	0.593021 (Hartree/Particle)
Thermal correction to Energy=	0.628556
Thermal correction to Enthalpy=	0.629500
Thermal correction to Gibbs Free Energy=	0.522883
Sum of electronic and zero-point Energies=	-1587.994230
Sum of electronic and thermal Energies=	-1587.958695
Sum of electronic and thermal Enthalpies=	-1587.957751
Sum of electronic and thermal Free Energies=	-1588.064367

cartesian

8	1.68690002	-0.09290000	-1.14649999	1	-2.29940009	-0.82099998	3.46429992
6	2.81329989	-0.59270000	-1.13950002	1	-0.63709998	-1.39440000	3.69499993
8	3.26430011	-1.03380001	-2.30769992	6	-2.13919997	-3.43020010	2.79229999
6	3.64229989	-0.72390002	0.10880000	1	-3.05509996	-3.19169998	3.34310007
6	4.55690002	-1.67180002	-2.43860006	1	-2.32430005	-4.32240009	2.18470001
6	3.83500004	-2.18619990	0.55379999	1	-1.37380004	-3.69169998	3.53209996
6	4.64709997	-3.02150011	-1.74300003	6	-4.33540010	1.06229997	-2.89520001
1	4.65730000	-1.78830004	-3.51810002	1	-4.52279997	0.19400001	-3.53609991
1	4.12260008	-2.16799998	1.60959995	1	-5.26020002	1.30110002	-2.35910010
1	2.87350011	-2.70970011	0.51150000	1	-4.11240005	1.91020000	-3.55299997
1	4.62039995	-0.25470001	-0.05900000	6	-2.97600007	2.14849997	-1.14530003
1	3.12479997	-0.14240000	0.87959999	1	-2.15179992	2.08690000	-0.43439999
6	4.89669991	-2.95040011	-0.23610000	1	-2.78390002	2.98799992	-1.82560003

1	5.33790016	-0.98180002	-2.10019994	1	-3.88879991	2.37179995	-0.58170003
1	3.73329997	-3.58559990	-1.96430004	6	-1.91799998	0.57270002	-2.83290005
1	5.47020006	-3.57049990	-2.21539998	1	-2.09170008	-0.30289999	-3.46819997
1	5.87519979	-2.48270011	-0.06140000	1	-1.72430003	1.43149996	-3.48810005
1	4.97189999	-3.96910000	0.15989999	1	-1.02020001	0.37700000	-2.24600005
8	-1.25670004	-0.04570000	0.07720000	12	0.40070000	0.76470000	0.21179999
6	-2.40000010	-0.71480000	-0.00510000	8	1.57869995	1.22090006	1.66520000
6	-2.66090012	-1.80879998	0.88000000	6	1.78450000	2.55789995	1.89180005
6	-3.87630010	-2.48959994	0.76090002	6	1.16540003	3.37509990	0.75599998
1	-4.09520006	-3.32150006	1.42209995	8	1.37870002	4.68090010	0.78630000
6	-4.83150005	-2.13439989	-0.18520001	6	0.78350002	5.45709991	-0.27489999
6	-4.57259989	-1.06729996	-1.03900003	6	1.21730006	3.03579998	3.24070001
1	-5.32950020	-0.80100000	-1.76909995	1	2.86069989	2.82559991	1.87670004
6	-3.38019991	-0.34040001	-0.97869998	8	0.50669998	2.84010005	-0.14600000
6	-1.64129996	-2.24090004	1.95060003	1	-0.30050001	5.33459997	-0.26859999
6	-3.14809990	0.83859998	-1.94140005	1	-5.76849985	-2.68179989	-0.25510001
6	-0.33030000	-2.70440006	1.28310001	1	1.42219996	4.09439993	3.42740011
1	0.07870000	-1.93850005	0.62159997	1	1.68009996	2.43810010	4.02950001
1	0.42190000	-2.95190001	2.04299998	1	0.13710000	2.86479998	3.28080010
1	-0.50849998	-3.59879994	0.67549998	1	1.18099999	5.14179993	-1.24100006
6	-1.37460005	-1.09010005	2.94219995	1	1.05690002	6.48939991	-0.06390000
1	-1.00039995	-0.19410001	2.44679999				

I-6_Zn

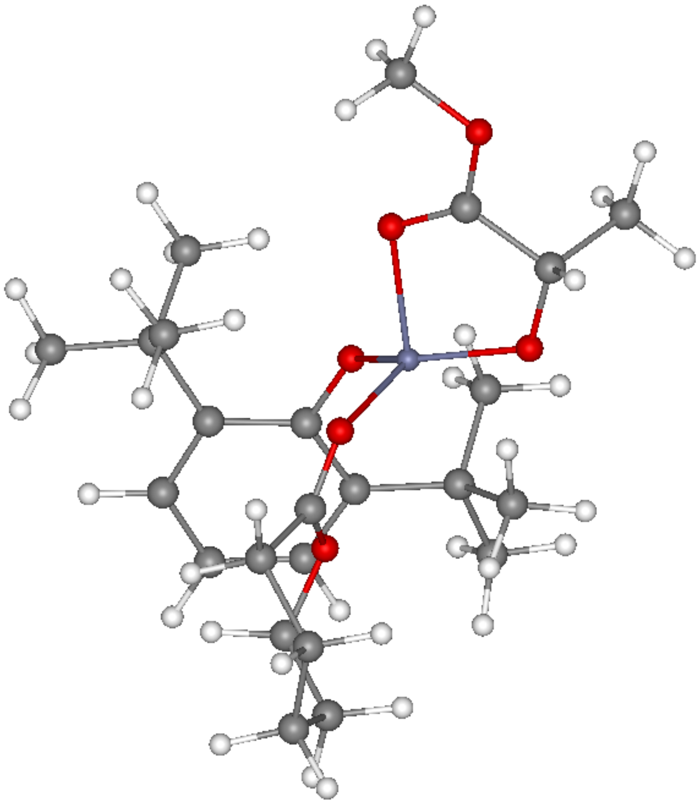

Zero-point vibrational energy	1555555.4 (Joules/Mol)
	371.78665 (Kcal/Mol)
Zero-point correction=	0.592480 (Hartree/Particle)
Thermal correction to Energy=	0.628314
Thermal correction to Enthalpy=	0.629258
Thermal correction to Gibbs Free Energy=	0.522443
Sum of electronic and zero-point Energies=	-3166.955120
Sum of electronic and thermal Energies=	-3166.919287
Sum of electronic and thermal Enthalpies=	-3166.918342
Sum of electronic and thermal Free Energies=	-3167.025157

cartesian

8	0.67680001	1.94379997	0.57520002	1	-0.84990001	-3.98510003	-2.13639998
6	-0.47979999	2.36619997	0.51389998	1	0.10150000	-3.17359996	-3.39330006
8	-1.35870004	1.64929998	-0.16410001	6	-2.42740011	-2.25419998	-3.48390007
6	-0.87220001	3.66700006	1.16199994	1	-2.98499990	-3.15660000	-3.21210003
6	-2.77649999	1.96019995	-0.18920000	1	-3.14510012	-1.44490004	-3.66050005
6	-1.38520002	4.72620010	0.16980000	1	-1.92760003	-2.45289993	-4.43809986
6	-3.09850001	3.20029998	-1.00549996	6	-1.84270000	-1.91949999	3.97300005
1	-3.20819998	1.06169999	-0.62919998	1	-2.50889993	-1.07210004	4.17140007
1	-1.30820000	5.70009995	0.66430002	1	-2.45230007	-2.82579994	3.89639997
1	-0.71280003	4.76980019	-0.69510001	1	-1.19719994	-2.03439999	4.85090017
1	-1.63370001	3.46830010	1.92630005	6	-0.01390000	-2.91300011	2.63199997
1	0.02060000	4.02320004	1.67729998	1	0.66430002	-2.81920004	1.78289998
6	-2.83010006	4.52309990	-0.28749999	1	0.58209997	-2.99740005	3.54940009
1	-3.13919997	2.03150010	0.84119999	1	-0.58560002	-3.84030008	2.51589990
1	-2.54600000	3.14829993	-1.95089996	6	-0.14800000	-0.42269999	2.98350000
1	-4.16190004	3.14549994	-1.26629996	1	-0.80360001	0.45420000	3.04539990
1	-3.49149990	4.59320021	0.58660001	1	0.38990000	-0.50120002	3.93630004
1	-3.10999990	5.35150003	-0.94779998	1	0.59410000	-0.24950001	2.20479989
8	0.13240001	-1.40359998	0.05350000	30	1.23210001	0.07420000	-0.31060001
6	-1.19579995	-1.53480005	0.14550000	8	2.41610003	0.59060001	-1.69970000
6	-1.99310005	-1.70089996	-1.03079998	6	3.75720000	0.55540001	-1.39540005
6	-3.38459992	-1.75039995	-0.88190001	6	3.98979998	0.22550000	0.07720000
1	-4.01809978	-1.85570002	-1.75660002	8	5.25430012	0.29370001	0.47850001
6	-3.99749994	-1.69000006	0.36660001	6	5.50390005	-0.02190000	1.86170006
6	-3.20300007	-1.65009999	1.50880003	6	4.52069998	-0.45550001	-2.26970005
1	-3.69589996	-1.67589998	2.47519994	1	4.22959995	1.54620004	-1.54009998
6	-1.80659997	-1.60389996	1.43789995	8	3.07570004	-0.08030000	0.84689999
6	-1.36730003	-1.88580000	-2.42919993	1	5.19089985	-1.04460001	2.07920003
6	-0.96560001	-1.70159996	2.72720003	1	-5.08129978	-1.72689998	0.45030001
6	-0.68239999	-0.60170001	-2.93510008	1	5.59709978	-0.44749999	-2.07329988
1	0.24030000	-0.36780000	-2.40199995	1	4.34730005	-0.19250000	-3.31579995

1	-0.39620000	-0.71719998	-3.98740005	1	4.13059998	-1.46519995	-2.10820007
1	-1.35529995	0.25960001	-2.86490011	1	4.96390009	0.66970003	2.51090002
6	-0.35089999	-3.04660010	-2.40219998	1	6.57910013	0.08710000	1.99310005
1	0.44380000	-2.86619997	-1.67760003				

TS-2_Mg

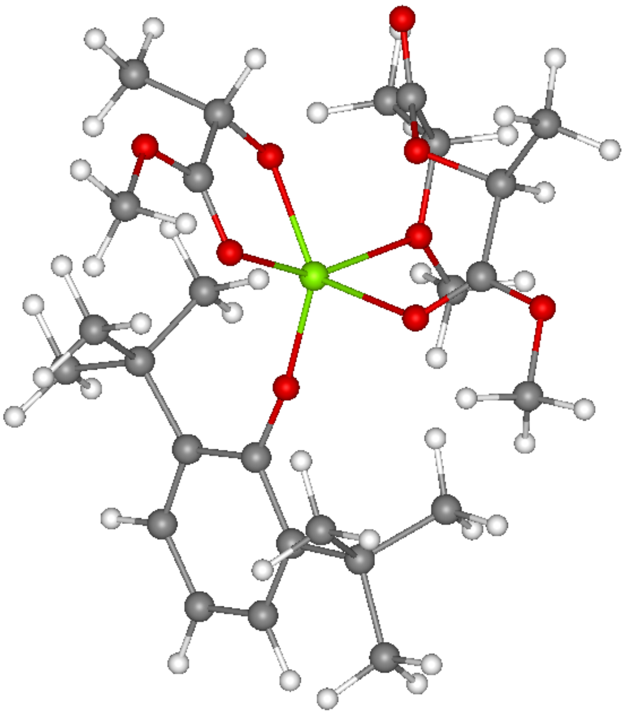

Zero-point vibrational energy	1730673.8 (Joules/Mol)
	413.64096 (Kcal/Mol)
Zero-point correction=	0.659179 (Hartree/Particle)
Thermal correction to Energy=	0.701177
Thermal correction to Enthalpy=	0.702121
Thermal correction to Gibbs Free Energy=	0.585898
Sum of electronic and zero-point Energies=	-1892.123262
Sum of electronic and thermal Energies=	-1892.081264
Sum of electronic and thermal Enthalpies=	-1892.080320
Sum of electronic and thermal Free Energies=	-1892.196543

cartesian

8	-1.38339996	0.06530000	0.02790000	8	2.35349989	3.79029989	0.18020000
6	-2.70799994	-0.01550000	-0.03020000	6	1.38409996	4.53660011	0.95200002
6	-3.51029992	1.16919994	0.09930000	1	2.87660003	1.95420003	-1.88000000
6	-4.89720011	1.06939995	-0.04820000	6	4.58199978	2.26370001	-0.59100002
1	-5.51300001	1.95869994	0.03160000	8	1.09990001	1.94379997	0.42789999
6	-5.53119993	-0.14270000	-0.29139999	1	1.30110002	4.11070013	1.95260000
6	-4.76020002	-1.29690003	-0.35609999	1	-6.61129999	-0.19000000	-0.40750000
1	-5.26989985	-2.24090004	-0.51490003	8	0.91200000	0.09310000	-2.07640004
6	-3.36929989	-1.27730000	-0.21770000	6	1.90740001	-1.48889995	2.14459991

6	-2.89030004	2.54290009	0.41639999	6	2.32179999	-2.25099993	0.88889998
6	-2.59929991	-2.60999990	-0.23540001	8	1.62709999	-1.75469995	-0.18960001
6	-1.96389997	2.99029994	-0.72970003	6	3.14100003	-0.56120002	-1.47039998
1	-1.11090004	2.31949997	-0.82709998	6	2.10719991	-0.50809997	-2.59410000
1	-1.59029996	4.00769997	-0.54970002	8	2.44910002	-1.90649998	3.28169990
1	-2.51119995	3.00539994	-1.67920005	6	2.07119989	-1.19029999	4.47300005
6	-2.11949992	2.47460008	1.74979997	1	2.37739992	-0.14510000	4.39949989
1	-1.34350002	1.71010005	1.72630000	8	4.14620018	-1.22340000	-1.52059996
1	-2.80710006	2.23810005	2.56929994	8	1.12940001	-0.53049999	2.10339999
1	-1.65209997	3.44239998	1.97580004	6	2.14980006	-3.76600003	1.06889999
6	-3.95009995	3.64779997	0.57709998	6	1.90509999	-1.87839997	-3.22059989
1	-4.66020012	3.42580009	1.38039994	1	0.99129999	-1.24349999	4.61969995
1	-4.51520014	3.82329988	-0.34470001	1	2.59579992	-1.68739998	5.28730011
1	-3.45070004	4.58879995	0.83609998	6	-0.06910000	0.42150000	-3.06620002
6	-3.52440000	-3.83159995	-0.38620001	1	-0.42960000	-0.47450000	-3.57690001
1	-4.07240009	-3.82570004	-1.33469999	1	0.35370001	1.12290001	-3.79690003
1	-4.24959993	-3.90860009	0.43079999	1	-0.89999998	0.88279998	-2.53660011
1	-2.91669989	-4.74350023	-0.36910000	1	2.48920012	-4.25409985	0.15230000
6	-1.85899997	-2.79360008	1.10420001	1	1.09689999	-4.01660013	1.22010005
1	-1.18949997	-1.95990002	1.31799996	1	2.73550010	-4.14790010	1.90999997
1	-1.27260005	-3.71989989	1.09549999	1	2.88059998	-2.28399992	-3.49740005
1	-2.58089995	-2.85700011	1.92620003	1	1.29449999	-1.81490004	-4.12449980
6	-1.62119997	-2.66350007	-1.42349994	1	1.43939996	-2.54310012	-2.49289989
1	-2.17459989	-2.60060000	-2.36789989	1	2.51600003	0.17739999	-3.35730004
1	-1.05750000	-3.60400009	-1.41980004	1	3.41030002	-2.05699992	0.79339999
1	-0.90200001	-1.84689999	-1.39489996	1	0.41290000	4.50740004	0.45950001
12	0.50840002	-0.14780000	0.08710000	1	1.77349997	5.55189991	0.99190003
8	2.99519992	0.42100000	-0.47380000	1	4.69080019	3.31660008	-0.86019999
6	3.15560007	1.78659999	-0.83029997	1	4.85790014	2.13339996	0.45780000
6	2.09599996	2.50670004	-0.00530000	1	5.26499987	1.66840005	-1.20079994

TS-2_Zn

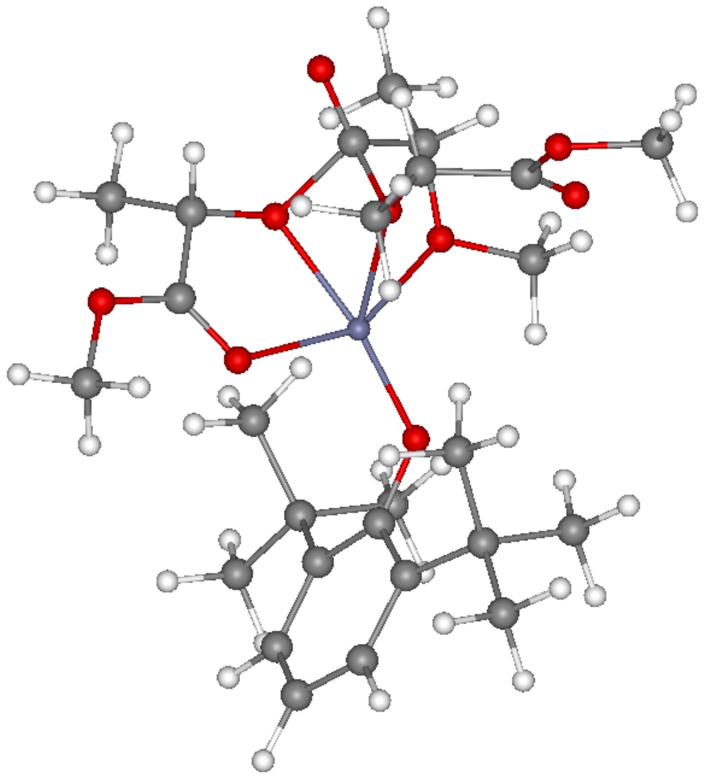

Zero-point vibrational energy	1729386.5 (Joules/Mol)
	413.33328 (Kcal/Mol)
Zero-point correction=	0.658689 (Hartree/Particle)
Thermal correction to Energy=	0.701027
Thermal correction to Enthalpy=	0.701971
Thermal correction to Gibbs Free Energy=	0.582956
Sum of electronic and zero-point Energies=	-3471.079413
Sum of electronic and thermal Energies=	-3471.037074
Sum of electronic and thermal Enthalpies=	-3471.036130
Sum of electronic and thermal Free Energies=	-3471.155145

cartesian

8	1.09979868	0.51445395	0.93076670	8	-4.83040142	2.30455399	-0.42133331
6	2.24819875	0.80185395	0.28036669	6	-5.32120132	3.36425400	0.41656667
6	2.36809874	2.03305411	-0.42933333	1	-4.34890127	-0.30684605	-0.85283333
6	3.53929877	2.26325393	-1.15863335	6	-3.19560122	0.86185396	-2.23293328
1	3.64999866	3.17705393	-1.73243332	8	-3.46940136	1.66815400	1.26176667
6	4.58819866	1.35035396	-1.16903341	1	-4.50640106	4.03145361	0.70446670
6	4.50039864	0.20515394	-0.38503331	1	5.48319864	1.54815388	-1.75423336
1	5.35339880	-0.46434605	-0.36143333	8	-1.27190137	-0.94994605	2.08206654
6	3.36029863	-0.08774605	0.37166667	6	-0.37710133	-1.97984600	-2.26993346
6	1.27409863	3.11665392	-0.36543331	6	-1.20950139	-2.88164592	-1.37233341
6	3.35999870	-1.30154610	1.32366657	8	-1.22190142	-2.34874606	-0.07583332
6	1.05149865	3.54245400	1.10066664	6	-2.73060131	-1.70444608	0.32056668
1	0.76009870	2.69085407	1.71706665	6	-2.54970121	-1.56804609	1.83806658

1	0.26279867	4.30275393	1.16116667	8	-0.27090132	-2.40884590	-3.51673341
1	1.96809852	3.97335410	1.51776659	6	0.51959866	-1.59484601	-4.41173315
6	-0.05480133	2.62445402	-0.96803331	1	0.06349866	-0.60994601	-4.52203321
1	-0.53860134	1.88795400	-0.32253331	8	-3.68690133	-2.29894590	-0.17133331
1	0.10189866	2.18225408	-1.95753336	8	0.15649866	-0.93974602	-1.88313341
1	-0.75560135	3.46155405	-1.07403338	6	-0.69340134	-4.32124615	-1.36413336
6	1.66939867	4.38145399	-1.14853334	6	-2.66970134	-2.89934587	2.55606651
1	1.78469861	4.18965387	-2.22123337	1	1.53399861	-1.48764610	-4.02583313
1	2.59869885	4.82415390	-0.77573329	1	0.51759869	-2.12934589	-5.35943317
1	0.88269871	5.13545370	-1.03503335	6	-1.23190141	-0.06324605	3.20516658
6	4.70699883	-2.04704595	1.30606663	1	-1.41940141	-0.61904603	4.13026667
1	5.54339886	-1.39624596	1.58006668	1	-1.96740139	0.73665392	3.08216667
1	4.92399883	-2.49584603	0.33006668	1	-0.22690134	0.35655394	3.22626662
1	4.67619896	-2.86074591	2.03876662	1	-1.35050142	-4.91524601	-0.72603333
6	2.28359866	-2.33994603	0.95146674	1	0.32039869	-4.36914635	-0.95833331
1	1.27789867	-2.00634599	1.21596658	1	-0.69740134	-4.74774647	-2.37003326
1	2.44839883	-3.27374601	1.50246668	1	-3.63360119	-3.35614610	2.31986666
1	2.31569862	-2.57384610	-0.11823332	1	-2.60720134	-2.76214600	3.63966656
6	3.13199878	-0.81634605	2.77006674	1	-1.87160134	-3.57334590	2.23816657
1	3.94569874	-0.15444605	3.08566666	1	-3.33320117	-0.87364608	2.16296673
1	3.10269880	-1.66914606	3.45966673	1	-2.23830128	-2.87464595	-1.76783335
1	2.19289875	-0.26774603	2.85526657	1	-5.78640127	2.95485401	1.31566668
30	-0.27900133	-0.52094603	0.18936668	1	-6.05650139	3.89525390	-0.18623331
8	-2.40100121	-0.33234605	-0.28063333	1	-4.04760122	1.40945387	-2.64113331
6	-3.49690127	0.38565397	-0.81533331	1	-3.00640130	-0.00754605	-2.86733341
6	-3.89430118	1.51335406	0.13616668	1	-2.31790137	1.51135397	-2.25503349

I-3'_Mg

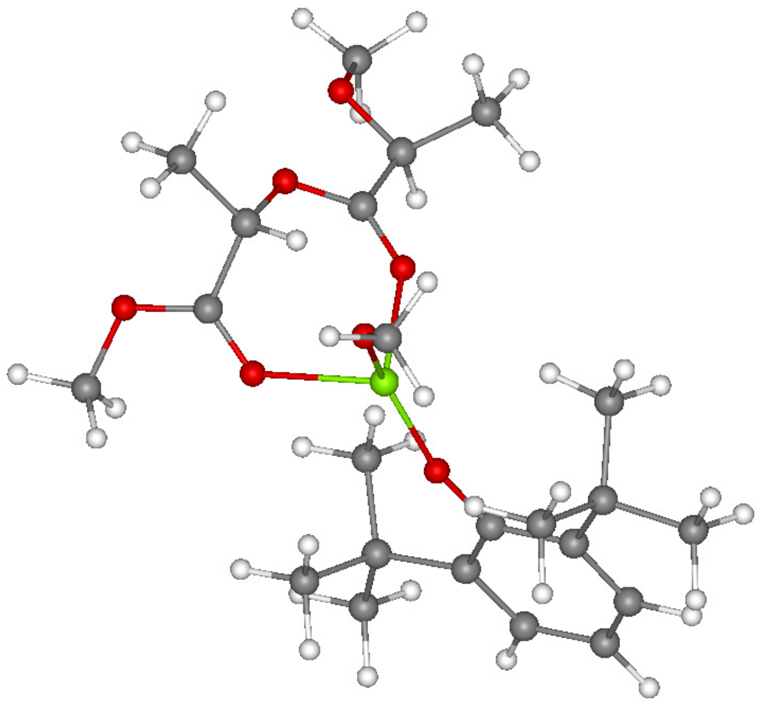

Zero-point vibrational energy	1539976.9 (Joules/Mol)
	368.06332 (Kcal/Mol)
Zero-point correction=	0.586546 (Hartree/Particle)
Thermal correction to Energy=	0.624217
Thermal correction to Enthalpy=	0.625161
Thermal correction to Gibbs Free Energy=	0.514822
Sum of electronic and zero-point Energies=	-1625.065452
Sum of electronic and thermal Energies=	-1625.027782
Sum of electronic and thermal Enthalpies=	-1625.026837
Sum of electronic and thermal Free Energies=	-1625.137176

cartesian

8	1.29318333	-0.04103186	0.50311595	12	-0.30251670	-0.89703184	0.91251600
6	2.48198318	0.32556814	0.03901595	6	-0.84241670	-3.62293196	2.30591607
6	2.79548335	1.71746814	-0.08008404	8	-1.06631660	-2.37193179	1.74321592
6	4.04838371	2.08086824	-0.58188403	1	5.96158361	1.44506812	-1.34768403
1	4.30718327	3.12986803	-0.68158400	8	-4.30951643	0.45886815	-2.65358400
6	4.99398327	1.13406813	-0.96108413	6	-2.82661676	0.39556813	1.86841595
6	4.68758345	-0.21603185	-0.83158410	6	-3.55291677	-0.71353185	1.11011589
1	5.44018364	-0.94073188	-1.12398410	8	-3.70841670	-0.26663184	-0.27448404
6	3.45488334	-0.65453184	-0.33818406	6	-2.68121672	-0.36863184	-1.10238409
6	1.79018331	2.80616808	0.33981594	6	-2.99241686	-0.02633185	-2.55118394
6	3.17678332	-2.16313195	-0.20288405	8	-3.60921669	1.18026817	2.58341599
6	1.45948339	2.67336822	1.84031594	6	-2.95341682	2.24246812	3.31541610
1	1.04428339	1.69296813	2.07441592	1	-2.24071670	1.81896818	4.02401590
1	0.73558331	3.44206810	2.14191604	8	-1.54801679	-0.70863187	-0.77998400
1	2.36458325	2.81176805	2.44191599	8	-1.60951662	0.54826814	1.80801594

6	0.50598329	2.70886803	-0.50858402	6	-4.93751669	-1.04503191	1.61741590
1	0.04308331	1.72586811	-0.41728404	6	-2.72431684	-1.25453186	-3.42878389
1	0.73568332	2.87966824	-1.56668413	1	-2.43361664	2.90446806	2.62231612
1	-0.22001669	3.47036815	-0.19418404	1	-3.75381684	2.76706815	3.83261609
6	2.34358335	4.22706795	0.13421595	6	-4.54161644	1.25106812	-3.80618405
1	2.57638335	4.43416834	-0.91578400	1	-4.46451664	0.66986817	-4.73378420
1	3.24408317	4.41116810	0.72951591	1	-3.84311676	2.09806824	-3.85598397
1	1.58958340	4.95726824	0.45061594	1	-5.55891657	1.63466811	-3.71718407
6	4.37698364	-3.02493191	-0.63628399	1	-5.35431671	-1.85583186	1.01581597
1	5.26578331	-2.83173180	-0.02668405	1	-5.60451651	-0.18283185	1.56581593
1	4.64048338	-2.87253189	-1.68828404	1	-4.87561655	-1.38413191	2.65421605
1	4.12188339	-4.08383179	-0.51458406	1	-1.70931673	-1.62413180	-3.27138400
6	2.00318336	-2.57923174	-1.11338413	1	-2.83081675	-0.99083185	-4.48348427
1	1.11038327	-1.97403181	-0.94648409	1	-3.43821669	-2.04893184	-3.19608402
1	1.74028325	-3.63193178	-0.95548403	1	-2.26811671	0.76206815	-2.81078386
1	2.27848315	-2.45193195	-2.16618395	1	-2.87851667	-1.58413184	1.15081596
6	2.89828324	-2.52813196	1.26971591	1	0.21998331	-3.80903196	2.53531599
1	3.77968335	-2.31343174	1.88361597	1	-1.17081666	-4.44053173	1.63961589
1	2.67098331	-3.59643197	1.36631596	1	-1.39521670	-3.74463177	3.25431609
1	2.06608319	-1.96153188	1.69031596				

I-3'_Zn

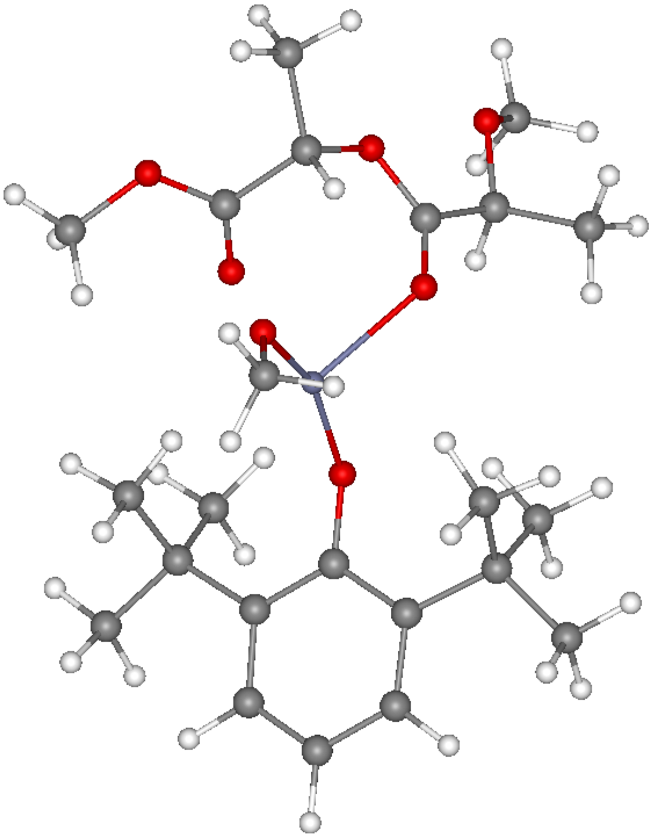

Zero-point vibrational energy	1538778.1 (Joules/Mol)
	367.77679 (Kcal/Mol)
Zero-point correction=	0.586090 (Hartree/Particle)
Thermal correction to Energy=	0.624892
Thermal correction to Enthalpy=	0.625837
Thermal correction to Gibbs Free Energy=	0.511344
Sum of electronic and zero-point Energies=	-3204.028271
Sum of electronic and thermal Energies=	-3203.989469
Sum of electronic and thermal Enthalpies=	-3203.988524
Sum of electronic and thermal Free Energies=	-3204.103017

cartesian

8	1.01193464	-0.16329706	-0.35704923	30	-0.24766538	0.54090291	0.80235076
6	2.35593462	-0.14299706	-0.21484925	6	-0.16396537	1.57720292	3.47995067
6	3.11713457	0.89880288	-0.82534927	8	-0.85566539	1.51640296	2.25565076
6	4.50593472	0.88570291	-0.65664923	1	6.23203468	-0.09739708	0.17715076
1	5.11153460	1.67070293	-1.09594917	8	-4.46236515	-1.97319710	-1.62824917
6	5.15143490	-0.11629707	0.05805077	6	-2.76896524	2.03580308	0.34045076
6	4.40383482	-1.15749705	0.59495074	6	-3.66616535	0.96920288	0.95715076
1	4.93133450	-1.94629705	1.11985075	8	-3.82726526	-0.10319708	-0.00094923
6	3.01173472	-1.21289706	0.46595079	6	-2.87776542	-1.02159715	-0.09234923
6	2.46263456	2.01710296	-1.66164923	6	-3.17746544	-2.14289689	-1.07724917
6	2.24243474	-2.42489696	1.03165078	8	-3.10636544	3.26320291	0.69135076
6	1.61993468	2.94370294	-0.76484925	6	-2.17066526	4.30000305	0.32065076
1	0.76983464	2.42680311	-0.31514925	1	-1.20526528	4.08870268	0.78115076
1	1.20983469	3.77370310	-1.35344923	8	-1.83006537	-1.01319706	0.54605079
1	2.22913456	3.36540294	0.04165076	8	-1.82336533	1.76290286	-0.39124924
6	1.59163463	1.42320287	-2.78814936	6	-5.05586529	1.42570293	1.35445082
1	0.79663461	0.79180288	-2.39234924	6	-2.99356532	-3.50059700	-0.39404923
1	2.20583463	0.81790292	-3.46404934	1	-2.07056546	4.34530306	-0.76454926
1	1.13943470	2.23020291	-3.37814927	1	-2.59786534	5.22220325	0.70995075
6	3.51003456	2.91470313	-2.34664941	6	-4.61206532	-2.54009700	-2.91794920
1	4.17563486	2.34270310	-3.00154924	1	-4.50566530	-3.63289690	-2.90874934
1	4.12453461	3.46990299	-1.62974918	1	-3.88636541	-2.11699700	-3.62654924
1	2.99463463	3.65410304	-2.96954942	1	-5.62046528	-2.29019690	-3.25154924
6	3.19163465	-3.50049686	1.59085083	1	-5.60536528	0.58300292	1.77975082
1	3.76913476	-3.14439702	2.45065069	1	-5.60956526	1.80610287	0.49345076
1	3.89083481	-3.86599708	0.83195072	1	-4.98596525	2.21410298	2.10505080
1	2.60113478	-4.35819721	1.93135083	1	-1.99816537	-3.57649708	0.04625076
6	1.41433465	-3.10049701	-0.08054924	1	-3.10866523	-4.30409718	-1.12524915
1	0.69933462	-2.40559697	-0.52204925	1	-3.74556541	-3.63489699	0.38815075
1	0.87003464	-3.96259689	0.32595077	1	-2.40666533	-2.02819705	-1.85664916
1	2.07353473	-3.46599698	-0.87574923	1	-3.09846544	0.60420293	1.82115078
6	1.32953465	-2.01029706	2.20215058	1	-0.70746541	2.24120307	4.16845083

1	1.89443469	-1.46889710	2.96765065	1	0.85523456	1.98080289	3.37445068
1	0.88103467	-2.89389706	2.67185068	1	-0.08026537	0.59560293	3.97285080
1	0.49633461	-1.38199711	1.87845075				

TS-3_Mg

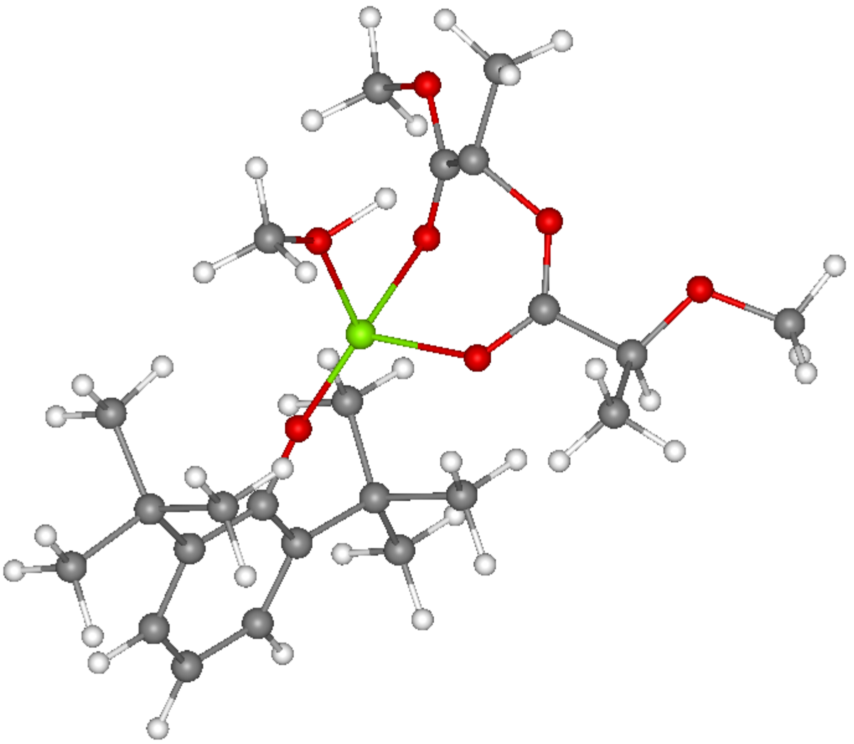

Zero-point vibrational energy	1528115.5 (Joules/Mol)
	365.22838 (Kcal/Mol)
Zero-point correction=	0.582028 (Hartree/Particle)
Thermal correction to Energy=	0.619744
Thermal correction to Enthalpy=	0.620688
Thermal correction to Gibbs Free Energy=	0.510542
Sum of electronic and zero-point Energies=	-1625.047888
Sum of electronic and thermal Energies=	-1625.010172
Sum of electronic and thermal Enthalpies=	-1625.009228
Sum of electronic and thermal Free Energies=	-1625.119374

cartesian

8	-1.37312460	-0.08767972	-0.29810292	1	-1.88952458	-2.24097991	-1.12820292
6	-2.61672449	0.28032029	0.00159710	12	0.41157544	-0.48917970	-0.47000289
6	-3.02362442	1.64122033	-0.16530290	6	1.20617545	-3.57767987	-0.40260291
6	-4.33852482	1.98692036	0.16099711	8	1.27987540	-2.23747969	-0.85980284
1	-4.67252493	3.01242018	0.04429710	1	-6.26782465	1.34992027	0.88189709
6	-5.25142479	1.05192029	0.63619709	8	4.91777515	1.06372035	2.03959703
6	-4.84842491	-0.26987973	0.79029715	6	2.69717550	-0.08387972	-2.10630298
1	-5.57502460	-0.98587972	1.15959704	6	3.48517561	-0.84487975	-1.15590298
6	-3.55002451	-0.68917972	0.48429713	8	3.76997542	-0.08527973	0.06789710
6	-2.05622458	2.71572018	-0.69500291	6	2.92587543	0.18692029	1.01979709

6	-3.15792441	-2.16727972	0.66459715
6	-1.57192457	2.34842014	-2.11240292
1	-1.08512461	1.37342036	-2.13890290
1	-0.86192465	3.09862018	-2.48190284
1	-2.42102456	2.31632018	-2.80400300
6	-0.86422455	2.88782024	0.26899710
1	-0.34372461	1.94692028	0.45269713
1	-1.21112466	3.26252031	1.23839712
1	-0.14202459	3.60822010	-0.13460290
6	-2.71882439	4.10032034	-0.80930293
1	-3.07082438	4.47422028	0.15799710
1	-3.56282449	4.10002041	-1.50700295
1	-1.98392451	4.81992054	-1.18800294
6	-4.32792473	-3.02597976	1.17799711
1	-5.17512465	-3.02757978	0.48419711
1	-4.68612480	-2.69677973	2.15899706
1	-3.99312425	-4.06407976	1.28539705
6	-2.03222442	-2.30267978	1.71019709
1	-1.15292466	-1.71287966	1.44469702
1	-1.72742462	-3.35127974	1.81689703
1	-2.37682438	-1.95127964	2.68879700
6	-2.73122454	-2.77647972	-0.68620288
1	-3.56102443	-2.73477983	-1.40020287
1	-2.45142460	-3.83017969	-0.56110287
6	3.53707552	0.87472028	2.23499703
8	3.07777548	-0.19027972	-3.37900305
6	2.13987541	0.27032030	-4.37120247
1	1.20757544	-0.29367971	-4.30350256
8	1.70857537	-0.04427972	1.02469707
8	1.62407541	0.50012028	-1.80670297
6	4.80127525	-1.43237972	-1.60110295
6	3.21527553	0.07152028	3.49969697
1	1.93417537	1.33292031	-4.23720264
1	2.62647557	0.08962028	-5.32820272
6	5.46407509	2.13082027	2.79349709
1	5.42277527	1.94372034	3.87459707
1	4.95037508	3.07812023	2.57609701
1	6.50957537	2.21842027	2.49399710
1	5.19717550	-2.08657980	-0.81960285
1	5.55617523	-0.66867977	-1.82170296
1	4.65187550	-2.03517985	-2.49910283
1	2.13897538	-0.08427972	3.59329700
1	3.56127548	0.61132026	4.38439751
1	3.71867561	-0.89847976	3.46889710
1	3.02057552	1.84692037	2.29049706
1	2.37567544	-1.80617964	-0.94050288
1	0.17457542	-3.92957973	-0.47980291
1	1.52707541	-3.66727972	0.64349711
1	1.83917534	-4.22427940	-1.02200294

TS-3_Zn

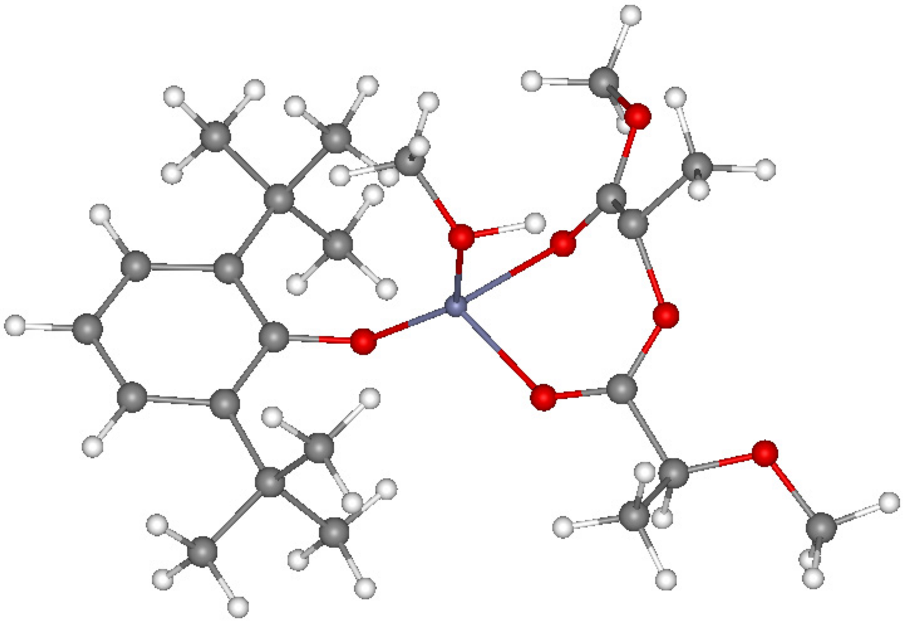

Zero-point vibrational energy	1527149.8 (Joules/Mol)
	364.99757 (Kcal/Mol)
Zero-point correction=	0.581661 (Hartree/Particle)
Thermal correction to Energy=	0.619657
Thermal correction to Enthalpy=	0.620601
Thermal correction to Gibbs Free Energy=	0.509181
Sum of electronic and zero-point Energies=	-3204.003690
Sum of electronic and thermal Energies=	-3203.965693
Sum of electronic and thermal Enthalpies=	-3203.964749
Sum of electronic and thermal Free Energies=	-3204.076170

cartesian

8	1.28206956	0.15654349	0.74668121	30	-0.34303045	-0.26855651	-0.03311881
6	2.51706934	0.43804345	0.28988117	6	-0.19143043	-1.31765652	-2.92991877
6	3.51146936	-0.58725649	0.29828116	8	-0.91213042	-0.59475648	-1.93181884
6	4.79676962	-0.26885653	-0.15151881	1	6.13636923	1.23184347	-0.92371881
1	5.56936932	-1.02995658	-0.16461881	8	-5.44423056	1.18924344	0.83648121
6	5.12896967	1.01114345	-0.57911879	6	-2.28303051	-2.33975649	0.10928119
6	4.16576958	2.01204348	-0.53711879	6	-3.09873056	-1.58925653	-0.81551880
1	4.45376968	3.01054358	-0.84691882	8	-3.74243045	-0.42845652	-0.19211881
6	2.85856938	1.76874340	-0.10111882	6	-3.17923045	0.66274351	0.23068120
6	3.21066952	-2.01215649	0.80378121	6	-4.15593052	1.73954344	0.68908119
6	1.84486949	2.93024349	-0.05601881	8	-2.40083051	-3.66955638	0.01408119
6	2.19226956	-2.70865655	-0.11821881	6	-1.43813038	-4.45725632	0.73808122
1	1.21906960	-2.21245646	-0.09161881	1	-0.42313045	-4.21545649	0.42018119
1	2.03936934	-3.74795651	0.19848119	8	-1.96833038	0.92464346	0.28078118
1	2.54746938	-2.72015643	-1.15421891	8	-1.40623045	-1.83575654	0.86248118
6	2.68316936	-1.97695661	2.25318122	6	-4.18533039	-2.30135655	-1.58211887
1	1.77036953	-1.38675654	2.33508134	6	-4.11013031	2.90794349	-0.30291882
1	3.43406940	-1.53805661	2.91898131	1	-1.53303039	-4.28075647	1.81068110
1	2.47796941	-2.99565649	2.60618114	1	-1.67903042	-5.49195623	0.49818116
6	4.46626949	-2.90295649	0.81088120	6	-6.28753042	1.93114340	1.69868112
1	5.25636959	-2.49435639	1.44918120	1	-6.53123045	2.92274356	1.29538119
1	4.87806940	-3.05775642	-0.19231881	1	-5.83633041	2.05274367	2.69388127
1	4.20386934	-3.88925648	1.20928109	1	-7.21103048	1.35814345	1.79678118
6	2.50846934	4.29194355	-0.33541882	1	-4.60723066	-1.62985659	-2.33511877
1	2.92436957	4.35984373	-1.34611881	1	-5.00253057	-2.65025640	-0.93941879
1	3.30536938	4.51454353	0.38188118	1	-3.76813054	-3.16605639	-2.10221863
1	1.75536954	5.08274364	-0.24521880	1	-3.08713055	3.27174354	-0.41891882
6	1.19166958	3.04734349	1.33688116	1	-4.72663069	3.73444366	0.05858119
1	0.66396958	2.13784361	1.62108111	1	-4.49193048	2.59114361	-1.27711880
1	0.48286957	3.88464355	1.34898114	1	-3.76913047	2.07984352	1.66178119
1	1.95536959	3.24414349	2.09738135	1	-1.90093040	-1.06735659	-1.66051888

6	0.77576959	2.74114347	-1.14841890	1	-0.66963047	-1.17075658	-3.90381885
1	1.23636961	2.73784351	-2.14201880	1	-0.15233044	-2.39075637	-2.70681882
1	0.04036956	3.55394363	-1.11631882	1	0.82886958	-0.93195647	-2.97451878
1	0.22316955	1.80554342	-1.04511881				

S5. Synthesis of random εCL copolymers using PLLA and other comonomers

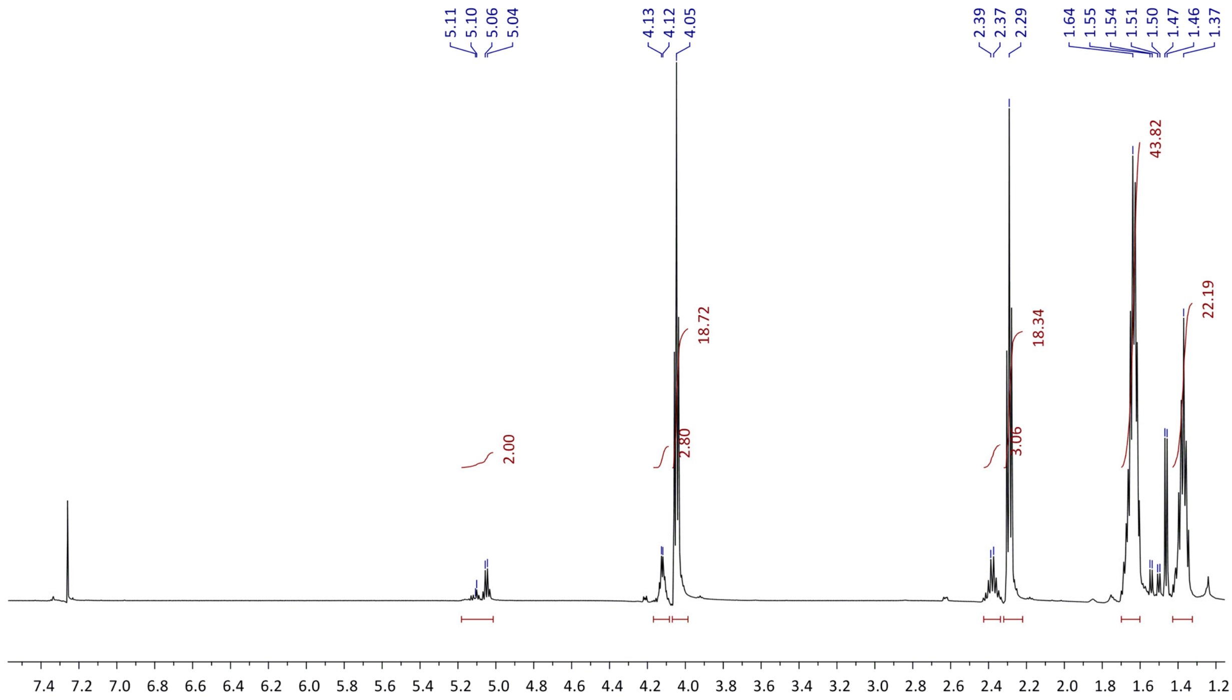

Figure S7. ^{1}H NMR spectrum ($CDCl_3$, 600 MHz) of *L*LA/εCL copolymer obtained by transesterification of PLLA (Table 4, Entry 2, re-precipitated from MeOH)

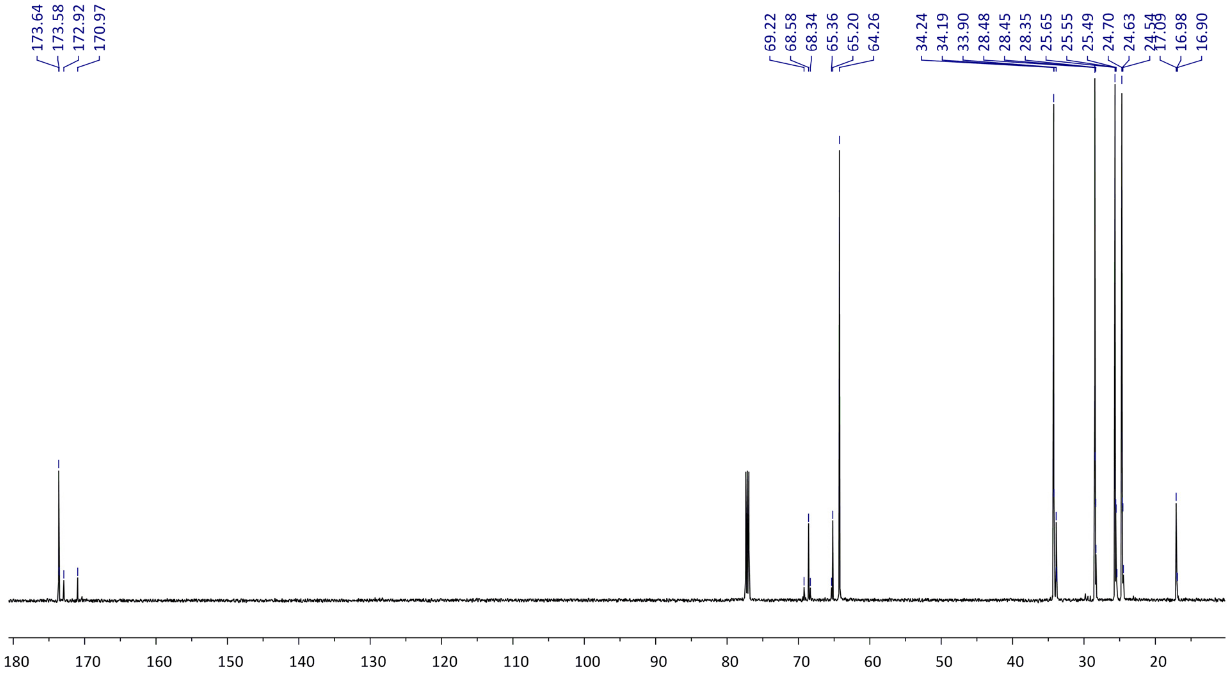

Figure S8. ^{13}C NMR spectrum ($CDCl_3$, 151 MHz) of *L*LA/εCL copolymer obtained by transesterification of PLLA (Table 4, Entry 2, re-precipitated from MeOH)

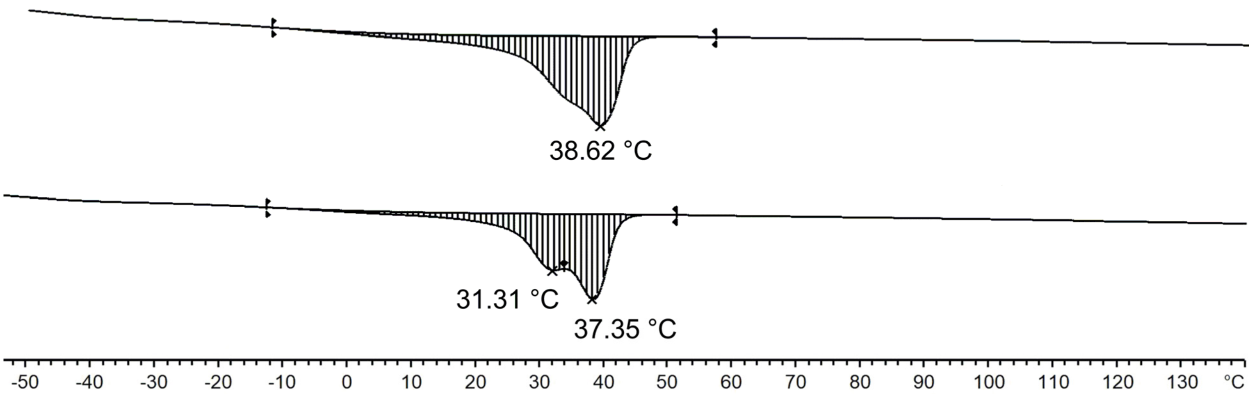

Figure S9. DSC curves (second heat) of *L*LA/εCL copolymers obtained in the presence of **3**/BnOH by transesterification of PLLA after 5 h (top) and 15 h (bottom) (Table 4, Entries 1 and 2, respectively).

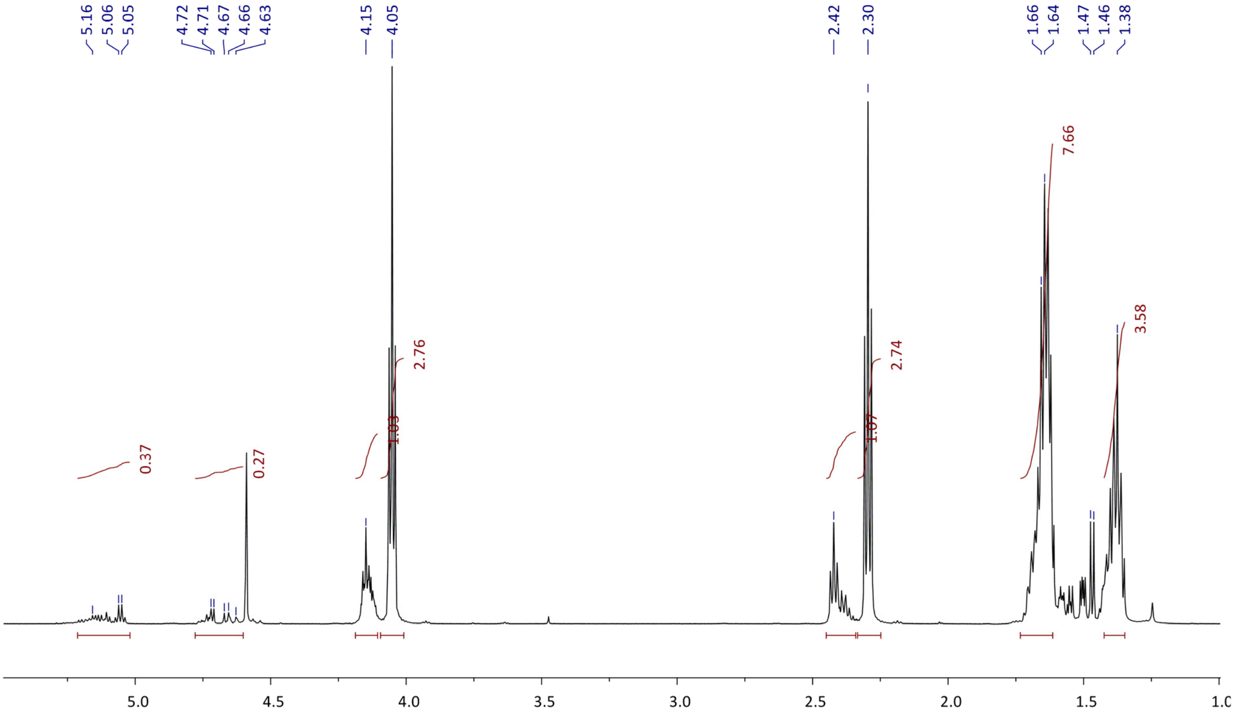

Figure S10. ^{1}H NMR spectrum ($CDCl_3$, 600 MHz) of MeGL/εCL copolymer (Table 4, Entry 3c, re-precipitated from MeOH)

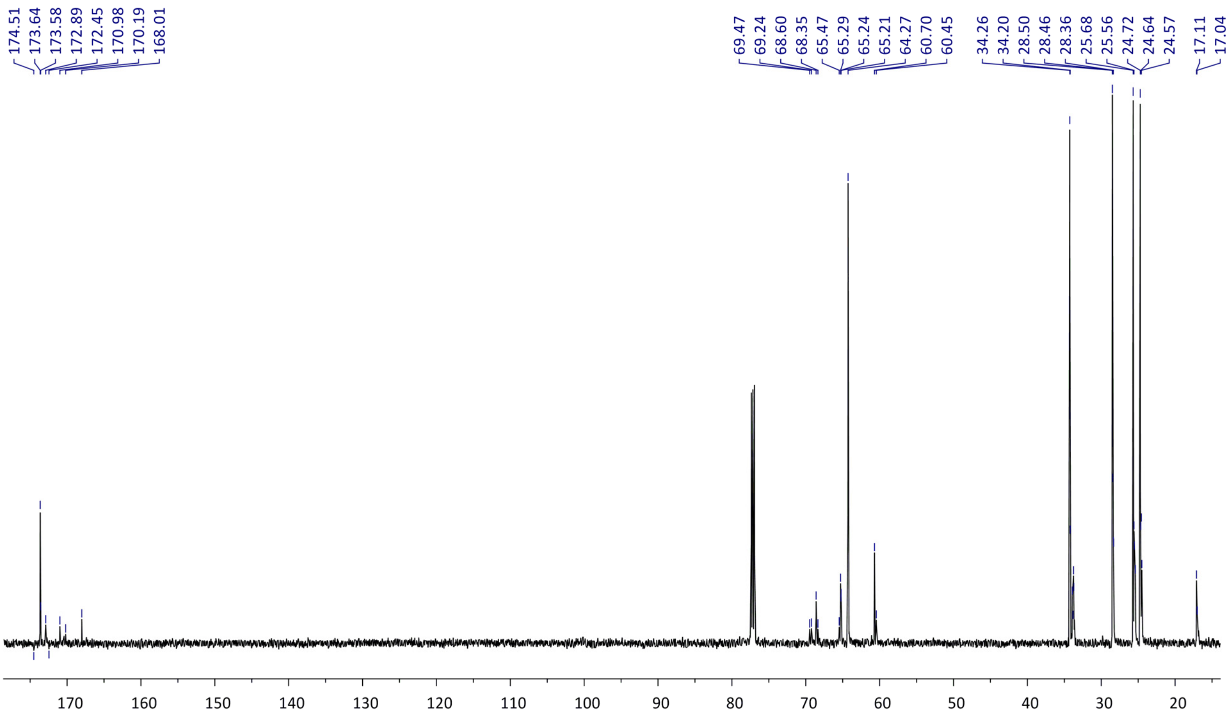

Figure S11. ^{13}C NMR spectrum ($CDCl_3$, 151 MHz) of MeGL/εCL copolymer (Table 4, Entry 3c, re-precipitated from MeOH)

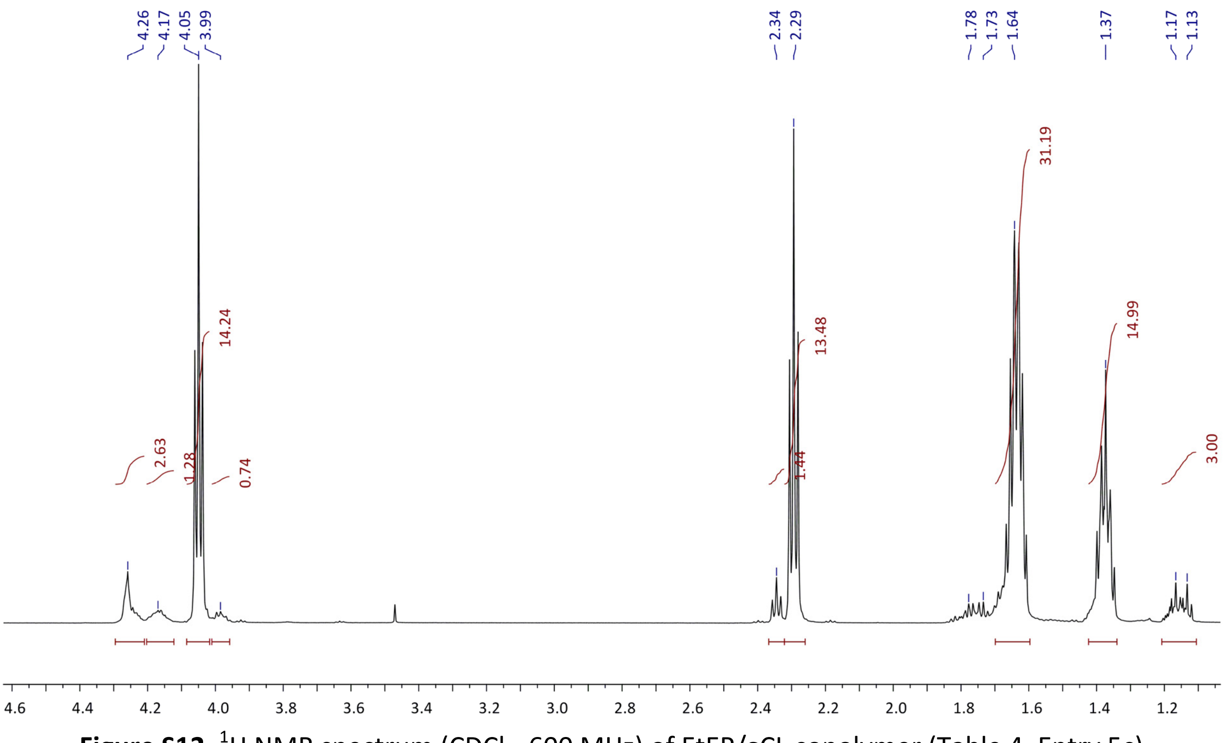

Figure S12. ^{1}H NMR spectrum ($CDCl_3$, 600 MHz) of EtEP/εCL copolymer (Table 4, Entry 5c)

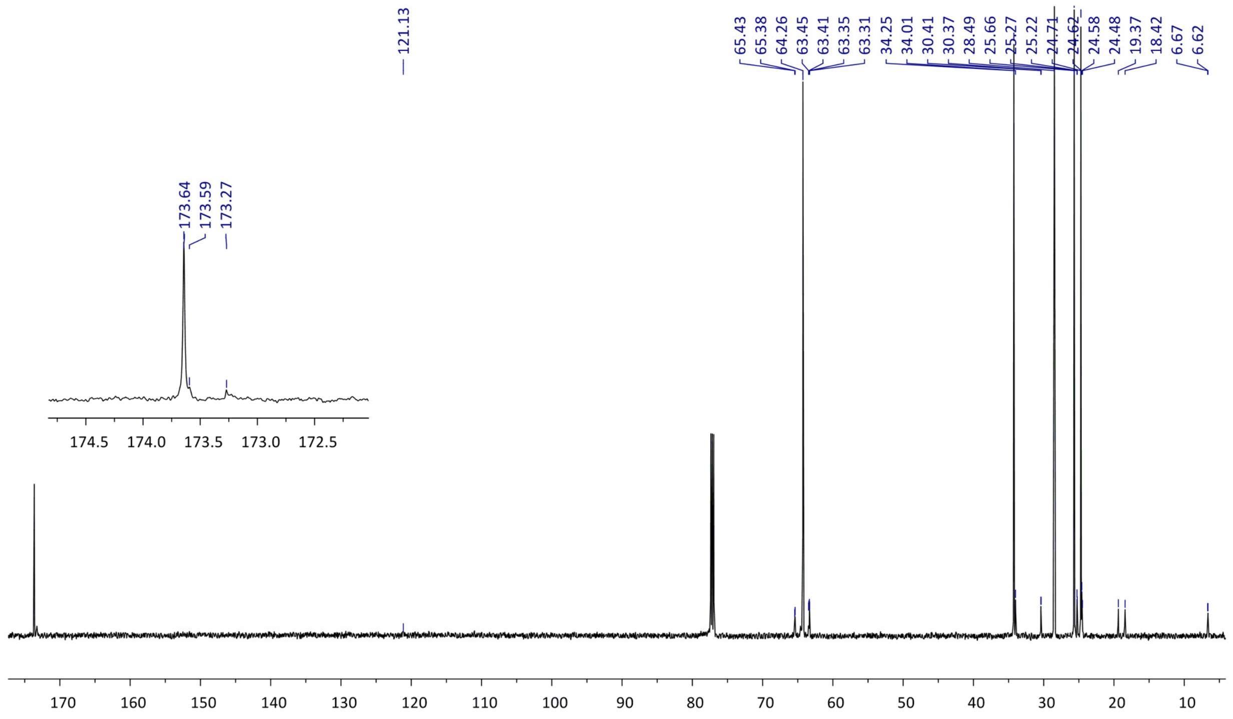

Figure S13. ^{13}C NMR spectrum ($CDCl_3$, 151 MHz) of EtEP/εCL copolymer (Table 4, Entry 5c)

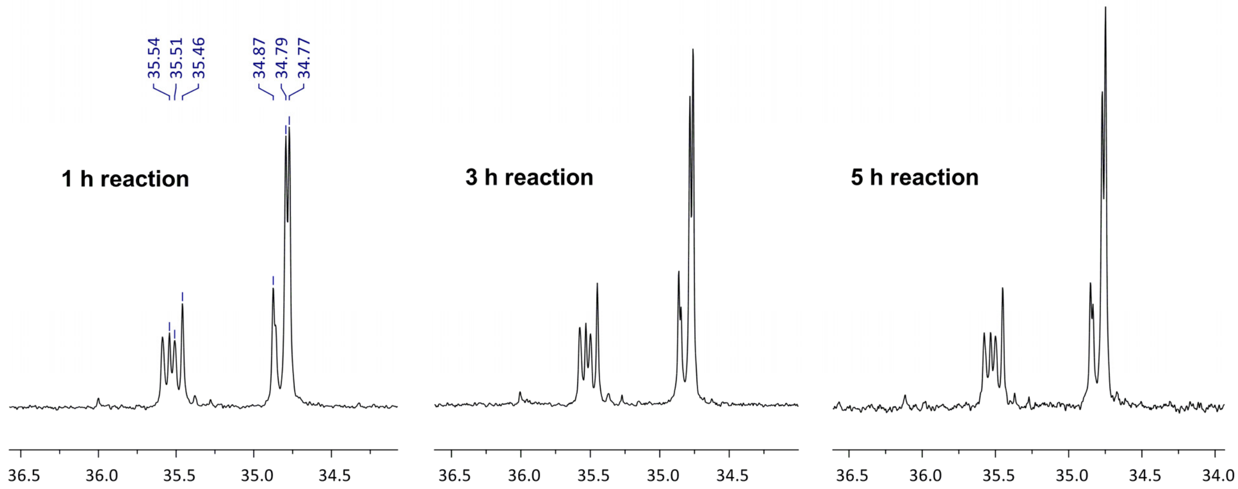

Figure S14. ^{31}P NMR spectra ($CDCl_3$, 162 MHz) of EtEP/εCL copolymerization probes after 1, 3 and 5 h (Table 4, Entries 5a–5c)

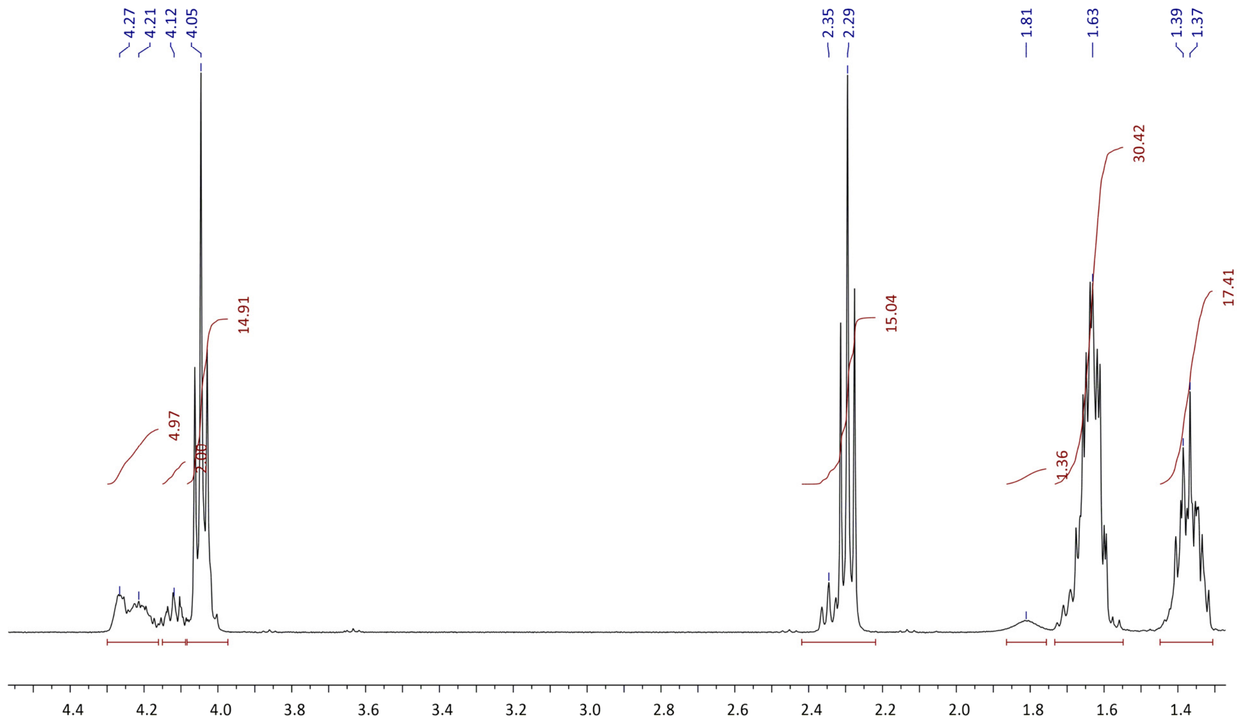

Figure S15. ^{1}H NMR spectrum ($CDCl_3$, 400 MHz) of EtOEP/εCL copolymer (Table 4, Entry 6c, re-precipitated from MeOH)

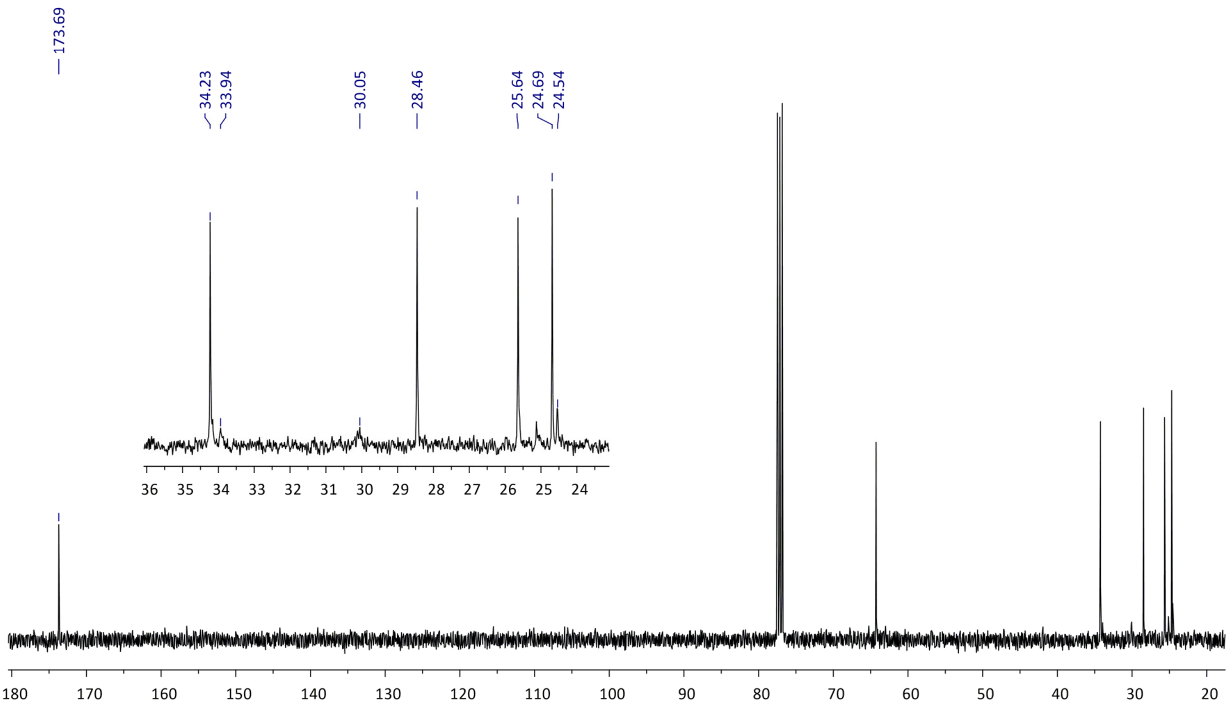

Figure S16. ^{13}C NMR spectrum ($CDCl_3$, 101 MHz) of EtOEP/εCL copolymer (Table 4, Entry 6c, re-precipitated from MeOH)

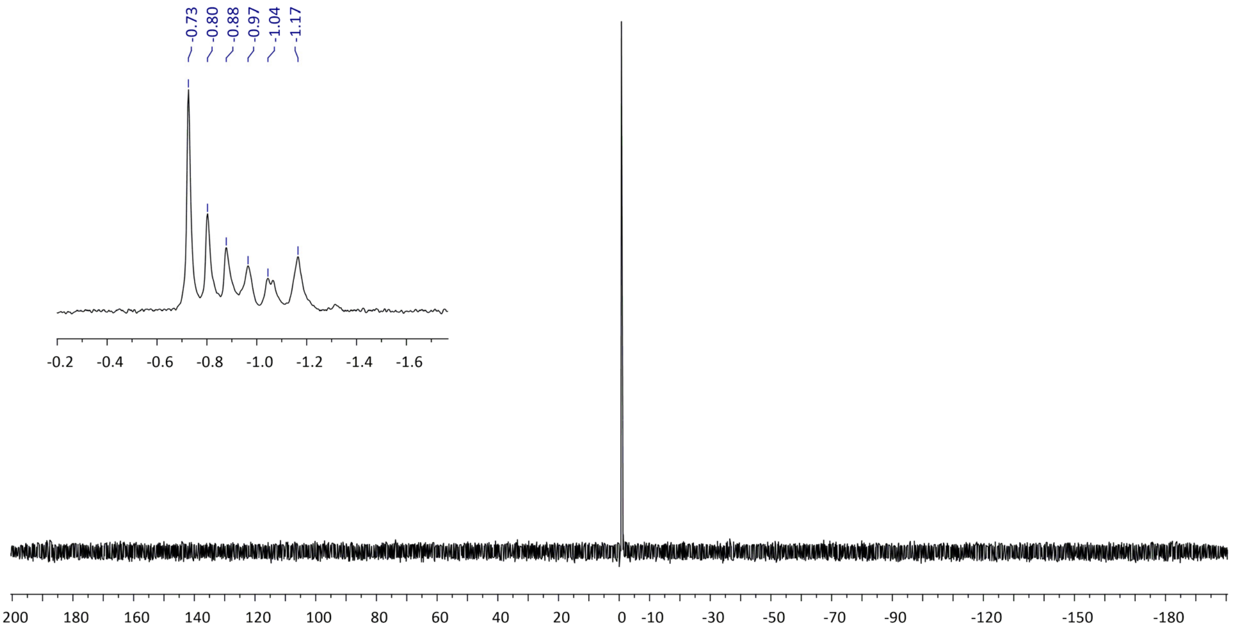

Figure S17. ^{31}P NMR spectrum ($CDCl_3$, 162 MHz) of EtOEP/εCL copolymer (Table 4, Entry 6c, re-precipitated from MeOH)
